# Treatment effects and safety of Chinese herbal medicine in gastroesophageal reflux disease: a systematic review of randomized controlled trials and a network meta-analysis with component analysis

**DOI:** 10.3389/fphar.2026.1920738

**Published:** 2026-09-24

**Authors:** Yi-Ting Chen, Yu-Hsuan Tai, Po-Yu Huang, Jung-Peng Chiu, Yu-Chiang Hung

**Affiliations:** 1 Linsen, Chinese Medicine and Kunming Branch, Taipei City Hospital, Taipei, Taiwan; 2 Graduate Institute of Clinical Medicine, Taipei Medical University, Taipei, Taiwan; 3 School of Chinese Medicine, College of Medicine, National Yang Ming Chiao Tung University, Taipei, Taiwan

**Keywords:** botanical drugs, Chinese herbal medicine, component network meta-analysis, gastroesophageal reflux disease, network meta-analysis, traditional Chinese medicine

## Abstract

**Background:**

Gastroesophageal reflux disease (GERD) is commonly treated with proton pump inhibitors (PPIs) despite their adverse effects and risk of recurrence. Chinese herbal medicine (CHM) has long been used to alleviate reflux-related symptoms and reduce relapse through multi-component and multi-target strategies. To evaluate the efficacy and safety of CHM for GERD and identify key botanical drugs contributing to therapeutic effects using a component-level analytical framework.

**Methods:**

English and Chinese databases were screened for relevant articles up to 15 April 2026. Eligible randomized controlled trials (RCTs) compared CHM with biomedicine. A meta-analysis was performed using RevMan 5.4 software, and the risk of bias assessed using Cochrane Risk of Bias two tool. A component-level contribution analysis based on component network meta-analysis (CNMA) framework and core-pattern network analysis were used to quantify individual botanical drug contributions and co-prescription structures.

**Results:**

Twenty randomized controlled trials involving 2,246 patients with GERD were included. Pairwise meta-analysis showed that CHM was superior to PPIs in clinical efficacy (odds ratio: 4.13, 95% confidence interval: 2.73–6.25) and symptom relief (standard mean difference: 0.71, 95% confidence interval: 0.49–0.94), with lower recurrence rates and equivalent safety. Network meta-analyses identified several CHM formulas with favorable effects relative to PPIs. Component-level contribution analyses identified varying associations among botanical drugs. Core-pattern analysis identified *Pinelliae rhizoma* as a highly connected botanical drug.

**Conclusion:**

This study presents the first component-level analysis of CHM prescriptions for GERD, suggesting improvements in clinical efficacy and recurrence control. By integrating prescription patterns with evidence-based pharmacology, our findings provide insights into multi-target CHM strategies and support the potential of CHM to strengthen the esophagogastric barrier and reduce long-term PPI dependence.

**Trial registration:**

The study was registered on PROSPERO, registration number CRD42024612778.

## Graphical Abstract

**Figure:**
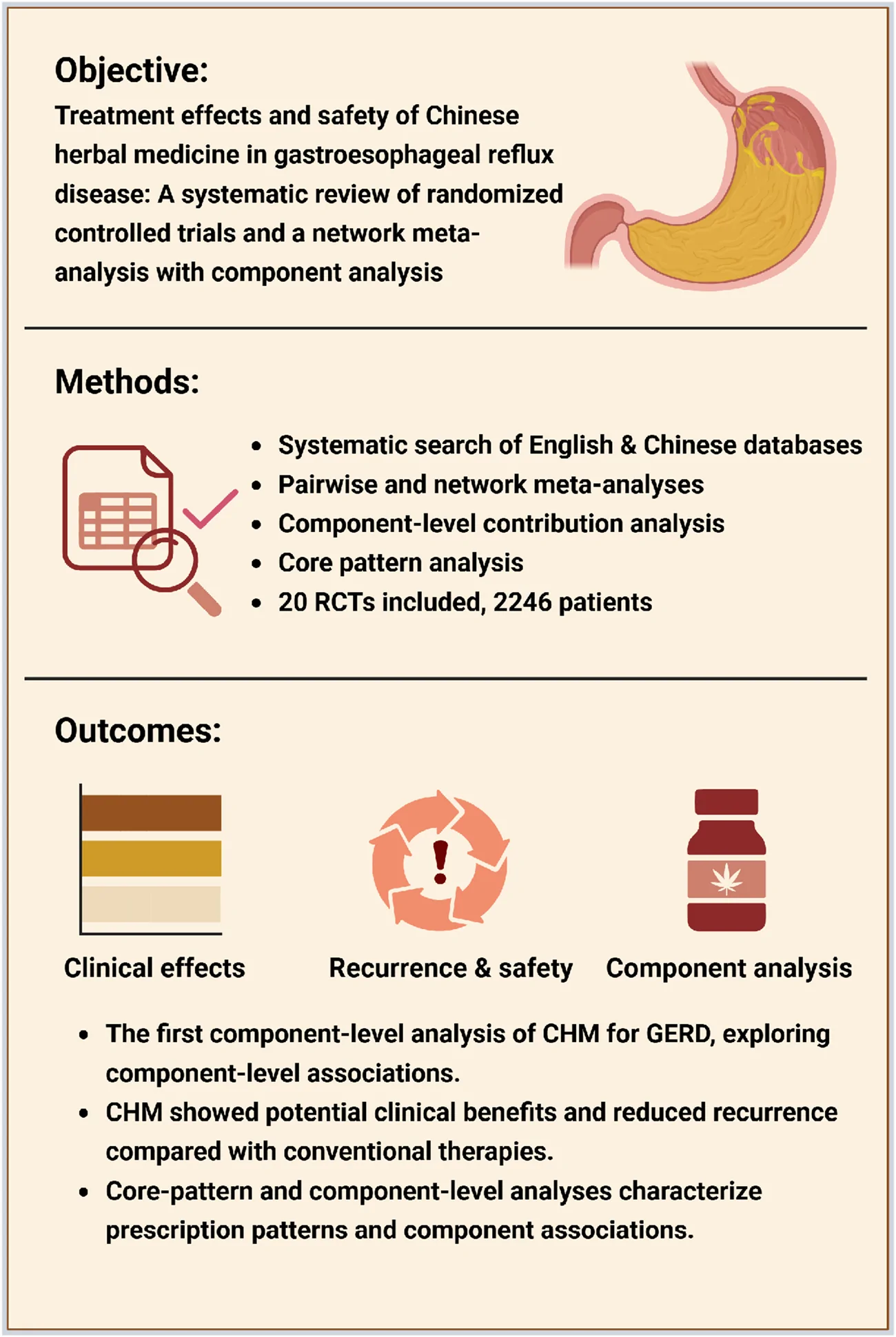
Created in BioRender. YiTing, C. (2026) https://BioRender.com/xwy5zz9.

## 1 Introduction

Gastroesophageal reflux disease (GERD) is a common digestive disorder in which gastric contents reflux into the esophagus, leading to symptoms and/or complications such as regurgitation, heartburn, and chest pain, with or without the presence of esophageal mucosal damage known as nonerosive reflux disease (NERD) (Katz et al., 2022; Dean et al., 2004). In adult populations in Western societies, the estimated prevalence of GERD is approximately 20% (Chhabra and Ingole, 2022).

In addition to gastric acid neutralizers, the primary GERD treatment is currently the administration of proton pump inhibitors (PPIs), including agents such as rabeprazole and omeprazole. PPIs suppress gastric acid secretion by irreversibly inhibiting hydrogen–potassium ATPase pumps in gastric parietal cells (Ward and Kearns, 2013). Although PPIs are effective for symptom control and mucosal healing, their long-term or recurrent use is often necessary. However, growing evidence indicates that long-term PPI exposure may be associated with adverse effects, such as renal disease, fracture risk, vitamin B12 and iron deficiency, and diarrhea or constipation (Fossmark et al., 2019; Begg et al., 2023). Patients with typical GERD symptoms may use on-demand or intermittent PPI treatment and should continue once-daily PPI even in the absence of symptoms due to an associated risk of esophagitis recurrence (Maret-Ouda et al., 2020).

Chinese herbal medicine (CHM) began to evolve approximately 3,000 years ago and was based on a balance of Yin–Yang and qi–blood (Zhang and Li, 2021). In recent years, an increasing number of clinical studies have indicated that CHM has several advantages over GERD. CHMs with various medicinal metabolites can alleviate GERD-related symptoms, reduce the incidence of adverse events, and reduce recurrence rates (Xiao et al., 2018; Mu et al., 2022; Jiang et al., 2023; Zhang et al., 2025). However, previous studies have mainly compared efficacy at the formula level without examining the contribution of individual botanical drugs. This limitation represents an important knowledge gap, given that CHM prescriptions are inherently multicomponent and multilevel interventions. Addressing this gap is particularly relevant in GERD, where the heterogeneity and recurrence of symptoms suggest the involvement of multiple pathological pathways. Therefore, the present study aimed to systematically review randomized controlled trials (RCTs) on CHM for the treatment of GERD. Network meta-analysis (NMA) was used to evaluate the efficacy of this treatment and identify key botanical drugs and prescription patterns through component-level and core-pattern analyses.

To our knowledge, this is the first study to integrate network meta-analysis with component-level contribution and core-pattern analyses to examine the therapeutic effects of CHM for GERD. This approach provides novel insights into the primary botanical drugs driving efficacy and provides an evidence-based foundation for the rational optimization of CHM in GERD management.

## 2 Methods

The current study was conducted in accordance with the Preferred Reporting Items for Systematic Reviews and Meta-Analyses (PRISMA) statement (Page et al., 2021), and it was registered on PROSPERO (registration number CRD42024612778). The original protocol specified a systematic review and meta-analysis; NMA was subsequently added because the eligible interventions formed a connected treatment network.

### 2.1 Search strategy

Nine literature databases, including six English-language databases (PubMed, MEDLINE [Ovid], Cochrane Controlled Register of Trials, EMBASE, CINAHL, and Scopus) and three Chinese-language databases (China National Knowledge Infrastructure [CNKI], Wanfang, and VIP), were searched for RCTs (including crossover trials) up to 15 April 2026. The search terms used were “GERD,” “acid reflux,” “herbal medic*,” “CHM,” and “herb*,” which were also revised according to the rules of the different databases. The detailed search strategies are listed in Supplementary Table S1.

### 2.2 Study selection

#### 2.2.1 Inclusion criteria

Studies that enrolled patients aged >18 years who met established guidelines or consensus criteria for GERD were included, with each study group comprising ≥15 patients. The intervention group received either CHM alone or a combination of CHM and biomedicine, whereas the comparator received either PPI alone or a combination of PPI with a CHM placebo or biomedicine (e.g., prokinetics). Eligible outcomes included clinical efficacy, symptom scores, recurrence, and adverse events, with no restriction on the timing of outcome assessment. Randomized controlled trials published in English or Chinese were eligible for inclusion.

#### 2.2.2 Exclusion criteria

Studies in which the CHM group received a combination of CHM with either acupoint injection or acupuncture, as well as studies without PPI control groups or those that included control participants who received acupoint injection or acupuncture, were excluded. Studies were also excluded if they only reported the overall effective rates and did not include symptom improvement outcomes.

### 2.3 Data extraction

Two independent reviewers (YTC and YHT) searched the aforementioned databases and screened the titles, abstracts, and full texts for studies that potentially satisfied the inclusion criteria. Potentially eligible studies were retrieved and considered for inclusion in the systematic review and meta-analysis, and discrepancies were resolved through discussion to reach a consensus. Quality assessment.

### 2.4 Quality assessment

Each RCT was assessed using the Cochrane Collaboration risk of bias tool which included five specific domains; randomization process, deviations from intended interventions, missing outcome data, measurement of the outcome, and selection of the reported result (Sterne et al., 2019). Overall risk was graded as low, some concern, or high according to the *Cochrane handbook* (Higgins et al., 2024).

### 2.5 Data analysis

#### 2.5.1 Pairwise meta-analysis

Primary outcomes were clinical efficacy and GERD symptom scores determined via various evaluation scales. Secondary outcomes were recurrence rates and safety. Differences between the intervention and control groups were assessed. Standard mean differences (SMDs) with 95% confidence intervals (CIs) and random-effects models based on the DerSimonian and Laird method were used to synthesize the GERD symptom rating scales for continuous data. For dichotomous data, treatment effects were assessed using odds ratios (ORs) with 95% CIs. Heterogeneity was investigated using the χ2 test and I2 statistics; sensitivity and subgroup analyses incorporating different scales were conducted to evaluate heterogeneity. A two-sided p-value of <0.05 was considered significant with respect to effect size estimation. Publication bias was evaluated using funnel plots and Egger’s test. Statistical analyses were performed using Review Manager 5.4 software provided by the Cochrane Collaboration. A free tool for data estimation and conversion for meta-analysis (DECoMA) (Chi et al., 2023) was used to calculate the standard deviation of the change in score using a correlation coefficient of 0.5, as recommended in the *Cochrane Handbook* (Higgins et al., 2024).

#### 2.5.2 Network meta-analysis

For advanced evidence synthesis, a frequentist network meta-analysis (NMA) was conducted using the *netmeta* package (version 3.2-0) in R (version 4.5.1; R Foundation for Statistical Computing, Vienna, Austria) (Balduzzi et al., 2023).

A random-effects model with between-study variance (τ^2^) estimated via the restricted maximum-likelihood (REML) method was employed to integrate both direct and indirect evidence, with PPIs as the reference treatment. Network consistency was examined through the node-splitting approach and design-by-treatment interaction model within the frequentist framework of the netmeta package. P-scores were used to rank the interventions and are the frequentist analogue of the surface under the cumulative ranking curve (SUCRA) used in Bayesian NMAs. Cumulative ranking probabilities were additionally estimated to characterize the distribution of possible treatment ranks. (Rücker and Schwarzer, 2015).

#### 2.5.3 Assessment of transitivity and consistency

The transitivity assumption was assessed by comparing potential clinical and methodological effect modifiers across treatment comparisons. Statistical consistency was further evaluated using the design-by-treatment interaction model and node-splitting where applicable (Higgins et al., 2024).

#### 2.5.4 Confidence assessment

The certainty of evidence in the NMA results was evaluated using the Confidence in Network Meta-Analysis (CINeMA) framework, which operationalizes the Grading of Recommendations Assessment, Development, and Evaluation (GRADE) principles for NMA. Six domains were assessed: within-study bias, cross-study bias (reporting bias), indirectness, imprecision, heterogeneity, and incoherence. Each treatment comparison in the network was assessed using the CINeMA web platform (Nikolakopoulou et al., 2020; Papakonstantinou et al., 2020). Evidence certainty was synthesized by integrating the primary study risk-of-bias with NMA estimates (SMD and 95% CI). Each domain was graded as “no concerns,” “some concerns,” or “major concerns,” and the overall confidence levels were rated as high, moderate, low, or very low according to the CINeMA framework (Nikolakopoulou et al., 2020).

#### 2.5.5 Component-level and core-pattern analyses

A component-level contribution analysis was conducted to estimate the associations of individual botanical drugs with the overall treatment effect. β coefficients with 95% CIs were estimated using an inverse-variance weighted additive linear model informed by the frequentist component network meta-analysis (CNMA) framework (Rücker et al., 2020), which models the overall effect as the additive combination of constituent components. This simplified regression-based approach was applied to the component effects and weighted by the inverse variance (1/SE^2^) of the pairwise effect estimates. Weighted bubble plots were used to visualize the relationships among the β estimates, frequency of appearance, and precision (1/SE). Core-pattern network analysis was conducted using the ggraph *h* (Pedersen, 2025) and *igraph* (Csardi and Nepusz, 2006) packages to identify the most frequently co-prescribed botanical drug combinations. Edge thickness represented co-occurrence frequency. Visualizations were generated primarily using the *ggplot2* package, with *igraph* and *ggraph* used for network visualization. (Wickham, 2016). The R script for the network meta-analysis, consistency assessment, component contribution analysis, and co-occurrence analysis is provided in the Supplementary Material.

## 3 Results

### 3.1 Study and patient characteristics

A total of 5,260 abstracts were reviewed and 1,617 duplicate publications were removed. Based on the titles and abstracts, 3,643 articles did not meet the selection criteria and were excluded, leaving 34 articles assessed for eligibility. Of these 34 articles, one was a protocol, five were duplicates, and nine were excluded because they did not include analyzable PPI or CHM intervention groups. The article selection process is summarized in Figure 1. Thus, 20 RCTs involving 2,246 patients with GERD were ultimately included in the analyses, based on the predetermined inclusion criteria (Cai Zongzong et al., 2024; Jia Ziliang et al., 2023; Lai Yaoliang, 2019; Li Z. et al., 2021; Li et al., 2022; Li Huili and Mou, 2023; Shih et al., 2019; Song et al., 2024; Takeuchi et al., 2019; Tominaga et al., 2012; Tominaga et al., 2014; Wang Guangming et al., 2020; Wang Jie, 2023; Yang Yunfeng et al., 2018; Yu Danchun et al., 2017; Zhang et al., 2021; Zhang et al., 2024; Zhang Hui-Kai and Zhang, 2021; Zhang Nan et al., 2024; Zhou Yan et al., 2022) The baseline characteristics of the included studies are presented in Table 1. All included studies were two-arm RCTs; 13 were “open-label” (Takeuchi et al., 2019; Tominaga et al., 2012; Song et al., 2024; Zhou Yan et al., 2022; Zhang Nan et al., 2024; Zhang Hui-Kai and Zhang, 2021; Li Huili and Mou, 2023; Lai Yaoliang, 2019; Yang Yunfeng et al., 2018; Wang Guangming et al., 2020; Cai Zongzong et al., 2024; Jia Ziliang et al., 2023; Yu Danchun et al., 2017) and seven were double-blind (Li et al., 2022; Li Z. et al., 2021; Shih et al., 2019; Tominaga et al., 2014; Zhang et al., 2021; Zhang et al., 2024; Wang Jie, 2023). Sixteen of the studies included were conducted in China (Li et al., 2022; Li Z. et al., 2021; Zhang et al., 2021; Song et al., 2024; Zhang et al., 2024; Zhou Yan et al., 2022; Zhang Nan et al., 2024; Zhang Hui-Kai and Zhang, 2021; Li Huili and Mou, 2023; Lai Yaoliang, 2019; Yang Yunfeng et al., 2018; Wang Guangming et al., 2020; Wang Jie, 2023; Cai Zongzong et al., 2024; Jia Ziliang et al., 2023; Yu Danchun et al., 2017), one in Taiwan (Shih et al., 2019), and three in Japan (Takeuchi et al., 2019; Tominaga et al., 2012; Tominaga et al., 2014), all of which were published between 2017 and 2024. Participant ages ranged from 18 to 79 years, and the mean duration of intervention was 6.45 weeks (range 2–8 weeks). The botanical drugs of the CHM formula are listed in Table 2. Detailed composition and preparation information for the included CHM interventions was compiled according to the ConPhyMP tool (Heinrich et al., 2022) based on information reported in the original studies and is provided in Supplementary Table S2. The included interventions comprised different preparation types, including decoctions, granules, and commercial products; detailed extract profiles and quality information were not available for all interventions. Botanical taxonomy, including scientific names, author citations, and families, was verified using the Medicinal Plant Names Services (MPNS) and Plants of the World Online (POWO). Valid source species are presented alongside the composition of each intervention in Supplementary Table S2, with detailed taxonomic information provided in the Appendix.

**FIGURE 1:**
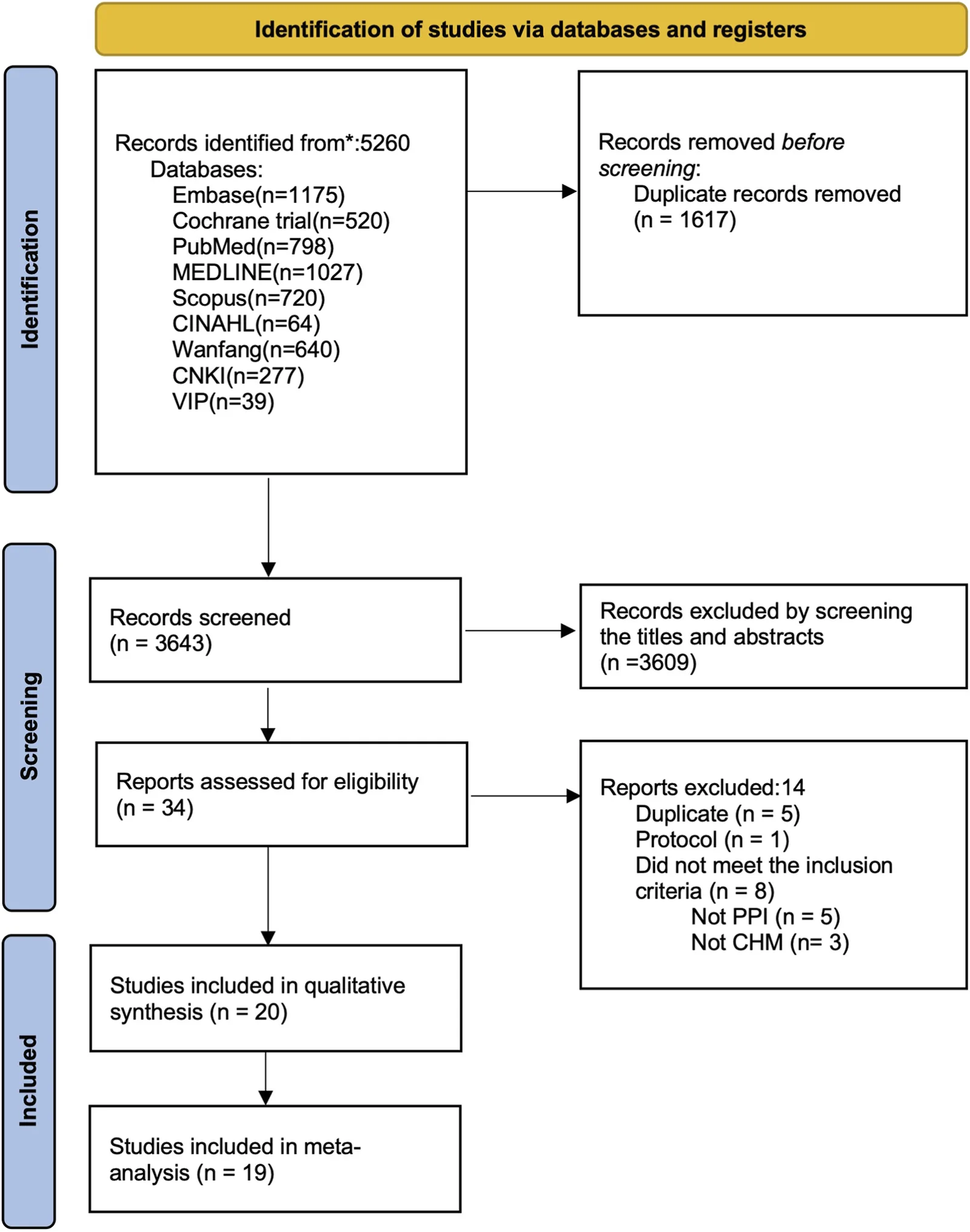
Flow diagram of the search strategy. Abbreviations: CHM, Chinese herbal medicine; PPI, proton pump inhibitor.

**TABLE 1:** Characteristics of the studies included in the analysis.

Authors, year	Sample size (M/F)	Age	Control	Intervention	Treatment duration
Cai Zongzong et al., 2024	C: 48 (27/21) I: 48 (25/23)	C: 51.19 ± 12.08 I: 52.68 ± 10.4	Esomeprazole magnesium (20 mg) + mosapride citrate (5 mg)	CHPLGML	8 weeks
Jia Ziliang et al., 2023	C: 33 (16/17) I: 39 (21/28)	C: 73.4 ± 4.8 70.1 ± 3.5	Any of the following PPIs: Omeprazole (20–60 mg), lansoprazole (15–30 mg), pantoprazole (40 mg), or rabeprazole (20 mg)	LZDBXHP	4 weeks then follow-up after 3 months
Lai Yaoliang, 2019	C: 30 (9/21) I: 30 (12/18)	C: 47.9 ± 12.4 I: 45.6 ± 13.2	Pantoprazole (40 mg)	Hejiang decoction	8 weeks
Li et al., 2021b	C: 130 (64/60) I: 116 (50/66)	C: 50.48 ± 11.71 I: 50.30 ± 10.38	Omeprazole (20 mg) + MXD placebo	MXD + omeprazole placebo	4 weeks, then follow-up after 1 and 3 months
Li F et al., 2022	C: 53 (16/37) I: 56 (12/44)	C: 48.51 ± 10.31 I: 48.05 ± 10.91	Dummy HWJND (81 g) + omeprazole (20 mg)	HWJND (81 g) + dummy omeprazole (20 mg)	8 weeks
Li Huili and Mou, 2023	C: 38 (22/16) I: 38 (20/18)	C: 49.12 ± 15.25 I: 48.89 ± 16.72	Rabeprazole (20 mg)	SNYLZS	8 weeks, then follow-up after 4 weeks
Shih YS et al., 2019	C: 37 (20/17) I: 40 (14/26)	C: 46.95 ± 13.42 I: 46.03 ± 13.88	Omeprazole (20 mg) + WCYT (3 g) placebo	WCYT (3 g) + omeprazole placebo	4 weeks
Song S et al., 2024	C: 38 (22/16) I: 43 (19/24)	C: 54.55 ± 11.68 I: 55.09 ± 14.19	PPI (rabeprazole, lansoprazole, esomeprazole, or omeprazole) twice-daily dosing + magnesium aluminum carbonate (0.5 g)	BXHP (57 g/200 mL)	2 weeks
Takeuchi T et al., 2019	C: 33 (11/22) I: 38 (10/28)	C: 60.8 ± 14.7 I: 59.2 ± 14.7	Rabeprazole (20 mg)	Rabeprazole (10 mg/day) + HST (7.5 g/day)	4 weeks
Tominaga K et al., 2012	C: 51 (17/34) I: 50 (20/30)	C: 63.6 (25–86) I: 64.5 (25–90)	Rabeprazole (20 mg)	Rabeprazole (10 mg/day) + rikkunshito (7.5 g/day)	4 weeks
Tominaga K et al., 2014	C: 108 (28/80) I: 109 (35/74)	C: 59.4 (22–83) I: 62.1 (25–85)	Rabeprazole (10 mg once daily) + placebo (7.5 g/day 3 times)	Rabeprazole (10 mg once daily) + rikkunshito (7.5 g/day 3 times)	8 weeks
Wang Guangming et al., 2020	C: 60 (36/24) I: 60 (37/23)	C: 54.62 ± 9.59 I: 53.66 ± 9.27	Rabeprazole (20 mg)	SGWA	8 weeks
Wang Jie, 2023	C: 35 (14/21) I: 36 (12/24)	C: 49.80 ± 13.10 I: 49.50 ± 12.50	Rabeprazole (20 mg)	CSTG	8 weeks
Yang Yunfeng et al., 2018	C: 42 (21/21) I: 43 (19/24)	C: 49.19 ± 11.75 I: 46.63 ± 13.80	Omeprazole (20 mg)	HZJP	8 weeks
Yu Danchun et al., 2017	C: 40 (24/16) I: 40 (28/12)	C: 47.62 ± 9.74 I: 48.71 ± 10.18	Rabeprazole (10 mg) + mosapride citrate (5 mg)	BXHP	8 weeks
Zhang J et al., 2021	C: 98 (31/66) I: 98 (40/58)	C: 46.54 ± 15.07 I: 45.45 ± 14.03	Dummy JQ (34.8 g) + omeprazole (20 mg)	JQ (34.8 g) + omeprazole (10 mg) + dummy omeprazole (10 mg)	4 weeks, then follow-up after 2 weeks
Zhang X et al., 2024	C: 132 (73/59) I: 132 (71/61)	C: 51.29 ± 12.00 I: 53.32 ± 12.54	Placebo SGHW (20 mg) + rabeprazole (20 mg)	SGHWX (20 mg) + rabeprazole (10 mg)	8 weeks, then follow-up 2 weeks
Zhang HK et al., 2021	C: 42 (24/18) I: 42 (26/16)	C: 48.54 ± 6.72 I: 49.16 ± 6.81	Omeprazole (20 mg)	ZBXHP	3 weeks
Zhang N et al., 2024	C: 40 (17/23) I: 40 (18/22)	C: 48.77 ± 11.96 I: 44.35 ± 14.25	Esomeprazole magnesium (40 mg) + mosapride citrate (5 mg)	SGHW	8 weeks
Zhou Yan et al., 2022	C: 30 (13/17) I: 30 (12/18)	C: 53.2 ± 10.4 I: 54.4 ± 10.8	Rabeprazole (20 mg)	JZJN	4 weeks

Abbreviation: M/F: Male/Female; C/I: Control group/Intervention group.

**TABLE 2:** Botanical drugs in Chinese medicine formulas.

Authors, year	Formula	Botanical drugs
Cai Zongzong et al., 2024	Chaihu plus longgu muli (CHPLGML) decoction	*Gardeniae Fructus* (梔子 zhī zǐ) 10g, *Citri Reticulatae Pericarpium* (陳皮 chén pí) 10g, *Paeoniae Radix Rubra* (赤芍 chì sháo) 10g, *Chuanxiong Rhizoma* (川芎 chuān xiōng) 10g, *Bupleuri Radix* (柴胡 chái hú) 9g, *Scutellariae Radix* (黃芩 huáng qín) 6g, *Fossilia Ossis Mastodi* (龍骨 lóng gǔ) 6g, *Ostreae Concha* (牡蠣 mǔ lì) 6g, *Cyperi Rhizoma Praeparatum* (香附 zhì xiāng fù) 6g, *Pinelliae Rhizoma* (半夏 bàn xià) 6g, *Glycyrrhizae Radix et Rhizoma* (生甘草 shēng gān cǎo) 6g, *Aurantii Fructus* (枳殼 zhǐ ké) 6g, and *Evodiae Fructus* (吳茱萸 wú zhū yú) 3 g
Jia Ziliang et al., 2023	Modified lizhong decoction combined with banxia houpu (LZDBXHP) decoction	*Codonopsis Radix* (黨參 dǎng shēn) 15g, *Atractylodis Macrocephalae Rhizoma* (raw) (生白朮 shēng bái zhú) 20g, *Zingiberis Rhizoma* (乾薑 gān jiāng) 10g, *Pinelliae Rhizoma Praeparatum* (法半夏 fǎ bàn xià) 15g, *Poria* (茯苓 fú líng) 10g, *Magnoliae Officinalis Cortex* (厚朴 hòu pò) 10g, *Cimicifugae Rhizoma* (升麻 shēng má) 6g, *Sepiae Endoconcha* (海螵蛸 hǎi piāo xiāo) 20g, *Hordei Fructus Germinatus Praeparatus* (焦麥芽 jiāo mài yá) 15g, *Glycyrrhizae Radix et Rhizoma Praeparata cum Melle* (炙甘草 zhì gān cǎo) 10 g
Lai Yaoliang, 2019	Hejiang therapy	*Bupleuri Radix* (柴胡 chái hú) 10g, *Paeoniae Radix Alba* (白芍 bái sháo) 10g, *Aurantii Fructus* (枳殼 zhǐ ké) 10g, *Pinelliae Rhizoma Praeparatum* (清半夏 qīng bàn xià) 9g, *Cyperi Rhizoma Praeparatum* (香附 zhì xiāng fù) 10g, *Perillae Caulis* (紫蘇梗 zǐ sū gěng) 10g, *Magnoliae Officinalis Cortex* (厚朴 hòu pò) 10g, *Coptidis Rhizoma* (黃連 huáng lián) 6g, and *Evodiae Fructus* (吳茱萸 wú zhū yú) 3 g
Li et al., 2021b	Modified xiaochaihu decoction (MXD)	*Bupleuri Radix* (柴胡 chái hú) 10g, *Codonopsis Radix* (黨參 dǎng shēn) 15g, *Atractylodis Macrocephalae Rhizoma Praeparatum* (炒白朮 chǎo bái zhú) 12g, *Coptidis Rhizoma* (黃連 huáng lián) 10g, *Inulae Flos* (旋覆花 xuán fù huā) 10g, and *Raphani Semen Praeparatum* (炒萊菔子 chǎo lái fú zǐ) 15 g
Li F et al., 2022	Hewei jiangni decoction (HWJND)	*Scutellariae Radix* (黃芩 huáng qín) 9g, *Coptidis Rhizoma* (黃連 huáng lián) 6g, *Zingiberis Rhizoma Recens* (Raw) (生薑 shēng jiāng) 9g, *Pinelliae Rhizoma* (半夏 bàn xià) 9g, *Fritillariae Thunbergii Bulbus* (浙貝母 zhè bèi mǔ) 9g, *Taraxaci Herba* (蒲公英 pú gōng yīng) 9g, *Gentianae Radix* (龍膽 lóng dǎn) 9g, *Aurantii Fructus Immaturus* (枳實 zhǐ shí) 9g, *Trichosanthis Radix* (栝蔞根 guā lóu gēn) 9g, and *Glycyrrhizae Radix et Rhizoma* (甘草 gān cǎo) 3 g
Li Huili and Mou, 2023	Siniyulianzheshi (SNYLZS) decoction	*Bupleuri Radix Praeparata cum Aceto* (醋柴胡 cù chái hú) 12g, *Aurantii Fructus Immaturus Praeparatus* (炒枳實 chǎo zhǐ shí) 15g, *Paeoniae Radix Rubra* (赤芍 chì sháo) 30g, *Glycyrrhizae Radix et Rhizoma* (甘草 gān cǎo) 9g, *Coptidis Rhizoma Praeparata* (炒黃連 chǎo huáng lián) 9g, *Evodiae Fructus* (吳茱萸 wú zhū yú) 3g, *Inulae Flos* (旋覆花 xuán fù huā) 15g, and *Hematitum Crudum* (生赭石 shēng zhě shí) 30 g
Shih YS et al., 2019	Wu chu yu tang (WCYT)	*Evodiae Fructus* (吳茱萸 wú zhū yú), *Ginseng Radix et Rhizoma* (人參 rén shēn), *Jujubae Fructus* (大棗 dà zǎo), and *Zingiberis Rhizoma Recens* (生薑 shēng jiāng)
Song S et al., 2024	Banxia houpu (BXHP) decoction	*Pinelliae Rhizoma Praeparatum cum Zingibere* (薑半夏 jiāng bàn xià) 15g, *Magnoliae Officinalis Cortex* (厚朴 hòu pò) 9g, *Poria* (茯苓 fú líng) 12g, *Zingiberis Rhizoma Recens* (生薑 shēng jiāng) 15g, and *Perillae Folium* (紫蘇葉 zǐ sū yè) 6 g
Takeuchi T et al., 2019	Hangeshashinto (HST)	*Pinelliae Rhizoma* (半夏 bàn xià), *Jujubae Fructus* (大棗 dà zǎo), *Ginseng Radix et Rhizoma* (人參 rén shēn), *Zingiberis Rhizoma Praeparatum* (炮薑 pào jiāng), *Coptidis Rhizoma* (黃連 huáng lián), *Scutellariae Radix* (黃芩 huáng qín), and *Glycyrrhizae Radix et Rhizoma* (甘草 gān cǎo)
Tominaga K et al., 2012	Rikkunshito	*Glycyrrhizae Radix et Rhizoma* (甘草 gān cǎo), *Zingiberis Rhizoma* (乾薑 gān jiāng), *Atractylodis Rhizoma* (蒼朮 cāng zhú), *Jujubae Fructus* (大棗 dà zǎo), *Citri Reticulatae Pericarpium* (陳皮 chén pí), *Ginseng Radix et Rhizoma* (人參 rén shēn), *Pinelliae Rhizoma* (半夏 bàn xià), and *Poria* (茯苓 fú líng)
Tominaga K et al., 2014	Rikkunshito	*Glycyrrhizae Radix et Rhizoma* (甘草 gān cǎo), *Zingiberis Rhizoma* (乾薑 gān jiāng), *Atractylodis Rhizoma* (蒼朮 cāng zhú), *Jujubae Fructus* (大棗 dà zǎo), *Citri Reticulatae Pericarpium* (陳皮 chén pí), *Ginseng Radix et Rhizoma* (人參 rén shēn), *Pinelliae Rhizoma* (半夏 bàn xià), and *Poria* (茯苓 fú líng)
Wang Guangming et al., 2020	Shugan weian (SGWA) recipe	*Pinelliae Rhizoma Praeparatum* (法半夏 fǎ bàn xià) 10g, *Zingiberis Rhizoma* (乾薑 gān jiāng) 3g, *Evodiae Fructus* (吳茱萸 wú zhū yú) 1g, *Codonopsis Radix* (黨參 dǎng shēn) 10g, *Poria* (茯苓 fú líng) 15g, *Atractylodis Macrocephalae Rhizoma Praeparata* (炒白朮 chǎo bái zhú) 10g, *Bupleuri Radix* (柴胡 chái hú) 10g, *Corydalis Rhizoma* (延胡索 yán hú suǒ) 10g, *Aucklandiae Radix Praeparatum* (煨木香 wēi mù xiāng) 6g, *Bambusae Caulis in Taeniam* (竹茹 zhú rú) 10g, *Inulae Flos* (旋覆花 xuán fù huā) 15g, *Coptidis Rhizoma* (黃連 huáng lián) 3 g
Wang Jie, 2023	Chaishao tongjian (CSTG) prescription	*Bupleuri Radix* (柴胡 chái hú) 9g, *Paeoniae Radix Alba* (白芍 bái sháo) 12g, *Coptidis Rhizoma* (黃連 huáng lián) 3g, *Evodiae Fructus* (吳茱萸 wú zhū yú) 3g, *Rehmanniae Radix* (生地 shēng dì) 9g, *Achyranthis Bidentatae Radix* (牛膝 niú xī) 9g, *Aurantii Fructus* (枳殼 zhǐ ké) 12g, *Arcae Concha Praeparata* (煅瓦楞子 duàn wǎ léng zi) 27g, *Pinelliae Rhizoma* (半夏 bàn xià) 6g, *Magnoliae Officinalis Cortex* (厚朴 hòu pò) 12g, *Atractylodis Macrocephalae Rhizoma Praeparata* (炒白朮 chǎo bái zhú) 12g, and *Pseudostellariae Radix* (太子參 tài zǐ shēn) 12 g
Yang Yunfeng et al., 2018	Hezhong jianpi (HZJP) decoction	*Atractylodis Macrocephalae Rhizoma* (白朮 bái zhú) 9g, *Eriobotryae Folium* (枇杷葉 pí pá yè) 9g, and *Platycodonis Radix* (桔梗 jí gěng) 1 g
Yu Danchun et al., 2017	Banxia houpu (BXHP) decoction	*Pinelliae Rhizoma* (半夏 jiāng bàn xià), *Magnoliae Officinalis Cortex* (厚朴 hòu pò), *Poria* (茯苓 fú líng), *Zingiberis Rhizoma Recens* (生薑 shēng jiāng), and *Perillae Folium* (紫蘇葉 zǐ sū yè)
Zhang J et al., 2021	JianpiQinghua granule (JQ granule)	*Codonopsis Radix* (黨參 dǎng shēn) 18 g, *Atractylodis Rhizoma* (蒼朮 cāng zhú) 15 g, *Perillae Folium* (紫蘇葉 zǐ sū yè) 12 g, *Eupatorii Herba* (佩蘭 pèi lán) 12 g, *Aurantii Fructus* (枳殼 zhǐ qiào) 12 g, *Scutellariae Radix* (黃芩 huáng qín) 9 g, *Coptidis Rhizoma* (黃連 huáng lián) 6 g, *Sepiae Endoconcha* (海螵蛸 hǎi piāo xiāo) 30 g, *Massa Medicata Fermentata* (神麯 shén qū) 15 g, and *Amomi Fructus Rotundus* (豆蔻 dòu kòu) 6 g
Zhang X et al., 2024	Shugan hewei (SGHWX) granules	*Ziziphi Spinosae Semen* (酸棗仁 suān zǎo rén), *Magnoliae Officinalis Cortex* (厚朴 hòu pò), *Coptidis Rhizoma* (黃連 huáng lián), *Evodiae Fructus* (吳茱萸 wú zhū yú), *Zingiberis Rhizoma* (乾薑 gān jiāng), *Arcae Concha* (瓦楞子 wǎ léng zi), *Inulae Flos* (旋覆花 xuán fù huā), *Bupleuri Radix* (柴胡 chái hú), *Corydalis Rhizoma* (延胡索 yán hú suǒ), *Cyperi Rhizoma* (香附 xiāng fù), *Gardeniae Fructus* (梔子 zhī zǐ), *Aurantii Fructus* (枳殼 zhǐ ké), and *Polygonati Rhizoma* (黃精 huáng jīng)
Zhang HK et al., 2021	Banxia houpu (ZBXHP) decoction	*Pinelliae Rhizoma* (半夏 bàn xià) 15g, *Magnoliae Officinalis Cortex* (厚朴 hòu pò) 15g, *Poria* (茯苓 fú líng) 12g, *Cyperi Rhizoma* (香附 xiāng fù) 10g, *Perillae Folium* (紫蘇葉 zǐ sū yè) 9g, *Zingiberis Rhizoma Recens* (生薑 shēng jiāng) 9g, *Citri Reticulatae Pericarpium* (陳皮 chén pí) 9g, and *Glycyrrhizae Radix et Rhizoma* (甘草 gān cǎo) 3 g
Zhang N et al., 2024	Shugan hewei (SGHW) formula	*Bupleuri Radix* (柴胡 chái hú) 9 g, *Cyperi Rhizoma* (香附 xiāng fù) 12 g, *Aurantii Fructus* (枳殼 zhǐ qiào) 12 g, *Inulae Caulis* (旋覆梗 xuán fù gěng) 12 g, *Hematitum Crudum* (代赭石 dài zhě shí) 15 g, *Coptidis Rhizoma* (黃連 huáng lián) 3 g, *Evodiae Fructus* (吳茱萸 wú zhū yú) 3 g, *Crataegi Fructus Praeparatus* (焦山楂 jiāo shān zhā) 9 g, *Arcae Concha Praeparata* (煅瓦楞 duàn wǎ léng) 30 g, *Polygonati Rhizoma* (黃精 huáng jīng) 15 g, *Fritillariae Thunbergii Bulbus* (浙貝母 zhè bèi mǔ) 9 g, *Magnoliae Officinalis Cortex* (厚朴 hòu pò) 12 g, and *Zingiberis Rhizoma Recens* (生薑 shēng jiāng) 3 g
Zhou Yan et al., 2022	JianZhong jiangni (JZJN) decoction	*Cynanchi Paniculati Radix et Rhizoma* (徐長卿xú cháng qīng) 30g, *Arcae Concha Praeparata* (煅瓦楞 duàn wǎ léng) 30g, *Ostreae Concha Praeparata* (煅牡蠣 duàn mǔ lì) 30g, *Sepiae Endoconcha* (海螵蛸 hǎi piāo xiāo) 30g, *Codonopsis Radix Praeparata* (炒黨參 chǎo dǎng shēn) 20g, *Corydalis Rhizoma* (延胡索 yán hú suǒ) 20g, *Atractylodis Macrocephalae Rhizoma Praeparata* (炒白朮 chǎo bái zhú) 10g, *Poria* (茯苓 fú líng) 10g, *Pinelliae Rhizoma Praeparatum cum Zingibere* (薑半夏 jiāng bàn xià) 10g, *Paeoniae Radix Alba* (白芍 bái sháo) 10g, *Cyperi Rhizoma Praeparatum* (制香附 zhì xiāng fù) 10g, *Inulae Flos* (旋覆花 xuán fù huā) 10g, *Dalbergiae Odoriferae Lignum* (降香 jiàng xiāng) 5g, and *Glycyrrhizae Radix et Rhizoma* (生甘草 shēng gān cǎo) 3 g

### 3.2 Evaluation of outcome measures

For domain 1 (randomization process), “some concerns” were noted in eight trials (Li Huili and Mou, 2023; Lai Yaoliang, 2019; Song et al., 2024; Zhang Hui-Kai and Zhang, 2021; Zhou Yan et al., 2022; Wang Guangming et al., 2020; Cai Zongzong et al., 2024; Jia Ziliang et al., 2023), due to the lack of information on allocation concealment, and the remaining studies were judged as low risk. For domain 2 (deviations from intended interventions), “some concerns” were identified in 13 studies because of the open-label design or insufficient information regarding blinding and potential deviations from the intended interventions arising from the trial context (Takeuchi et al., 2019; Tominaga et al., 2012; Song et al., 2024; Li Huili and Mou, 2023; Lai Yaoliang, 2019; Zhang Hui-Kai and Zhang, 2021; Zhang Nan et al., 2024; Zhou Yan et al., 2022; Wang Guangming et al., 2020; Wang Jie, 2023; Cai Zongzong et al., 2024; Jia Ziliang et al., 2023; Yu Danchun et al., 2017). or domain 3 (missing outcome data), one (Li Z. et al., 2021) reported a large number of withdrawals because of participant dissatisfaction and was assessed as “some concerns.” For domain 5 (selection of the reported result), “some concerns” were identified in eight trials because insufficient information was available to determine whether the reported results could have been selected from multiple eligible outcome measurements. Where prospective study plans were available, prespecified outcome measurements and analyses were compared with those reported in the published articles. The risk assessments in these studies are summarized in Figure 2.

**FIGURE 2:**
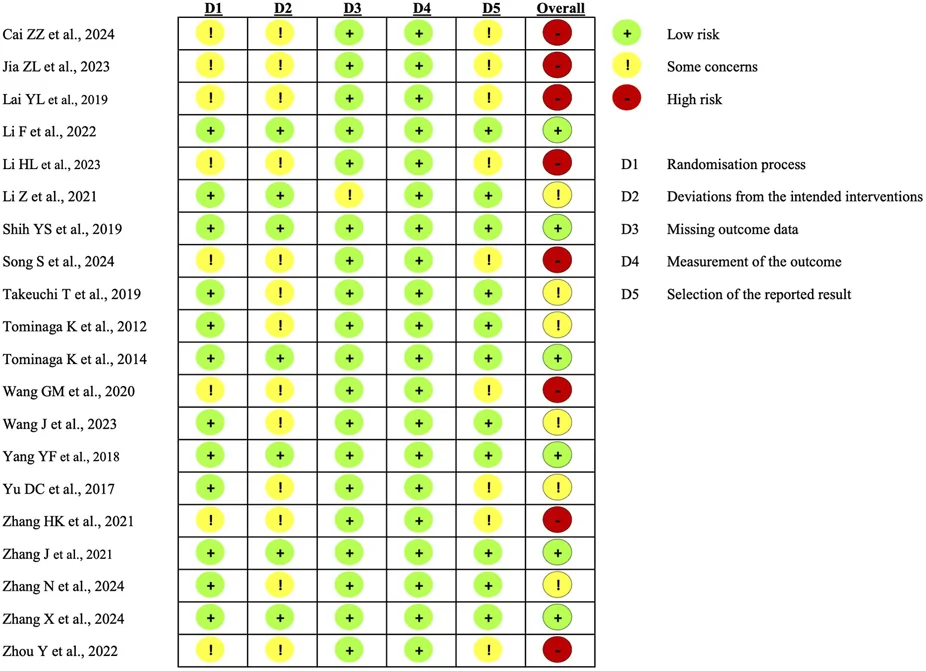
Risk of bias analysis of the included studies.

### 3.3 Clinical effects

Fourteen of the 20 RCTs (Li Z. et al., 2021; Zhang et al., 2021; Zhang et al., 2024; Yu Danchun et al., 2017; Lai Yaoliang, 2019; Zhou Yan et al., 2022; Cai Zongzong et al., 2024; Jia Ziliang et al., 2023; Zhang Nan et al., 2024; Li Huili and Mou, 2023; Wang Guangming et al., 2020; Wang Jie, 2023; Yang Yunfeng et al., 2018; Zhang Hui-Kai and Zhang, 2021), involving 778 patients in the CHM group and 782 in the control group, were included in the pairwise meta-analysis evaluating overall clinical efficacy. The pooled analysis revealed a significantly higher clinical efficacy rate in the CHM group than in the control group (OR = 4.13, 95% CI: 2.73–6.25), and pooled results indicated an overall Z effect of 6.70 (p < 0.00001), with moderate heterogeneity (p = 0.03, I2 = 46%; Figure 3A).

**FIGURE 3:**
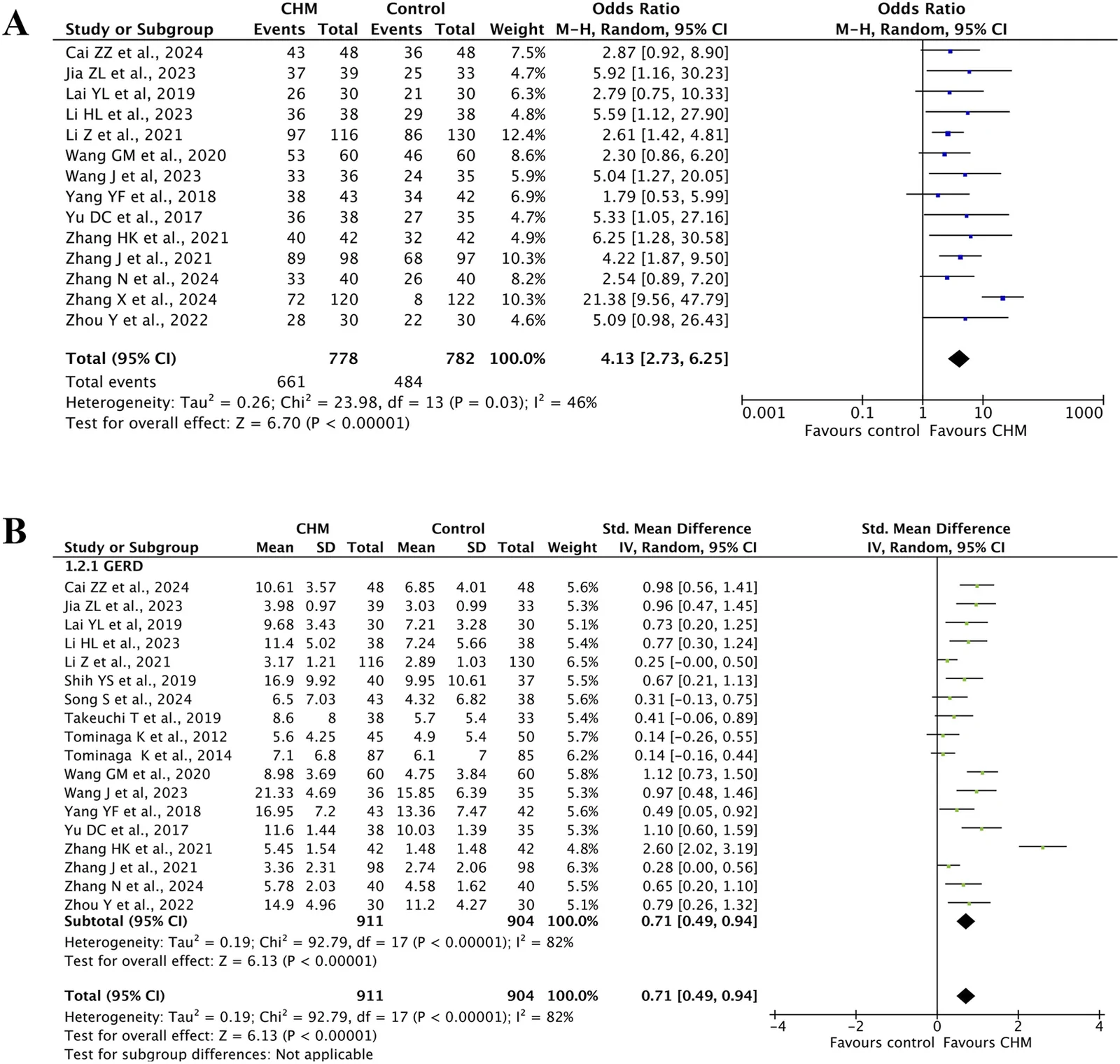
**(A)** Forest plot of the clinical effects of CHM treatment on GERD. **(B)** Forest plot of CHM treatment effects on GERD symptom scores. Abbreviations: CHM, Chinese herbal medicine; GERD, gastroesophageal reflux disease.

### 3.4 Comparisons of GERD symptom scores

A pairwise meta-analysis was performed to compare the changes in GERD symptom severity between the CHM and control groups. Eighteen of the 20 RCTs assessed GERD symptom severity using various validated scales including the Frequency Scale for the Symptoms of GERD (FSSG), Reflux Diagnostic Questionnaire (RDQ), and GERD-Q. Two RCTs were excluded due to insufficient data (Li et al., 2022; Zhang et al., 2024). The pooled results (Figure 3B) demonstrated a significant difference between groups, favoring the CHM group over the control group (SMD = 0.71, 95% CI: 0.49–0.94), indicating an overall Z effect of 6.70 (p < 0.00001) with considerable heterogeneity (p < 0.00001, I^2^ = 82%).

### 3.5 Recurrence

Among the included studies, follow-up evaluations were performed in six RCTs to assess post-treatment recurrence of symptoms (Li Z. et al., 2021; Zhang et al., 2021; Zhang et al., 2024; Li Huili and Mou, 2023; Jia Ziliang et al., 2023; Wang Jie, 2023). Li et al. (Li Z. et al., 2021) reported that symptom recurrence rate was significantly higher in the control group at both 1 month and 3 months after completing treatment. Zhang et al. (Zhang et al., 2024) found that recurrence within 2 weeks post-treatment was significantly higher in the control group than in the CHM group. Zhang et al. (Zhang et al., 2021) observed no significant differences in symptom relief between groups after 2 weeks of follow-up; however, the CHM group demonstrated greater improvements in anxiety scores at week 4 and in physical and social functioning up to week 6, although these differences were no longer evident at week 8, except for quality-of-life outcomes. According to Li HL et al. (Li Huili and Mou, 2023), follow-up endoscopy 1 month after treatment completion showed a significantly lower esophageal mucosal inflammation score in the CHM group than in the control group (p < 0.05). Similarly, Jia et al. (Jia Ziliang et al., 2023) and Wang et al. (Wang Jie, 2023) reported that the recurrence rate in the treatment group was significantly lower than that in the control group (16.67% [2 of 12] vs. 33.33% [2 of 6]; 13.8% [5 of 36] vs. 28.57% [10 of 35]; P < 0.05).

Overall, these findings suggest that CHM interventions may help reduce recurrence rates and sustain therapeutic effects after treatment completion, although follow-up durations varied across studies.

### 3.6 Safety

Among the included studies, 12 RCTs (Shih et al., 2019; Takeuchi et al., 2019; Tominaga et al., 2012; Zhang et al., 2021; Zhang et al., 2024; Li et al., 2022; Tominaga et al., 2014; Wang Guangming et al., 2020; Zhou Yan et al., 2022; Zhang Hui-Kai and Zhang, 2021; Jia Ziliang et al., 2023; Li Huili and Mou, 2023) evaluated safety and adverse effects. Regarding laboratory assessments, five studies (Li et al., 2022; Shih et al., 2019; Takeuchi et al., 2019; Zhang et al., 2024; Li Huili and Mou, 2023) howed no significant differences in aspartate aminotransferase, alanine aminotransferase, blood urea nitrogen, serum creatinine, white blood cells, red blood cells, platelet counts, urinalysis, or electrocardiography between the CHM and control groups. No serious adverse effects were observed in most trials; minor adverse effects, such as nausea, vomiting, dizziness, urinary tract infection, and diarrhea, were reported in several studies (Shih et al., 2019; Takeuchi et al., 2019; Tominaga et al., 2014; Zhang et al., 2021; Zhou Yan et al., 2022; Zhang Hui-Kai and Zhang, 2021; Wang Guangming et al., 2020; Li Huili and Mou, 2023; Jia Ziliang et al., 2023). One serious adverse event, dizziness, was reported and judged as not treatment-related (Tominaga et al., 2014). One patient was excluded from the clinical trial because of a suspected drug-related adverse reaction (Zhang et al., 2024). Overall, the incidence of adverse events was low, with no evidence of treatment-related toxicity or organ impairment across studies, indicating good tolerability and no major safety concerns for CHM interventions.

### 3.7 Subgroup analysis

Subgroup analyses were performed to explore the potential sources of heterogeneity. CHM consistently showed superior efficacy compared to controls across different outcome measurement scales (RDQ, GERD-Q, and FSSG) and GERD subtypes (mixed-type GERD vs. NERD). Substantial heterogeneity was observed in GERD-Q subgroup (I^2^ = 95%), whereas heterogeneity was minimal in both the FSSG and RDQ subgroups (I^2^ = 0%). When stratified by comparator type, studies using PPI plus mosapride as the control showed a larger effect size in favor of CHM (Z = 6.74); however, heterogeneity in the PPI subgroup remained considerable (I^2^ = 83%). These findings suggest that none of the examined factors fully accounted for the observed variability (Supplementary Figures S1–S3). Variations in PPI type, dose, and treatment duration across studies were further examined and are summarized in Supplementary Table S3. Exploratory subgroup analyses according to outcome instrument and treatment duration showed no significant between-subgroup differences (P = 0.25 and P = 0.51, respectively; Supplementary Figures S4–S5).

### 3.8 Publication bias

Publication bias was assessed using a funnel plot and Egger’s regression test. Although Egger’s test indicated significance (t = 3.7, p = 0.0019), the funnel plot appeared generally symmetrical, except for one study with a comparatively high effect size, which may partially explain the asymmetry (Supplementary Figure S6). According to the *Cochrane Handbook* (version 6.5, Section 13.3.5), funnel plot asymmetry should not be regarded as a diagnostic of non-reporting bias, as such patterns may also result from true heterogeneity or methodological variation. Therefore, the observed asymmetry should be interpreted with caution, considering potential reporting biases (Higgins et al., 2024).

### 3.9 Network meta-analysis

A frequentist random-effects NMA was conducted using the netmeta package. The treatment network comprised 17 interventions, with PPI serving as a common comparator (Figure 4A). Each node represents a treatment, and the size of the node is proportional to the number of patients assigned, whereas the line width corresponds to the number of direct comparisons between interventions.

**FIGURE 4:**
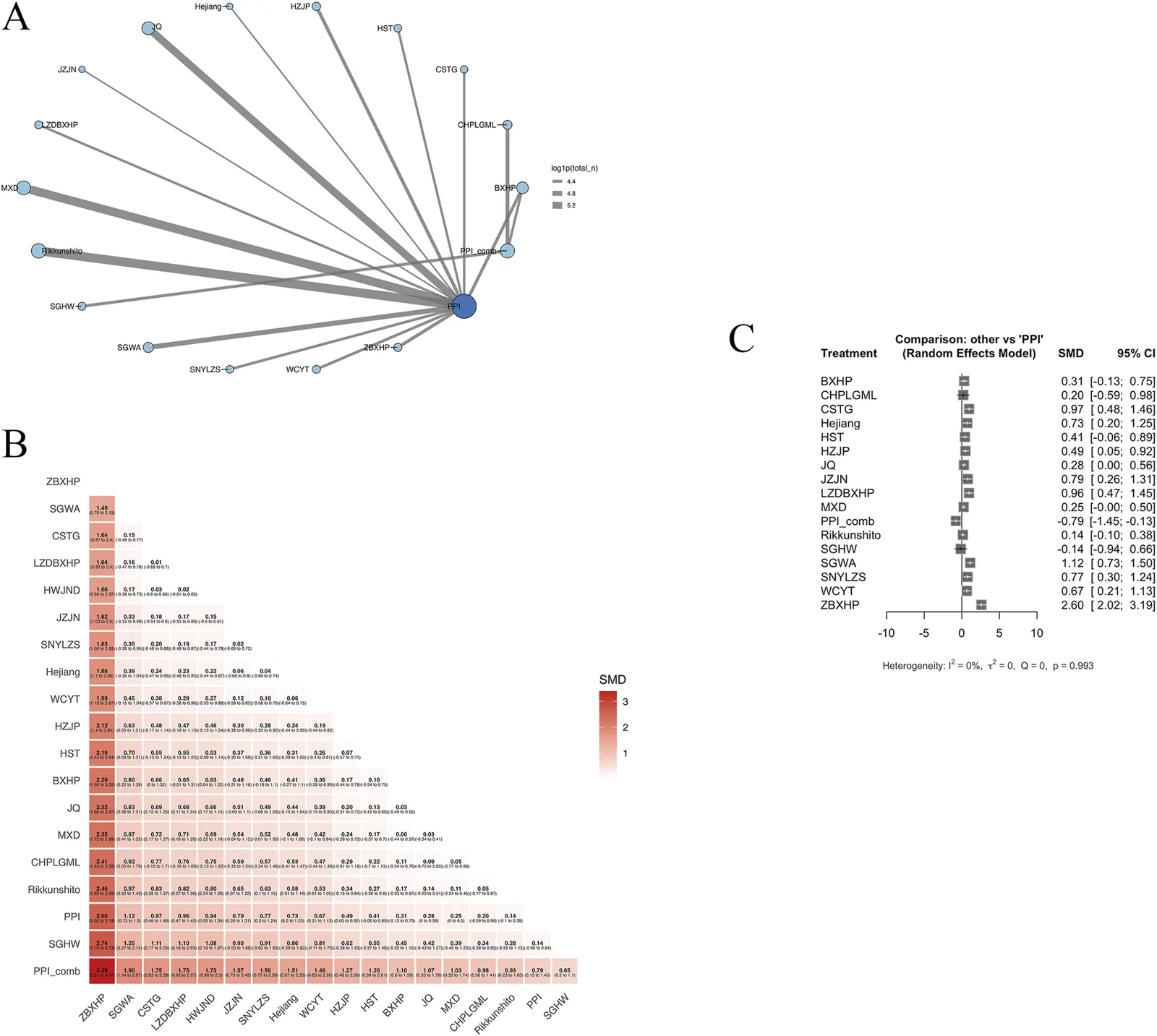
**(A)** Network geometry of the included interventions. Each node represents a treatment, with node size proportional to the number of participants and edge thickness indicating the number of direct comparisons. **(B)** League table of relative treatment effects. Values represent SMDs with corresponding 95% CIs. Cells above the diagonal indicate relative effects favoring column treatment over row treatment. The shading intensity reflects the magnitude of the effect, with darker colors representing stronger benefits. **(C)** Forest plot of network estimates for CHM versus PPI. Each point and horizontal bar represents the pooled network estimate (SMD [95% CI]) for one CHM relative to PPI as the reference treatment. Abbreviations: BXHP, Banxia Houpu Decoction; CHM, Chinese herbal medicine; CHPLGML, Chaihu Plus Longgu Muli Decoction; CI, confidence interval; CSTG, Chaishao Tongjian Prescription; HST, Hangeshashinto; HZJP, Hezhong Jianpi Decoction; JQ, JianpiQinghua Granules; JZJN, Jianzhong Jiangni Decoction; LZDBXHP, Modified Lizhong Decoction combined with Banxia Houpu Decoction; MXD, Modified Xiaochaihu Decoction; PPI, proton pump inhibitor; PPI_comb, PPI combined with mosapride; RKT, Rikkunshito; SGHW, Shugan Hewei Formula; SGWA, Shugan Weian Recipe; SMD, standard mean difference; SNYLZS, Sinivulianzheshi Decoction; WCYT, Wu Chu Yu Tang; ZBXHP, Zhang’s Banxia Houpu Decoction.

As shown in the league table (Figure 4B), several CHM prescriptions demonstrated superior efficacy compared with that of PPI, with the largest improvement observed for Zhang’s Banxia Houpu Decoction (ZBXHP; SMD = 2.60 [2.02, 3.19]) (Zhang Hui-Kai and Zhang, 2021), followed by Shugan Weian (SGWA) (Wang Guangming et al., 2020) and Chaishao Tongjian (CSTG) (Wang Jie, 2023).

The forest plot (Figure 4C) illustrates the overall effects of CHM versus PPI. No significant global inconsistency was detected using the design-by-treatment interaction model, and overall network heterogeneity was negligible (*I* ^*2*^ = 0%, τ^2^ = 0, *p* = 0.993), supporting the robustness and coherence of the network estimates.

### 3.10 Ranking of interventions

Ranking analysis using P-scores indicated that ZBXHP, SGWA, and CSTG were among the highest-ranked interventions for improving GERD symptoms (Figure 5). ZBXHP (Zhang Hui-Kai and Zhang, 2021) had the highest P-score; however, this ranking should be interpreted with caution, as the corresponding effect estimate was strongly influenced by a single trial with a large effect estimate.

**FIGURE 5:**
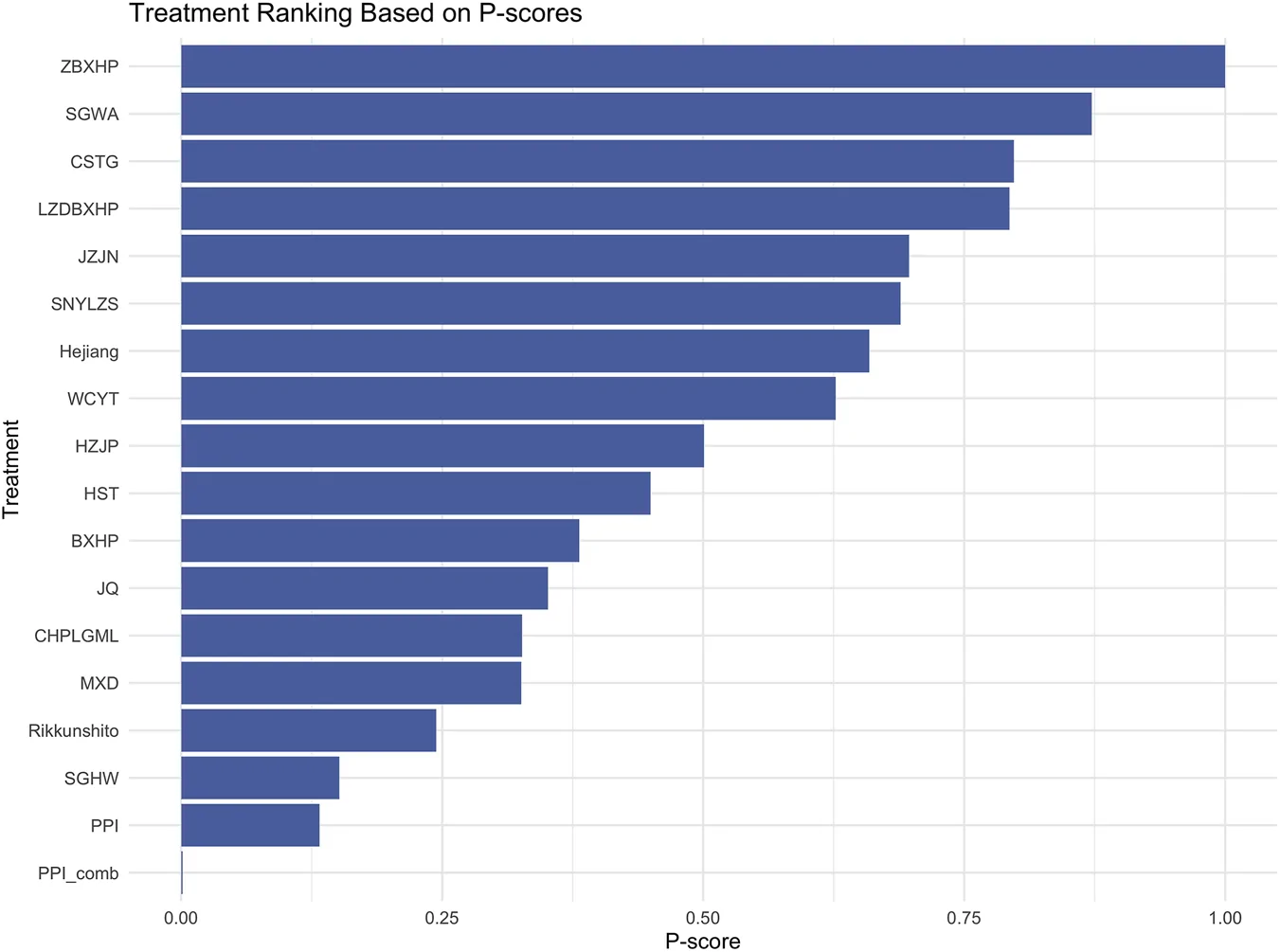
Treatment ranking based on P-scores. Abbreviations are the same as those described in the legend of Figure 4.

PPI, Modified Xiaochaihu Decoction (MXD) (Li Z. et al., 2021), and JianpiQinghua granules (JQ) (Zhang et al., 2021) were positioned toward the lower end of the ranking distribution. Given that P-scores provide a relative hierarchy within the analyzed network and do not by themselves establish clinically meaningful differences between treatments, treatment rankings were interpreted alongside the corresponding comparative effect estimates and confidence intervals. Detailed ranking plots for all treatments are presented in Supplementary Figures S7 and S8.

### 3.11 Sensitivity analysis

A sensitivity analysis was subsequently performed to examine the influence of the potential outlier (Zhang Hui-Kai and Zhang, 2021) on overall heterogeneity in the pairwise meta-analysis comparing CHM to PPI. After excluding this study, heterogeneity decreased from I^2^ = 81%–65%, while the pooled effect size remained statistically significant (SMD = 0.61 [0.44, 0.77]; p < 0.00001; Supplementary Figure S9). The large effect estimate observed in this study may also have contributed to the exceptionally high P-score of ZBXHP in the network analysis.

### 3.12 Transitivity and consistency

Statistical assessment of consistency was limited by the sparse network structure. In the design-by-treatment interaction model, the between-design component had zero degrees of freedom (df = 0), and therefore inconsistency could not be formally assessed using this approach. Node-splitting was also performed; however, no treatment comparison allowed separate estimation of direct and indirect effects. For within-design heterogeneity, no significant heterogeneity was found for PPI versus Rikkunshito (Q = 0.00, df = 1, p = 0.9928). Given these structural limitations, the transitivity assumption was further assessed qualitatively by comparing potential effect modifiers across treatment comparisons. Although some variation was observed in age, baseline symptom severity, and outcome measures, no major systematic imbalance in baseline patient and trial characteristics was identified across treatment comparisons, supporting the plausibility of the transitivity assumption (Supplementary Table S4).

### 3.13 Component contribution analysis

Due to the limited connectivity of the treatment network, a weighted meta-regression-based additive model derived from the frequentist CNMA framework (Rücker et al., 2020) was used to estimate the directional effects (β) of individual botanical drugs that appeared in more than three formulas.

As shown in Figure 6, component-level coefficient estimates varied across botanical drugs. *Citri reticulatae pericarpium* (chén pí), *Evodiae fructus* (wú zhū yú), *Cyperi rhizoma* (xiāng fù), *Magnoliae officinalis cortex* (hòu pò), *Atractylodis macrocephalae rhizoma* (bái zhú), *Pinelliae rhizoma* (bàn xià), *Coptidis rhizoma* (huáng lián), *Codonopsis radix* (dǎng shēn), and *Inulae flos* (xuán fù huā) showed positive or near-zero estimates (β ≥ 0). In contrast, *Aurantii fructus* (zhǐ shí), *Poria* (fú líng), *Bupleuri radix* (chái hú), *Glycyrrhizae radix et rhizoma* (gān cǎo), and *Zingiberis rhizoma* (jiāng) showed negative estimates (β < 0). A weighted bubble plot (Supplementary Figure S10) illustrates these component-level estimates, with bubble size representing precision (1/SE) and the x-axis denoting frequency of use.

**FIGURE 6:**
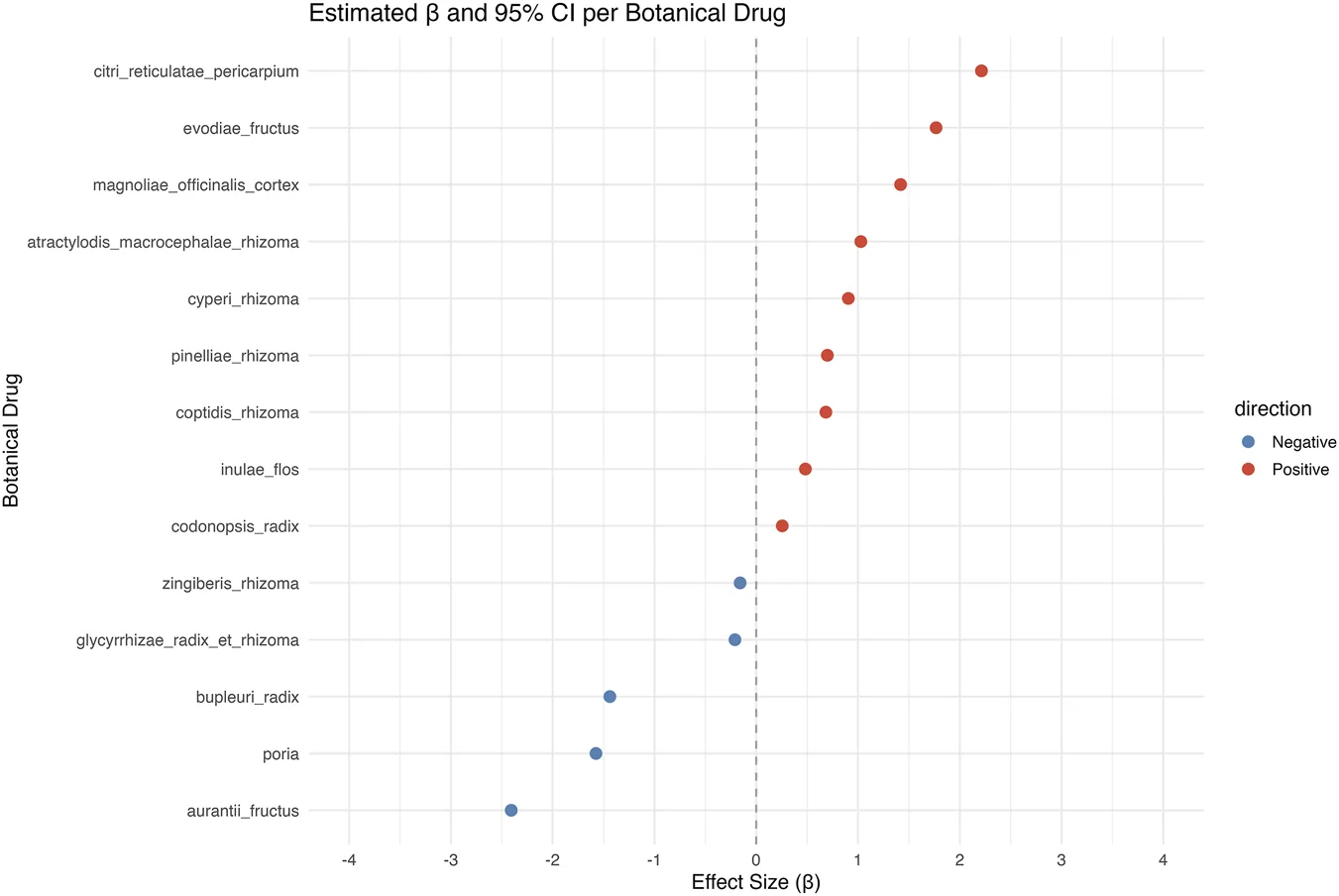
Component-level coefficient estimates for individual botanical drugs. The forest plot displays the estimated regression coefficients (β) and their 95% confidence intervals for each individual botanical drug, with a frequency of use greater than three (frequency >3), derived from the component contribution analysis. Components with β > 0 indicate a positive association with symptom improvement.

The core pattern network analysis identified 54 botanical drugs and 464 botanical drug pairs across all included trials (Figure 7). The six most frequently prescribed single botanical drug were *P. rhizoma* (bàn xià, 62.5%), *Coptidis rhizoma* (huáng lián, 50%), *Z. rhizoma* (jiāng, 50%), *Bupleuri radix* (chái hú, 43.75%), *G. radix et rhizoma* (gān cǎo, 43.75%), and *Evodiae fructus* (wú zhū yú, 43.75%).

**FIGURE 7:**
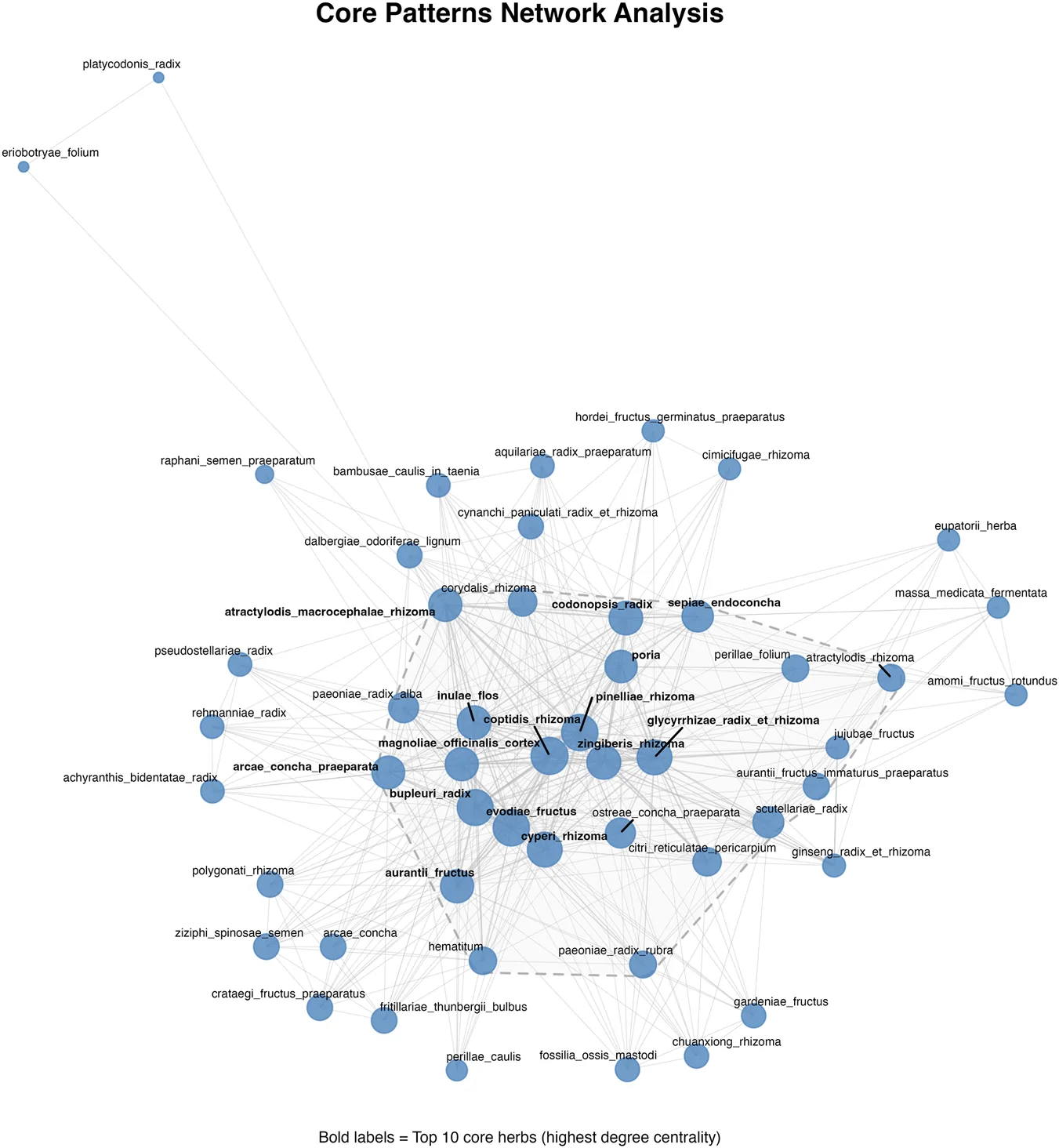
Core-pattern network analysis of botanical drug co-prescriptions across all included trials. Node size reflects the number of connections, and edge width represents the frequency of co-occurrence between botanical drugs.

In network visualization, node size and color intensity reflect the relative number of connections of each botanical drug within the network, whereas edge width indicates the frequency of co-occurrence between two botanical drugs. Within this structure, *P. rhizoma* (bàn xià) and *Coptidis rhizoma* (huáng lián) constituted the central nucleus of the prescription network, showing extensive connectivity with other components across the major formula clusters. The most frequently co-prescribed pairs were *Bupleuri radix–Coptidis rhizoma*, *Z. rhizoma–P. rhizoma*, *G. radix et rhizoma–P. rhizoma*, *Poria–P. rhizoma*, and *Evodiae fructus–Bupleuri radix* (n = 6 each). These recurrent co-prescription patterns indicate that frequently used botanical drugs were substantially interconnected within the analyzed prescription network (Supplementary Table S5).

### 3.14 CINeMa assessment

The CINeMA assessment revealed variability in confidence levels across the network (Supplementary Table S6). Among all treatment comparisons, 32.7% were rated as moderate confidence, 28.8% as low, and 38% as very low.

The primary factors contributing to downgrading were publication bias and the lack of blinding in several included trials, whereas other domains had minimal influence on the overall ratings. Comparisons involving JQ (Zhang et al., 2021), Wu Chu Yu Tang (WCYT) (Shih et al., 2019), and Shugan Hewei (SGHW) (Zhang Nan et al., 2024) versus PPI were generally rated as moderate confidence, indicating robust and coherent evidence. In contrast, comparisons based on smaller sample sizes or single-trial interventions, such as Hejiang Decoction (Lai Yaoliang, 2019), Jianzhong Jiangni (JZJN) (Zhou Yan et al., 2022), and Chaishao Tongjian (CSTG) (Wang Jie, 2023), were more frequently rated as low or very low confidence, largely due to these methodological limitations.

Overall, the CINeMA evaluation supports moderate confidence in the key CHM–PPI comparisons, reinforcing the robustness of the primary NMA findings.

## 4 Discussion

Gastroesophageal reflux disease remains a common and recurrent condition, with notable limitations in the long-term use of proton pump inhibitors. Although Chinese herbal medicines have shown clinical benefits, the contribution of individual botanical drugs has yet to be systematically explored.

Our meta-analysis of 20 RCTs involving 2,246 patients showed that CHM interventions were superior to control treatments in improving clinical efficacy and GERD symptom scores, with lower recurrence rates and without major safety concerns. NMA and component-level contribution analysis consistently supported these findings and revealed key formulas and botanical drugs contributing to overall efficacy.

Previous systematic reviews and meta-analyses have reported that CHM is effective for managing GERD and NERD (Xiao et al., 2018; Mu et al., 2022; Zhang et al., 2025). Xiao J et al. (Xiao et al., 2018) analyzed 14 RCTs involving 1,444 patients and reported that TCM achieved higher overall efficacy, lower recurrence rates, and fewer adverse events compared with conventional Western therapy in NERD patients. Mu J et al. (Mu et al., 2022) included 11 RCTs with 873 participants and found that Xiaoxiangxiong Decoction produced a significantly greater total effective rate than control interventions. Zhang R et al. (Zhang et al., 2025) synthesized 19 RCTs encompassing 1,585 patients and demonstrated that CHM combined with biomedicine provided significant adjunctive benefits in the treatment of refractory GERD. However, these studies primarily evaluated efficacy at the formula level, focusing on comparisons among complete CHM prescriptions rather than dissecting the contributions of individual botanical drugs. Our study extends this evidence base by integrating a frequentist NMA with a component-level contribution analysis grounded in the CNMA framework, complemented by core-pattern network analysis. This multi-layered approach not only compares the relative efficacy of CHM formulas but also elucidates the pharmacological contribution and interrelationships of individual botanical drug underlying therapeutic effectiveness.

Both pairwise and network meta-analyses consistently showed that CHM provided greater symptom relief and overall efficacy than conventional therapy. This consistency supports the robustness of our findings and suggests that the therapeutic benefits of CHM are not confined to a single prescription. The network results further identified ZBXHP, SGWA, and CSTG as the most effective formulae. In Chinese medicine, GERD is often related to liver and stomach constraint-heat or disharmony between the liver and stomach, where stagnated liver qi disrupts the descending function of the stomach (Ho et al., 2016). Banxia Houpu Decoction (BXHP) and its related preparations (Zhang Hui-Kai and Zhang, 2021; Song et al., 2024; Yu Danchun et al., 2017; Jia Ziliang et al., 2023; Wang Guangming et al., 2020; Wang Jie, 2023; Zhang Nan et al., 2024), formulated to disperse liver qi and regulate the stomach (shu gan he wei), correspond to this mechanism and may explain the favorable efficacy observed in network analysis. At the component level, further analyses revealed distinct contributions of individual botanical drugs, offering mechanistic insights that extend beyond formula-level comparisons.

The botanical drug frequency analysis showed that *P. rhizoma* (bàn xià, 62.5%), *Coptidis rhizoma* (huáng lián, 50%), *Z. rhizoma* (jiāng, 50%) were the most frequently prescribed botanical drugs, followed by *Bupleuri radix* (chái hú, 43.75%), *G. radix et rhizom*a (gān cǎo, 43.75%), and *Evodiae fructus* (wú zhū yú, 43.75%).

At the component level, individual botanical drugs showed distinct coefficient estimates; however, these estimates should be interpreted within the composition of the overall prescription network rather than as independent effects of individual botanical drugs. Among the botanical drugs with positive estimates, *C. reticulatae pericarpium* (chén pí) showed the largest positive coefficient in the component analysis. Together with *Cyperi rhizoma* (xiāng fù), which also showed a relatively large positive estimate, these botanical drugs are traditionally used to regulate qi and relieve gastrointestinal discomfort. Xiāng fù contains volatile oils and flavonoids that regulate smooth muscle contractions and alleviate visceral hypersensitivity (Lei et al., 2025), while *C. reticulatae pericarpium*, containing active metabolites such as hesperidin, nobiletin, and tangeretin, has been reported to exert prokinetic effects by accelerating gastric emptying, enhancing intestinal transit, and modulating gastrointestinal peptides (Hu et al., 2025). In addition, network pharmacology analysis identified several flavonoids, including hesperidin, as core bioactive metabolites associated with its effects against spleen deficiency, further supporting its traditional role in regulating digestive function (Zheng et al., 2020). These gastrointestinal regulatory actions may help interpret the positive component-level estimates observed in the analysis. *Evodiae fructus* (wú zhū yú) and *Coptidis rhizoma* (huáng lián) also showed positive estimates in the component analysis. *Evodiae fructus*, a major botanical drug of WCYT, demonstrates antibacterial activity against *Helicobacter pylori* and ameliorates gastric and duodenal ulceration. In addition, rutaecarpine, a principal metabolite of *Evodiae fructus*, inhibits cyclooxygenase-2 (Na et al., 2022). *Coptidis rhizoma* (huáng lián) contains alkaloids, including berberine, coptisine, palmatine, and jatrorrhizine. Specifically, berberine protects against esophageal mucosal damage in reflux-induced esophagitis by suppressing proinflammatory signaling such as NF-κB (Kwon et al., 2016). Notably, a recent study demonstrates that berberine induces concentration-dependent relaxation of the LES via potassium-channel activation, suggesting that its pharmacological actions relevant to GERD may be multifaceted. Nevertheless, *Coptidis rhizoma* exhibits anti-inflammatory, antibacterial, and cytoprotective effects on other key pathological domains of GERD (Koh et al., 2024). In traditional practice, huáng lián and wú zhū yú are paired to harmonize cold–heat properties within formulas, such as Zuo Jin Wan. Their co-occurrence within the analyzed prescriptions may reflect established prescription patterns and potentially complementary pharmacological properties (Wei et al., 2022). *Codonopsis radix* (dǎng shēn) is traditionally used to strengthen the spleen and replenish qi. Pharmacological studies have indicated that it promotes gastric motility, protects the mucosa, and modulates immune and antioxidant pathways. Mechanistically, *C. radix* and several of its anti-inflammatory metabolites, such as luteolin, curcumin, and stigmasterol, regulate the phosphatidylinositol-3-kinase/protein kinase B, tumor necrosis factor (TNF), and nuclear factor kappa B (NF-κB) signaling pathways, thereby contributing to inflammation control and mucosal healing (He et al., 2021). *Magnoliae officinalis cortex* (hòu pò) is traditionally used to relieve abdominal fullness and modern pharmacological studies have supported its ability to enhance gastric emptying and promote digestion. These prokinetic actions are attributed in part to magnolol and honokiol, which accelerate gastric emptying and intestinal propulsion and may also involve modulation of the c-kit/stem cell factor signaling pathway. Both metabolites also suppress the activation of mitogen-activated protein kinase and NF-κB, thereby providing gastrointestinal protection and anti-inflammatory activity (Luo et al., 2019). *Pinelliae rhizoma* (bàn xià), the most frequently used botanical drug, plays a central role in relieving nausea and regulating gastric motility. It is widely used for the treatment of emesis and gastric ulcers. Its antiemetic activity is primarily attributed to Pinellia alkaloids, which inhibit isolated ileum contraction tension and function as antagonists of 5-HT3 and neurokinin-1 receptors (Zou et al., 2023). Its frequent co-occurrence with multiple botanical drugs also suggests that its individual association may be difficult to disentangle from the broader prescription patterns.

In contrast, negative component-level estimates were observed for *Aurantii fructus* (zhǐ shí), *Poria* (fú líng), and *Bupleuri radix* (chái hú), whereas the estimate for *G. radix et rhizoma* (gān cǎo) and *Z. rhizoma* (jiāng) were slightly negative but close to the null. *Glycyrrhizae radix et rhizoma* (gān cǎo) exhibits potent anti-ulcer and mucosal protective effects through its bioactive metabolites, including flavonoids such as isoliquiritigenin, liquiritigenin, as well as carbon-dot derivatives. These metabolites suppress proinflammatory cytokine production, oxidative stress, nitric oxide production, and the expression of inducible nitric oxide synthase, while enhancing mucus secretion and epithelial repair, thereby strengthening gastric mucosal defense (Liu et al., 2021; Tanemoto et al., 2015). In CHM prescriptions, *G. radix et rhizoma* is also frequently used as an adjuvant or harmonizing botanical drug to coordinate the actions of other ingredients rather than serving as the principal therapeutic agent. This prescription role may provide additional context for its near-null estimate in the component analysis. *Zingiberis rhizoma* (jiāng) exerts dual antiemetic and gastroprotective effects. It neutralizes gastric acid and reduces acid-induced mucosal irritation, whereas its major metabolites, 6-gingerol and 6-shogaol, stimulate digestive juice secretion. In addition, 6-gingerol suppresses the 5-hydroxytryptamine type 3 receptor (5-HT_3_R)/Ca^2+^ pathway, contributing to its antiemetic effect. Importantly, by modulating smooth-muscle contraction through muscarinic type 3 receptors (M_3_Rs) and 5-HT_3_Rs, standardized 6-gingerol extracts increase lower esophageal sphincter (LES) tone and reduce reflux events (Li X. et al., 2021; Gu et al., 2025; Promdam et al., 2022). *Bupleuri radix* (chái hú) exhibits immunomodulatory and anti-inflammatory activities. Saikosaponin, a bioactive triterpenoid glycoside (saponin) metabolite derived from *Bupleuri radix*, suppresses NF-κB nuclear translocation via the extracellular signal-regulated kinase pathway, leading to reduced expression of TNF-α, IL-1β, and IL-6, and decreased production of nitric oxide (Kim et al., 2015). *Poria* (fú líng) has also been reported to exert gastrointestinal regulatory effects. *Poria* polysaccharides may enhance digestive function and accelerate gastrointestinal motility, thereby alleviating dyspeptic symptoms (Wang et al., 2022). These apparent discrepancies illustrate that the direction of a component coefficient does not necessarily correspond to the known pharmacological properties of the individual botanical drug. The botanical drug-pair analysis further informs the interpretation of these component-level estimates. Several frequently represented botanical drugs repeatedly co-occurred within the same formulations, indicating substantial structural dependence among components within the prescription network. In the additive component model, such recurrent co-prescription may make the contribution of individual components difficult to disentangle, particularly when specific botanical drugs are preferentially prescribed together. A mechanistic overview of the frequently used CHM in GERD management is shown in Figure 8.

**FIGURE 8:**
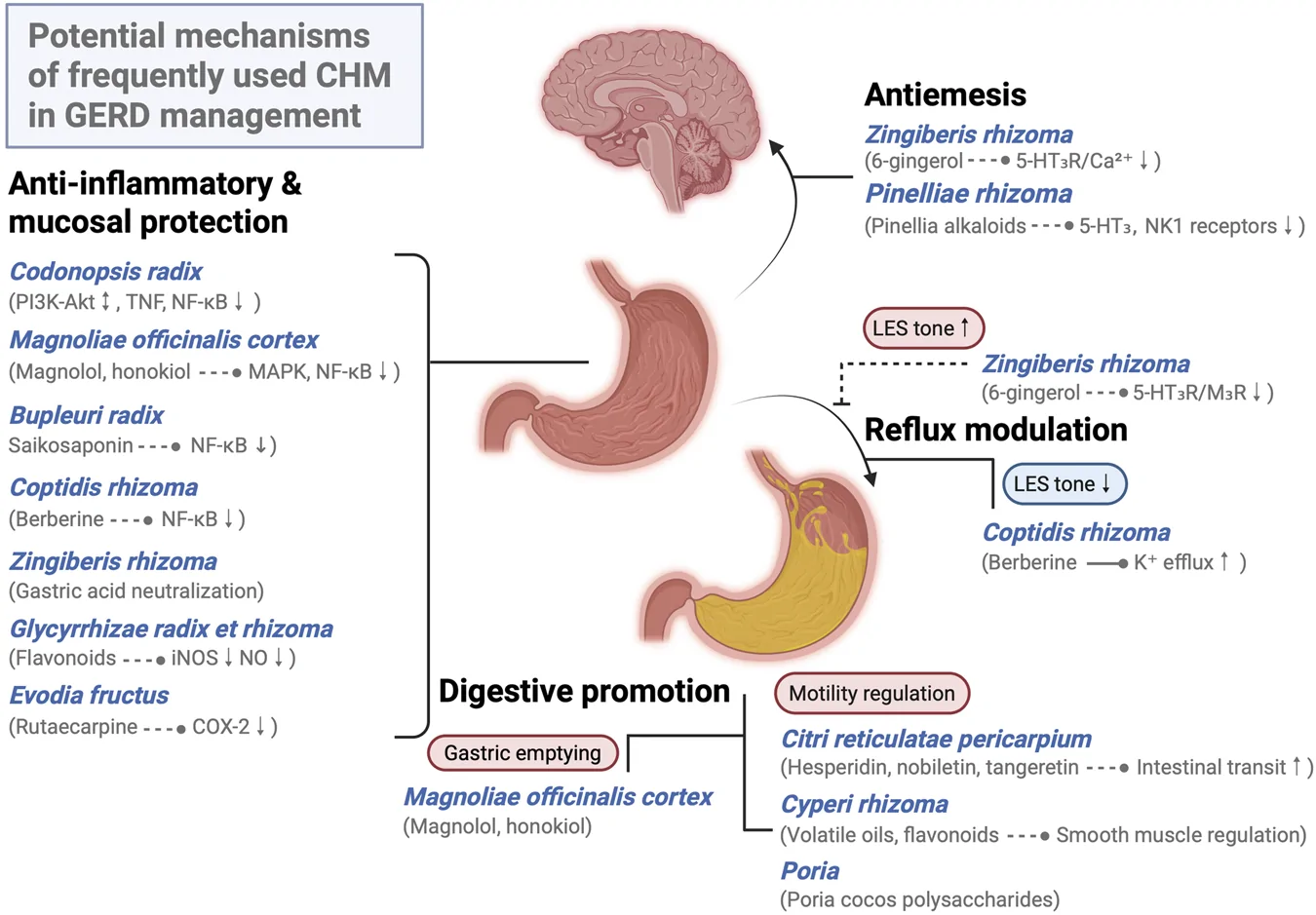
Potential mechanisms of frequently used CHM in GERD management. Frequently used CHM may act through four major mechanistic domains: (1) anti-inflammatory and mucosal protection via suppression of NF-κB/MAPK pathways and inhibition of COX-2 and iNOS; (2) antiemesis through 6-gingerol–mediated inhibition of 5-HT_3_Rs/Ca^2+^ influx and Pinellia alkaloid-induced inhibition of 5-HT_3_Rs and NK1 receptors; (3) reflux modulation via alteration of LES tone through 5-HT_3_R/M_3_R pathways (Z*ingiberis rhizoma*) or increased K^+^ efflux (*Coptidis rhizoma*); and (4) digestive promotion through enhanced gastric emptying (*Magnoliae officinalis cortex*) and motility regulation involving enhanced intestinal transit (*Citri reticulatae pericarpium*) and smooth muscle regulation (*Cyperi rhizoma*). Abbreviations: 5-HT_3_R, 5-hydroxytryptamine type 3 receptor; CHM, Chinese herbal medicine; COX-2, cyclooxygenase-2; GERD, gastroesophageal reflux disease; IL, interleukin; iNOS, inducible nitric oxide synthase; LES, lower esophageal sphincter; M_3_R, muscarinic type 3 receptor; MAPK, mitogen-activated protein kinase; NF-κB, nuclear factor kappa B; NK1, neurokinin-1 receptor; TNF-α, tumor necrosis factor-alpha. Created in BioRender. YiTing, C. (2026) https://BioRender.com/43plep4.

In recent years, concerns have grown regarding the increasing number of patients on long-term PPI therapy, the widespread inappropriate use of PPIs, and associated safety considerations (Helgadottir and Bjornsson, 2019). Potential botanical drug–drug interactions should also be considered when CHM is used in combination with PPIs, as CHM products may modulate drug-metabolizing enzymes and transporters, including CYP2C19 and CYP3A4, thereby altering the systemic exposure of co-administered drugs. For example, red ginseng has demonstrated potential interactions with several drug-metabolizing enzymes and transporters, although the clinical relevance may vary according to the composition and preparation of herbal products (Kwon et al., 2020). Beyond pharmacokinetic interactions, hepatic safety warrants attention during combined therapy because both PPI-associated drug-induced liver injury (DILI) and CHM-associated herb-induced liver injury (HILI) have been reported. Although PPI-associated DILI is uncommon, several PPIs, including omeprazole and esomeprazole, have been implicated in published cases of idiosyncratic DILI (Björnsson and Hoofnagle, 2016). Based on the updated Roussel Uclaf Causality Assessment Method (RUCAM), omeprazole was identified as a major causative gastrointestinal medication associated with DILI in children (11%) (Ye et al., 2021). Various CHMs have also been implicated in HILI, with numerous cases assessed for causality using RUCAM. When hepatic adverse events occur during combined CHM–PPI treatment, causality cannot be assigned to either component solely on the basis of temporal association. RUCAM provides a structured, quantitative approach for assessing both DILI and HILI, requiring individual assessment of each co-administered product while considering concomitant agents and alternative etiologies (Danan and Teschke, 2015; Teschke and Danan, 2021). In our analysis, CHM therapy was associated with lower rates of adverse events and recurrence, with no evidence of treatment-related toxicity or organ impairment. These findings suggest that CHM may serve as a safer and more sustainable alternative or complementary approach to long-term PPI use, potentially helping to reduce dependence on acid-suppressive therapy while maintaining symptom control and mucosal protection.

This study has some limitations. First, considerable heterogeneity among studies may have arisen from variations in CHM formula composition, dosage, and preparation methods, reflecting the clinical diversity of individualized prescriptions. The markedly large effect estimate reported in the study evaluating ZBXHP also contributed to heterogeneity and may have influenced its high P-score, warranting caution in the interpretation of its treatment ranking. Second, the treatment network showed limited connectivity, with no closed loop and sparse indirect comparisons, reducing the precision of the NMA estimates, direct and indirect evidence could not be separately compared using node-splitting, while the between-design component of the design-by-treatment interaction model had zero degrees of freedom. Such limitations in assessing inconsistency have been recognized in methodological studies of network meta-analysis (Seitidis et al., 2023). Third, the lack of a double-blind design in several RCTs contributed to downgrading the CINeMA assessment and may have introduced performance bias. These limitations should be considered when interpreting the results, and future large-scale, double-blinded RCTs with standardized protocols are warranted to enhance evidence certainty. Finally, the availability and economic accessibility of CHMs vary substantially across healthcare systems. In several East Asian countries, traditional medicine is more extensively integrated into national healthcare systems, which may facilitate access and affordability (World Health Organization, 2013). In contrast, access to CHMs in Western countries is influenced by differences in regulatory frameworks and healthcare coverage. Cross-national analyses have demonstrated considerable variation in the regulation and availability of Chinese herbal products, and only a limited number of CHM-based herbal medicinal products have obtained formal registration in Europe. Consequently, the availability of the specific CHM formulations evaluated in the present study cannot be assumed to be equivalent across countries. Treatment costs may likewise vary according to healthcare and insurance coverage (World Health Organization, 2013; Fleischer et al., 2017; Bilia et al., 2025). As comparative economic data were not reported in the included RCTs, the relative cost or cost-effectiveness of CHM compared with PPI therapy could not be determined.

Based on the current evidence, CHM may be considered as a potential treatment option for patients with GERD. However, patients should use CHM under the prescription and supervision of qualified healthcare professionals rather than selecting CHM formulations according to treatment rankings. Patients should inform their healthcare providers of all concomitant medications, particularly PPIs and other gastrointestinal medications, to facilitate the assessment of potential botanical drug–drug interactions. Possible adverse events should also be monitored during treatment, and patients should seek medical evaluation if clinically relevant adverse symptoms occur. Given the varying certainty of evidence across individual CHM formulations, treatment selection and continuation should be individualized according to the patient’s clinical condition, current therapy, treatment response, and available evidence.

## 5 Conclusion

To the best of our knowledge, this study represents the first application of component-level contribution analysis to evaluate the contribution structure of complex CHM prescriptions for GERD. Our findings suggest that CHM may provide clinical benefits compared with conventional therapies, with improvements in clinical efficacy and recurrence control while maintaining a comparable safety profile. The integration of network and component-level analyses provides insights into prescription structures, frequently co-prescribed botanical drugs, and component-level associations, providing a quantitative framework for linking CHM prescription patterns with evidence-based pharmacology. These findings underscore the potential of CHM to strengthen the esophagogastric barrier and reduce long-term dependence on PPIs. Nevertheless, component-level estimates should not be interpreted as independent therapeutic effects of individual botanical drugs. Further rigorously designed, large-scale clinical trials are required to validate these effects, clarify their clinical relevance, delineate the underlying mechanisms, and refine multi-target CHM strategies in GERD management.

## Data Availability

The original contributions presented in the study are included in the article/Supplementary Material, further inquiries can be directed to the corresponding author.

## Glossary

Gastroesophageal Reflux Disease (GERD): A chronic gastrointestinal disorder characterized by the reflux of gastric contents into the esophagus, causing symptoms such as heartburn and regurgitation and, in some cases, esophageal mucosal injury.
Nonerosive Reflux Disease (NERD): A subtype of GERD in which patients experience typical reflux symptoms without visible esophageal mucosal damage on endoscopy.
Proton Pump Inhibitors (PPIs): A class of acid-suppressive drugs that irreversibly inhibit the gastric hydrogen–potassium ATPase (proton pump), thereby reducing gastric acid secretion.
Chinese Herbal Medicine (CHM): A therapeutic system within traditional Chinese medicine that uses multi-herb formulations designed according to TCM theory to restore physiological balance and treat disease.
Traditional Chinese Medicine (TCM): A holistic medical system originating in China, based on concepts such as Yin–Yang balance, qi, and organ-system harmony, and including herbal medicine, acupuncture, and dietary therapy.
Lower Esophageal Sphincter (LES): A ring of smooth muscle at the junction of the esophagus and stomach that prevents reflux of gastric contents.
**5-Hydroxytryptamine Type 3 Receptor (5-HT**_**3**_ **R)**: A serotonin receptor involved in gastrointestinal motility and emesis, commonly targeted in antiemetic therapy.
