# Supplementary material for: Treatment effects and safety of Chinese herbal medicine in gastroesophageal reflux disease: a systematic review of randomized controlled trials and a network meta-analysis with component analysis
Source: Front Pharmacol. 2026 Sep 24;17:1920738. doi: 10.3389/fphar.2026.1920738 (PMC13646106; doi:10.3389/fphar.2026.1920738)
Supplement: Supplementary file 4 [file Table2.docx]

**# Network meta-analysis and treatment ranking**

nma <- netmeta::netmeta(

TE = TE,

seTE = seTE,

treat1 = treat1,

treat2 = treat2,

studlab = study,

data = effect_data,

sm = "SMD",

reference.group = "PPI",

random = TRUE,

common = FALSE,

method.tau = "REML",

hakn = FALSE,

prediction = FALSE,

favour.higher = "better"

)

ranking_results <- netmeta::netrank(

nma,

small.values = "bad"

)

rank_table <- data.frame(

Treatment = names(ranking_results$Pscore.random),

Pscore = as.numeric(ranking_results$Pscore.random),

stringsAsFactors = FALSE

)

rank_table <- rank_table[

order(rank_table$Pscore, decreasing = TRUE),

]

**# Consistency assessment**

global_consistency <- netmeta::decomp.design(nma)

netsplit_result <- netmeta::netsplit(

nma,

common = FALSE,

random = TRUE,

details = TRUE,

show = "both"

)

df <- netsplit_result$random

colnames(df) <- make.unique(colnames(df))

consistency_table <- df |>

tibble::as_tibble(rownames = "comparison")

**# Component contribution analysis**

effect_by_treat <- effect_data |>

dplyr::group_by(treat1) |>

dplyr::summarise(

TE = sum(TE / seTE^2) / sum(1 / seTE^2),

seTE = sqrt(1 / sum(1 / seTE^2)),

.groups = "drop"

)

component_data <- component_matrix |>

dplyr::filter(treat1 %in% effect_by_treat$treat1) |>

dplyr::arrange(match(treat1, effect_by_treat$treat1))

effect_by_treat <- effect_by_treat |>

dplyr::filter(treat1 %in% component_data$treat1) |>

dplyr::arrange(match(treat1, component_data$treat1))

X <- component_data |>

dplyr::select(-treat1) |>

dplyr::mutate(

dplyr::across(

dplyr::everything(),

as.numeric

)

)

freq <- colSums(X)

X <- X[, freq > 3, drop = FALSE]

fit <- stats::lm(

effect_by_treat$TE ~ .,

data = X,

weights = 1 / effect_by_treat$seTE^2

)

coef_table <- summary(fit)$coefficients[-1, , drop = FALSE]

crit <- stats::qt(0.975, df = stats::df.residual(fit))

component_results <- data.frame(

herb = rownames(coef_table),

estimate = coef_table[, "Estimate"],

se = coef_table[, "Std. Error"]

) |>

dplyr::mutate(

ci_low = estimate - crit * se,

ci_high = estimate + crit * se,

direction = ifelse(

estimate > 0,

"Positive",

"Negative"

)

)

**# Herb frequency and co-occurrence analysis**

component_matrix_core <- component_matrix |>

dplyr::distinct(

treat1,

.keep_all = TRUE

) |>

dplyr::mutate(

dplyr::across(

-treat1,

~ ifelse(is.na(.), 0, .)

)

)

mat <- as.matrix(

component_matrix_core[, -1]

)

rownames(mat) <- component_matrix_core$treat1

co_matrix <- t(mat) %*% mat

freq_df <- data.frame(

herb = colnames(mat),

frequency = colSums(mat),

freq_pct = colSums(mat) /

nrow(mat) * 100

)

pairs_list <- which(

co_matrix > 0,

arr.ind = TRUE

)

pair_df <- data.frame(

herb1 = rownames(co_matrix)[pairs_list[, 1]],

herb2 = colnames(co_matrix)[pairs_list[, 2]],

co_occurrence = co_matrix[pairs_list]

)

pair_df <- pair_df |>

dplyr::filter(herb1 != herb2) |>

dplyr::mutate(

pair_id = paste(

pmin(herb1, herb2),

pmax(herb1, herb2),

sep = "_"

)

) |>

dplyr::distinct(

pair_id,

.keep_all = TRUE

) |>

dplyr::arrange(

dplyr::desc(co_occurrence)

)
