# Supplementary material for: Treatment effects and safety of Chinese herbal medicine in gastroesophageal reflux disease: a systematic review of randomized controlled trials and a network meta-analysis with component analysis
Source: Front Pharmacol. 2026 Sep 24;17:1920738. doi: 10.3389/fphar.2026.1920738 (PMC13646106; doi:10.3389/fphar.2026.1920738)
Supplement: Supplementary file 5 [file Table3.docx]

**Supplementary Table S1. Details of the search strategy in MEDLINE (Ovid) and PubMed.**

| Database | # | Search syntax | Citations found |
| --- | --- | --- | --- |
| MEDLINE (Ovid) | 1 | (GERD OR GORD OR (Reflux* ADJ4 (Gastr* OR Esophag* OR Oesophag* OR Acid*))).mp | 49687 |
|  | 2 | "Esophageal Motility Disorders"/ OR exp "Gastroesophageal Reflux"/ | 33036 |
|  | 3 | ((Chinese ADJ4 medic*) OR CHM OR CAM OR herb* OR phytotherap* OR phytomedicine OR "chung i" OR "zhong yi" OR ((chinese OR orienta* OR kampo OR botanic* OR phyto* OR plant*) ADJ6 (drug* OR medic* OR formula* OR remed* OR preparation* OR capsule* OR pellet* OR solution* OR pill* OR powder* OR granule* OR granula* OR extract* OR product* OR agent* OR substance* OR material* OR traditional OR phyto* OR plant* OR therap* OR treatment* OR intervention* OR supplement*)) OR ((complementary OR alternative) ADJ6 (drug* OR medic* OR formula* OR remed* OR preparation* OR therap* OR treamment* OR intervention*)) OR fang OR tang OR wan OR san OR dan OR decoction*).mp | 1777665 |
|  | 4 | "Complementary Therapies"/ OR exp "Medicine, Traditional"/ OR exp "Phytotherapy"/ OR exp "Plant Preparations"/ OR exp "Herbal Medicine"/ OR exp "Plants"/ | 672842 |
|  | 7 | (1 OR 2) AND (3 OR 4) | 1027 |

| Pubmed | 1 | GERD[tw] OR GORD[tw] OR "Acid Reflux"[tiab:~3] OR "Gastroesophageal reflux"[tiab:~3] OR "Gastro-oesophageal reflux"[tiab:~3] OR "esophageal gastric reflux"[tiab:~3] | 34205 |
| --- | --- | --- | --- |
|  | 2 | "Esophageal Motility Disorders"[mh:noexp] OR "Gastroesophageal Reflux"[mh] | 33028 |
|  | 3 | Chinese medic*[tw] OR CHM[tw] OR CAM[tw] OR herb*[tw] OR phytotherap*[tw] OR phytomedicine[tw] OR "chung i"[tw] OR "zhong yi"[tw] OR (chinese[tw] OR orienta*[tw] OR kampo[tw] OR botanic*[tw] OR phyto*[tw] OR plant*[tw]) AND (drug*[tw] OR medic*[tw] OR formula*[tw] OR remed*[tw] OR preparation*[tw] OR capsule*[tw] OR pellet*[tw] OR solution*[tw] OR pill*[tw] OR powder*[tw] OR granule*[tw] OR granula*[tw] OR extract*[tw] OR product*[tw] OR agent*[tw] OR substance*[tw] OR material*[tw] OR traditional[tw] OR phyto*[tw] OR plant*[tw] OR therap*[tw] OR treatment*[tw] OR intervention*[tw] OR supplement*[tw]) OR ((complementary[tw] OR alternative[tw]) AND (drug*[tw] OR medic*[tw] OR formula*[tw] OR remed*[tw] OR preparation*[tw] OR therap*[tw] OR treamment*[tw] OR intervention*[tw])) OR fang[tw] OR tang[tw] OR wan[tw] OR san[tw] OR dan[tw] OR decoction*[tw] | 2296178 |
|  | 4 | "Complementary Therapies"[mh:noexp] OR "Medicine, Traditional"[mh] OR "Phytotherapy"[mh] OR "Plant Preparations"[mh] OR "Herbal Medicine" OR "Plants"[mh] | 686307 |
|  | 7 | (#1 OR #2) AND (#3 OR #4) | 1841 |
|  | 8 | #5 AND (randomized controlled trial[pt] OR controlled clinical trial[pt] OR randomized[tiab] OR randomised[tiab] OR placebo[tiab] OR drug therapy[sh] OR randomly[tiab] OR trial[tiab] OR groups[tiab] NOT (animals [mh] NOT humans [mh])) | 798 |

**Supplementary Table S2. Detailed information for ConPhyMP assessment**

| Study | Formula | Dosage form | Manufacturer/ Preparation source | Processing / Extraction | Modification | Quality information | |
| --- | --- | --- | --- | --- | --- | --- | --- |
| Cai ZZ et al., 2024 | Chaihu Plus Longgu Muli (CHPLGML) Decoction | Herbal  Decoction | Not reported | Prepared as a decoction (300 mL/day) | No modifications reported | Not reported | |
|  | Composition:   - Gardenia jasminoides J.Ellis [Rubiaceae; Gardeniae Fructus], 10 g - Citrus reticulata Blanco [Rutaceae; Citri Reticulatae Pericarpium], 10 g - Paeonia lactiflora Pall. or Paeonia veitchii Lynch [Paeoniaceae; Paeoniae Radix Rubra], 10 g - Conioselinum anthriscoides 'Chuanxiong' [Apiaceae; Chuanxiong Rhizoma], 10 g - Bupleurum chinense DC. or Bupleurum scorzonerifolium Willd. [Apiaceae; Bupleuri Radix], 9 g - Scutellaria baicalensis Georgi [Lamiaceae; Scutellariae Radix], 6 g - Fossil medicinal material [Not applicable; Fossilia Ossis Mastodi], 6 g - *Ostrea gigas* Thunberg, *Ostrea talienwhanensis* Crosse, or *Ostrea rivularis* Gould (animal-derived medicinal material) [Ostreidae; *Ostreae Concha*]Cyperus rotundus L. [Cyperaceae; Cyperi Rhizoma], 6 g - Pinellia ternata (Thunb.) Makino [Araceae; Pinelliae Rhizoma], 6 g - Glycyrrhiza glabra L., Glycyrrhiza uralensis Fisch. ex DC., or Glycyrrhiza inflata Batalin [Fabaceae; Glycyrrhizae Radix et Rhizoma], 6 g - Citrus × aurantium L. [Rutaceae; Aurantii Fructus], 6 g - Tetradium ruticarpum (A.Juss.) T.G.Hartley [Rutaceae; Evodiae Fructus], 3 g | | | | | |  |
| Jia ZL et al., 2023 | Modified Lizhong Decoction combined with Banxia Houpu (LZDBXHP) Decoction | Herbal  Decoction | Not reported | Prepared as a decoction (300 mL/day) | Symptom-based prescription modifications were reported. | Not reported | |
|  | Composition:   - Codonopsis pilosula (Franch.) Nannf. subsp. pilosula or Codonopsis pilosula subsp. tangshen (Oliv.) D.Y.Hong [Campanulaceae; Codonopsis Radix], 15 g - Atractylodes macrocephala Koidz. [Asteraceae; Atractylodis Macrocephalae Rhizoma], 20 g - Zingiber officinale Roscoe [Zingiberaceae; Zingiberis Rhizoma], 10 g - Pinellia ternata (Thunb.) Makino [Araceae; Pinelliae Rhizoma], 15 g - Wolfiporia cocos (F.A. Wolf) Ryvarden & Gilb. [Polyporaceae; Poria], 10 g - Magnolia officinalis Rehder & E.H.Wilson or Magnolia officinalis var. biloba Rehder & E.H.Wilson [Magnoliaceae; Magnoliae Officinalis Cortex], 10 g - Actaea heracleifolia (Kom.) J.Compton, Actaea dahurica (Turcz. ex Fisch. & C.A.Mey.) Franch., or Actaea cimicifugaL. [Ranunculaceae; Cimicifugae Rhizoma], 6 g - Sepiella maindroni de Rochebrune or Sepia esculenta Hoyle [Sepiidae; Sepiae Endoconcha], 20 g - Hordeum vulgare L. [Poaceae; Hordei Fructus Germinatus Praeparatus], 15 g - Glycyrrhiza glabra L., Glycyrrhiza uralensis Fisch. ex DC., or Glycyrrhiza inflata Batalin [Fabaceae; Glycyrrhizae Radix et Rhizoma Praeparata cum Melle], 10 g | | | | | |  |
| Lai YL et al., 2019 | Hejiang therapy | Granules | Beijing Kangrentang Pharmaceutical Co., Ltd. | Not reported. | Symptom-based prescription modifications were reported. | Not reported | |
|  | Composition:   - Bupleurum chinense DC. or Bupleurum scorzonerifolium Willd. [Apiaceae; Bupleuri Radix], 10 g - Paeonia lactiflora Pall. [Paeoniaceae; Paeoniae Radix Alba], 10 g - Citrus × aurantium L. [Rutaceae; Aurantii Fructus], 10 g - Pinellia ternata (Thunb.) Makino [Araceae; Pinelliae Rhizoma Praeparatum], 9 g - Cyperus rotundus L. [Cyperaceae; Cyperi Rhizoma], 10 g - Perilla frutescens (L.) Britton [Lamiaceae; Perillae Caulis], 10 g - Magnolia officinalis Rehder & E.H.Wilson or Magnolia officinalis var. biloba Rehder & E.H.Wilson [Magnoliaceae; Magnoliae Officinalis Cortex], 10 g - Coptis chinensis Franch., Coptis deltoidea C.Y.Cheng & P.K.Hsiao, or Coptis teeta Wall. [Ranunculaceae; Coptidis Rhizoma], 6 g - Tetradium ruticarpum (A.Juss.) T.G.Hartley [Rutaceae; Evodiae Fructus], 3 g | | | | | |  |
| Li Z et al., 2021 | Modified Xiaochaihu Decoction (MXD) | Granules | Jiangyin Tianjiang Pharmaceutical Co., Ltd. | Not reported | Mainly composed of the listed herbs; additional modifications were not reported. | Not reported | |
|  | Composition:   - Bupleurum chinense DC. or Bupleurum scorzonerifolium Willd. [Apiaceae; Bupleuri Radix], 10 g - Codonopsis pilosula (Franch.) Nannf. subsp. pilosula or Codonopsis pilosula subsp. tangshen (Oliv.) D.Y.Hong [Campanulaceae; Codonopsis Radix], 15 g - Atractylodes macrocephala Koidz. [Asteraceae; Atractylodis Macrocephalae Rhizoma Praeparatum], 12 g - Coptis chinensis Franch., Coptis deltoidea C.Y.Cheng & P.K.Hsiao, or Coptis teeta Wall. [Ranunculaceae; Coptidis Rhizoma], 10 g - Pentanema britannica (L.) D.Gut.Larr., Santos-Vicente, Anderb., E.Rico & M.M.Mart.Ort. or Inula japonicaThunb. [Asteraceae; Inulae Flos], 10 g - Raphanus raphanistrum subsp. sativus (L.) Schmalh. [Brassicaceae; Raphani Semen Praeparatum], 15 g | | | | | |  |
| Li F et al., 2022 | Hewei Jiangni Decoction (HWJND) | Granules | Beijing Kangrentang Pharmaceutical Co., Ltd | Not reported | No modifications reported | Not reported | |
|  | Composition:   - Scutellaria baicalensis Georgi [Lamiaceae; Scutellariae Radix], 9 g - Coptis chinensis Franch., Coptis deltoidea C.Y.Cheng & P.K.Hsiao, or Coptis teeta Wall. [Ranunculaceae; Coptidis Rhizoma], 6 g - Zingiber officinale Roscoe [Zingiberaceae; Zingiberis Rhizoma Recens], 9 g - Pinellia ternata (Thunb.) Makino [Araceae; Pinelliae Rhizoma], 9 g - Fritillaria thunbergii Miq. [Liliaceae; Fritillariae Thunbergii Bulbus], 9 g - Taraxacum mongolicum Hand.-Mazz. or Taraxacum borealisinense Kitam. [Asteraceae; Taraxaci Herba], 9 g - Gentiana scabra Bunge, Gentiana triflora Pall., Gentiana manshurica Kitag., or Gentiana crassa subsp. rigescens(Franch. ex Hemsl.) Halda [Gentianaceae; Gentianae Radix], 9 g - Citrus × aurantium L. or Citrus sinensis (L.) Osbeck [Rutaceae; Aurantii Fructus Immaturus], 9 g - Trichosanthes kirilowii Maxim. or Trichosanthes rosthornii Harms [Cucurbitaceae; Trichosanthis Radix], 9 g - Glycyrrhiza glabra L., Glycyrrhiza uralensis Fisch. ex DC., or Glycyrrhiza inflata Batalin [Fabaceae; Glycyrrhizae Radix et Rhizoma], 3 g | | | | | |  |
| Li HL et al., 2023 | Siniyulianzheshi (SNYLZS)  Decoction | Herbal  Decoction | Department of Pharmacy (Beijing Tongrentang herbal products) | Prepared as a decoction (400 mL/day) | No modifications reported | Not reported | |
|  | Composition:   - Bupleurum chinense DC. or Bupleurum scorzonerifolium Willd. [Apiaceae; Bupleuri Radix Praeparata cum Aceto], 12 g - Citrus × aurantium L. or Citrus sinensis (L.) Osbeck [Rutaceae; Aurantii Fructus Immaturus Praeparatus], 15 g - Paeonia lactiflora Pall. or Paeonia veitchii Lynch [Paeoniaceae; Paeoniae Radix Rubra], 30 g - Glycyrrhiza glabra L., Glycyrrhiza uralensis Fisch. ex DC., or Glycyrrhiza inflata Batalin [Fabaceae; Glycyrrhizae Radix et Rhizoma], 9 g - Coptis chinensis Franch., Coptis deltoidea C.Y.Cheng & P.K.Hsiao, or Coptis teeta Wall. [Ranunculaceae; Coptidis Rhizoma Praeparata], 9 g - Tetradium ruticarpum (A.Juss.) T.G.Hartley [Rutaceae; Evodiae Fructus], 3 g - Pentanema britannica (L.) D.Gut.Larr., Santos-Vicente, Anderb., E.Rico & M.M.Mart.Ort. or Inula japonica Thunb. [Asteraceae; Inulae Flos], 15 g - Hematite (mineral-derived medicinal material, Corundum group, mainly containing Fe₂O₃) [Not applicable; Hematitum Crudum], 30 g | | | | | |  |
| Shih YS et al., 2019 | Wu Chu Yu Tang (WCYT) | Concentrated granules | Sun Ten Pharmaceutical Co., Ltd. | Herbs decocted with boiling water, filtered, and granulated | No modifications reported | Authenticated; HPLC fingerprint available | |
|  | Composition:   - Tetradium ruticarpum (A.Juss.) T.G.Hartley [Rutaceae; Evodiae Fructus] - Panax ginseng C.A.Mey. [Araliaceae; Ginseng Radix et Rhizoma] - Ziziphus jujuba Mill. [Rhamnaceae; Jujubae Fructus] - Zingiber officinale Roscoe [Zingiberaceae; Zingiberis Rhizoma Recens] | | | | | |  |
| Song S et al., 2024 | Banxia Houpu Decoction | Herbal  Decoction | Chinese Medicine Pharmacy, First Affiliated Hospital of Dalian Medical University | Decocted with water | No modifications | Not reported | |
|  | Composition:   - Pinellia ternata (Thunb.) Makino [Araceae; Pinelliae Rhizoma Praeparatum cum Zingibere], 15 g - Magnolia officinalis Rehder & E.H.Wilson or Magnolia officinalis var. biloba Rehder & E.H.Wilson [Magnoliaceae; Magnoliae Officinalis Cortex], 9 g - Wolfiporia cocos (F.A. Wolf) Ryvarden & Gilb. [Polyporaceae; Poria], 12 g - Zingiber officinale Roscoe [Zingiberaceae; Zingiberis Rhizoma Recens], 15 g - Perilla frutescens (L.) Britton [Lamiaceae; Perillae Folium], 6 g | | | | | |  |
| Takeuchi T et al., 2019 | Hangeshashinto (HST) | Not reported | Not reported | Not reported | No modifications reported | Not reported | |
|  | Composition:   - Pinellia ternata (Thunb.) Makino [Araceae; Pinelliae Rhizoma] - Ziziphus jujuba Mill. [Rhamnaceae; Jujubae Fructus] - Panax ginseng C.A.Mey. [Araliaceae; Ginseng Radix et Rhizoma] - Zingiber officinale Roscoe [Zingiberaceae; Zingiberis Rhizoma Praeparatum] - Coptis chinensis Franch., Coptis deltoidea C.Y.Cheng & P.K.Hsiao, or Coptis teeta Wall. [Ranunculaceae; Coptidis Rhizoma] - Scutellaria baicalensis Georgi [Lamiaceae; Scutellariae Radix] - Glycyrrhiza glabra L., Glycyrrhiza uralensis Fisch. ex DC., or Glycyrrhiza inflata Batalin [Fabaceae; Glycyrrhizae Radix et Rhizoma] | | | | | |  |
| Tominaga K et al., 2012 | Rikkunshito | Extract granules | Tsumura Co., Ltd (Product number TJ-43) | Not reported | No modifications reported | Not reported | |
|  | Composition:   - Glycyrrhiza glabra L., Glycyrrhiza uralensis Fisch. ex DC., or Glycyrrhiza inflata Batalin [Fabaceae; Glycyrrhizae Radix et Rhizoma] - Zingiber officinale Roscoe [Zingiberaceae; Zingiberis Rhizoma] - Atractylodes chinensis (DC.) Koidz. or Atractylodes lancea (Thunb.) DC. [Asteraceae; Atractylodis Rhizoma] - Ziziphus jujuba Mill. [Rhamnaceae; Jujubae Fructus] - Citrus reticulata Blanco [Rutaceae; Citri Reticulatae Pericarpium] - Panax ginseng C.A.Mey. [Araliaceae; Ginseng Radix et Rhizoma] - Pinellia ternata (Thunb.) Makino [Araceae; Pinelliae Rhizoma] - Wolfiporia cocos (F.A. Wolf) Ryvarden & Gilb. [Polyporaceae; Poria] | | | | | |  |
| Tominaga K et al., 2014 | Rikkunshito | Extract granules | Tsumura Co., Ltd (Product number TJ-43) | Spray-dried hot-water extract | No modifications reported | 3D-HPLC fingerprint reported | |
|  | Composition:   - Glycyrrhiza glabra L., Glycyrrhiza uralensis Fisch. ex DC., or Glycyrrhiza inflata Batalin [Fabaceae; Glycyrrhizae Radix et Rhizoma] - Zingiber officinale Roscoe [Zingiberaceae; Zingiberis Rhizoma] - Atractylodes chinensis (DC.) Koidz. or Atractylodes lancea (Thunb.) DC. [Asteraceae; Atractylodis Rhizoma] - Ziziphus jujuba Mill. [Rhamnaceae; Jujubae Fructus] - Citrus reticulata Blanco [Rutaceae; Citri Reticulatae Pericarpium] - Panax ginseng C.A.Mey. [Araliaceae; Ginseng Radix et Rhizoma] - Pinellia ternata (Thunb.) Makino [Araceae; Pinelliae Rhizoma] - Wolfiporia cocos (F.A. Wolf) Ryvarden & Gilb. [Polyporaceae; Poria] | | | | | |  |
| Wang GM et al., 2020 | Shugan Weian (SGWA) Recipe | Herbal  Decoction | Not reported | Prepared as a decoction (200 mL/day) | No modifications reported | Not reported | |
|  | Composition:   - Pinellia ternata (Thunb.) Makino [Araceae; Pinelliae Rhizoma Praeparatum], 10 g - Zingiber officinale Roscoe [Zingiberaceae; Zingiberis Rhizoma], 3 g - Tetradium ruticarpum (A.Juss.) T.G.Hartley [Rutaceae; Evodiae Fructus], 1 g - Codonopsis pilosula (Franch.) Nannf. or Codonopsis pilosula subsp. tangshen (Oliv.) D.Y.Hong [Campanulaceae; Codonopsis Radix], 10 g - Wolfiporia cocos (F.A. Wolf) Ryvarden & Gilb. [Polyporaceae; Poria], 15 g - Atractylodes macrocephala Koidz. [Asteraceae; Atractylodis Macrocephalae Rhizoma Praeparata], 10 g - Bupleurum chinense DC. or Bupleurum scorzonerifolium Willd. [Apiaceae; Bupleuri Radix], 10 g - Corydalis yanhusuo (Y.H.Chou & Chun C.Hsu) W.T.Wang ex Z.Y.Su & C.Y.Wu [Papaveraceae; Corydalis Rhizoma], 10 g - Dolomiaea costus (Falc.) Kasana & A.K.Pandey [Asteraceae; Aucklandiae Radix Praeparata], 6 g - Bambusa tuldoides Munro, Sinocalamus beecheyanus (Munro) McClure var. pubescens P.F.Li, or Phyllostachys nigra(Lodd.) Munro var. henonis (Mitford) Stapf ex Rendle [Poaceae; Bambusae Caulis in Taeniam], 10 g - Pentanema britannica (L.) D.Gut.Larr., Santos-Vicente, Anderb., E.Rico & M.M.Mart.Ort. or Inula japonica Thunb. [Asteraceae; Inulae Flos], 15 g - Coptis chinensis Franch., Coptis deltoidea C.Y.Cheng & P.K.Hsiao, or Coptis teeta Wall. [Ranunculaceae; Coptidis Rhizoma], 3 g | | | | | |  |
| Wang J et al., 2023 | Chaishao Tongjian (CSTG) Prescription | Herbal  Decoction | Not reported | Prepared as a decoction (400 mL/day) | No modifications reported | Not reported | |
|  | Composition:   - Bupleurum chinense DC. or Bupleurum scorzonerifolium Willd. [Apiaceae; Bupleuri Radix], 9 g - Paeonia lactiflora Pall. [Paeoniaceae; Paeoniae Radix Alba], 12 g - Coptis chinensis Franch., Coptis deltoidea C.Y.Cheng & P.K.Hsiao, or Coptis teeta Wall. [Ranunculaceae; Coptidis Rhizoma], 3 g - Tetradium ruticarpum (A.Juss.) T.G.Hartley [Rutaceae; Evodiae Fructus], 3 g - Rehmannia glutinosa (Gaertn.) Libosch. ex DC. [Orobanchaceae; Rehmanniae Radix], 9 g - Achyranthes bidentata Blume [Amaranthaceae; Achyranthis Bidentatae Radix], 9 g - Citrus × aurantium L. [Rutaceae; Aurantii Fructus], 12 g - Arca subcrenata Lischke, Arca granosa Linnaeus, or Arca inflata Reeve (animal-derived medicinal material) [Arcidae; Arcae Concha Praeparata], 27 g - Pinellia ternata (Thunb.) Makino [Araceae; Pinelliae Rhizoma], 6 g - Magnolia officinalis Rehder & E.H.Wilson or Magnolia officinalis var. biloba Rehder & E.H.Wilson [Magnoliaceae; Magnoliae Officinalis Cortex], 12 g - Atractylodes macrocephala Koidz. [Asteraceae; Atractylodis Macrocephalae Rhizoma Praeparata], 12 g - Pseudostellaria heterophylla (Miq.) Pax [Caryophyllaceae; Pseudostellariae Radix], 12 g | | | | | |  |
| Yang YF et al., 2018 | Hezhong Jianpi (HZJP)  Decoction | Herbal  Decoction | Department of Pharmacy (Shanghai Municipal Hospital of Traditional Chinese Medicine) | Prepared as a decoction (200 mL/day) | No modifications reported | Not reported | |
|  | Composition:   - *Atractylodes macrocephala* Koidz. [Asteraceae; *Atractylodis Macrocephalae Rhizoma*], 9 g - *Eriobotrya japonica* (Thunb.) Lindl. [Rosaceae; *Eriobotryae Folium*], 9 g - *Platycodon grandiflorus* (Jacq.) A.DC. [Campanulaceae; *Platycodonis Radix*], 1 g | | | | | |  |
| Yu, 2017 | Banxia Houpu (BXHP) Decoction | Herbal  Decoction | Not reported | Prepared as a decoction (300 mL/day) | No modifications reported | Not reported | |
|  | Composition:   - Pinellia ternata (Thunb.) Makino [Araceae; Pinelliae Rhizoma] - Magnolia officinalis Rehder & E.H.Wilson or Magnolia officinalis var. biloba Rehder & E.H.Wilson [Magnoliaceae; Magnoliae Officinalis Cortex] - Wolfiporia cocos (F.A. Wolf) Ryvarden & Gilb. [Polyporaceae; Poria] - Zingiber officinale Roscoe [Zingiberaceae; Zingiberis Rhizoma Recens] - Perilla frutescens (L.) Britton [Lamiaceae; Perillae Folium] | | | | | |  |
| Zhang J et al., 2021 | JianpiQinghua granule (JQ granule) | Granules | Beijing Tcmages Pharmaceutical Co., Ltd. | Not reported | No modifications reported | HPLC quantified compounds reported | |
|  | Composition:   - Codonopsis pilosula (Franch.) Nannf. or Codonopsis pilosula subsp. tangshen (Oliv.) D.Y.Hong [Campanulaceae; Codonopsis Radix], 18 g - Atractylodes chinensis (DC.) Koidz. or Atractylodes lancea (Thunb.) DC. [Asteraceae; Atractylodis Rhizoma], 15 g - Perilla frutescens (L.) Britton [Lamiaceae; Perillae Folium], 12 g - Eupatorium fortunei Turcz. [Asteraceae; Eupatorii Herba], 12 g - Citrus × aurantium L. or Citrus sinensis (L.) Osbeck [Rutaceae; Aurantii Fructus], 12 g - Scutellaria baicalensis Georgi [Lamiaceae; Scutellariae Radix], 9 g - Coptis chinensis Franch., Coptis deltoidea C.Y.Cheng & P.K.Hsiao, or Coptis teeta Wall. [Ranunculaceae; Coptidis Rhizoma], 6 g - Sepiella maindroni de Rochebrune or Sepia esculenta Hoyle (animal-derived medicinal material) [Sepiidae; Sepiae Endoconcha], 30 g - Not applicable (fermented medicinal product) [Not applicable; Massa Medicata Fermentata], 15 g - Wurfbainia compacta (Sol. ex Maton) Škorničk. & A.D.Poulsen or Wurfbainia vera (Blackw.) Škorničk. & A.D.Poulsen [Zingiberaceae; Amomi Fructus Rotundus], 6 g | | | | | |  |
| Zhang X et al., 2024 | Shugan Hewei (SGHWX) granules | Granules | Jiangyin Tianjiang Pharmaceutical Co., Ltd., Jiangsu, China | Not reported | No modifications reported | Not reported | |
|  | Composition:   - Ziziphus jujuba Mill. var. spinosa (Bunge) Hu ex H.F.Chow [Rhamnaceae; Ziziphi Spinosae Semen] - Magnolia officinalis Rehder & E.H.Wilson or Magnolia officinalis var. biloba Rehder & E.H.Wilson [Magnoliaceae; Magnoliae Officinalis Cortex] - Coptis chinensis Franch., Coptis deltoidea C.Y.Cheng & P.K.Hsiao, or Coptis teeta Wall. [Ranunculaceae; Coptidis Rhizoma] - Tetradium ruticarpum (A.Juss.) T.G.Hartley [Rutaceae; Evodiae Fructus] - Zingiber officinale Roscoe [Zingiberaceae; Zingiberis Rhizoma] - Arca subcrenata Lischke, Arca granosa Linnaeus, or Arca inflata Reeve (animal-derived medicinal material) [Arcidae; Arcae Concha] - Pentanema britannica (L.) D.Gut.Larr., Santos-Vicente, Anderb., E.Rico & M.M.Mart.Ort. or Inula japonica Thunb. [Asteraceae; Inulae Flos] - Bupleurum chinense DC. or Bupleurum scorzonerifolium Willd. [Apiaceae; Bupleuri Radix] - Corydalis yanhusuo (Y.H.Chou & Chun C.Hsu) W.T.Wang ex Z.Y.Su & C.Y.Wu [Papaveraceae; Corydalis Rhizoma] - Cyperus rotundus L. [Cyperaceae; Cyperi Rhizoma] - Gardenia jasminoides J.Ellis [Rubiaceae; Gardeniae Fructus] - Citrus × aurantium L. or Citrus sinensis (L.) Osbeck [Rutaceae; Aurantii Fructus] - Polygonatum kingianum Collett & Hemsl., Polygonatum sibiricum Redouté, or Polygonatum cyrtonema Hua [Asparagaceae; Polygonati Rhizoma] | | | | | | |
| Zhang HK et al., 2021 | Banxia Houpu (ZBXHP) Decoction | Herbal  Decoction | Not reported | Prepared as a decoction (300 mL/day) | Symptom-based prescription modifications were reported. | Not reported | |
|  | Composition:   - Pinellia ternata (Thunb.) Makino [Araceae; Pinelliae Rhizoma], 15 g - Magnolia officinalis Rehder & E.H.Wilson or Magnolia officinalis var. biloba Rehder & E.H.Wilson [Magnoliaceae; Magnoliae Officinalis Cortex], 15 g - Wolfiporia cocos (F.A. Wolf) Ryvarden & Gilb. [Polyporaceae; Poria], 12 g - Cyperus rotundus L. [Cyperaceae; Cyperi Rhizoma], 10 g - Perilla frutescens (L.) Britton [Lamiaceae; Perillae Folium], 9 g - Zingiber officinale Roscoe [Zingiberaceae; Zingiberis Rhizoma Recens], 9 g - Citrus reticulata Blanco [Rutaceae; Citri Reticulatae Pericarpium], 9 g - Glycyrrhiza glabra L., Glycyrrhiza uralensis Fisch. ex DC., or Glycyrrhiza inflata Batalin [Fabaceae; Glycyrrhizae Radix et Rhizoma], 3 g | | | | | | |
| Zhang N et al., 2024 | hugan Hewei (SGHW) formula | Herbal  Decoction | Department of Pharmacy (Yueyang Hospital of Integrated Traditional Chinese and Western Medicine) | Prepared as a decoction (200 mL/day) | No modifications reported | Not reported | |
|  | Composition:   - Bupleurum chinense DC. or Bupleurum scorzonerifolium Willd. [Apiaceae; Bupleuri Radix], 9 g - Cyperus rotundus L. [Cyperaceae; Cyperi Rhizoma], 12 g - Citrus × aurantium L. [Rutaceae; Aurantii Fructus], 12 g - Pentanema britannica (L.) D.Gut.Larr., Santos-Vicente, Anderb., E.Rico & M.M.Mart.Ort. or Inula japonica Thunb. [Asteraceae; Inulae Caulis], 12 g - Hematite (mineral-derived medicinal material) [Not applicable; Hematitum Crudum], 15 g - Coptis chinensis Franch., Coptis deltoidea C.Y.Cheng & P.K.Hsiao, or Coptis teeta Wall. [Ranunculaceae; Coptidis Rhizoma], 3 g - Tetradium ruticarpum (A.Juss.) T.G.Hartley [Rutaceae; Evodiae Fructus], 3 g - Crataegus pinnatifida Bunge [Rosaceae; Crataegi Fructus Praeparatus], 9 g - Arca subcrenata Lischke, Arca granosa Linnaeus, or Arca inflata Reeve (animal-derived medicinal material) [Arcidae; Arcae Concha Praeparata], 30 g - Polygonatum kingianum Collett & Hemsl., Polygonatum sibiricum Redouté, or Polygonatum cyrtonema Hua [Asparagaceae; Polygonati Rhizoma], 15 g - Fritillaria thunbergii Miq. [Liliaceae; Fritillariae Thunbergii Bulbus], 9 g - Magnolia officinalis Rehder & E.H.Wilson or Magnolia officinalis var. biloba Rehder & E.H.Wilson [Magnoliaceae; Magnoliae Officinalis Cortex], 12 g - Zingiber officinale Roscoe [Zingiberaceae; Zingiberis Rhizoma Recens], 3 g | | | | | | |
| Zhou et al., 2022 | JianZhong Jiangni (JZJN) decoction | Herbal  Decoction | Not reported | Prepared as a decoction (400 mL/day) | Symptom-based prescription modifications were reported. | Not reported | |
|  | Composition:   - *Vincetoxicum bungei* (G.Don) Cai F.Zhang & G.W.Hu [Apocynaceae; *Cynanchi Paniculati Radix et Rhizoma*], 30 g - *Arca subcrenata* Lischke, *Arca granosa* Linnaeus, or *Arca inflata* Reeve (animal-derived medicinal material) [Arcidae; *Arcae Concha Praeparata*], 30 g - *Ostrea gigas* Thunberg, *Ostrea talienwhanensis* Crosse, or *Ostrea rivularis* Gould (animal-derived medicinal material) [Ostreidae; *Ostreae Concha Praeparata*], 30 g - *Sepiella maindroni* de Rochebrune or *Sepia esculenta* Hoyle (animal-derived medicinal material) [Sepiidae; *Sepiae Endoconcha*], 30 g - *Codonopsis pilosula* (Franch.) Nannf. or *Codonopsis pilosula* subsp. *tangshen* (Oliv.) D.Y.Hong [Campanulaceae; *Codonopsis Radix Praeparata*], 20 g - *Corydalis yanhusuo* (Y.H.Chou & Chun C.Hsu) W.T.Wang ex Z.Y.Su & C.Y.Wu [Papaveraceae; *Corydalis Rhizoma*], 20 g - *Atractylodes macrocephala* Koidz. [Asteraceae; *Atractylodis Macrocephalae Rhizoma Praeparata*], 10 g - *Wolfiporia cocos* (F.A. Wolf) Ryvarden & Gilb. [Polyporaceae; *Poria*], 10 g - *Pinellia ternata* (Thunb.) Makino [Araceae; *Pinelliae Rhizoma Praeparatum cum Zingibere*], 10 g - *Paeonia lactiflora* Pall. [Paeoniaceae; *Paeoniae Radix Alba*], 10 g - *Cyperus rotundus* L. [Cyperaceae; *Cyperi Rhizoma Praeparatum*], 10 g - *Pentanema britannica* (L.) D.Gut.Larr., Santos-Vicente, Anderb., E.Rico & M.M.Mart.Ort. or *Inula japonica* Thunb. [Asteraceae; *Inulae Flos*], 10 g - *Dalbergia odorifera* T.C.Chen [Fabaceae; *Dalbergiae Odoriferae Lignum*], 5 g - *Glycyrrhiza glabra* L., *Glycyrrhiza uralensis* Fisch. ex DC., or *Glycyrrhiza inflata* Batalin [Fabaceae; *Glycyrrhizae Radix et Rhizoma*], 3 g | | | | | | |

Composition and preparation information is presented as reported in the original RCTs; unavailable information is indicated as “Not reported.” Botanical scientific names, author citations, and families were verified using the Medicinal Plant Names Services (MPNS) and Plants of the World Online (POWO) in accordance with the ConPhyMP and Four Pillars guidelines. Pharmacopoeial drug names follow the Pharmacopoeia of the People’s Republic of China (ChP). Where the original clinical trials did not specify the exact source species among multiple pharmacopoeially accepted species, all applicable species are listed and separated by “or.” Detailed taxonomic information is also provided in the Appendix.

Abbreviations: HPLC, High-Performance Liquid Chromatography; 3D-HPLC, Three-Dimensional High-Performance Liquid Chromatography.

**Supplementary Table S3. Characteristics of CHM versus PPI comparisons**

| Study | PPI | Dosage and frequency | Treatment duration | Outcome measurement |
| --- | --- | --- | --- | --- |
| Jia ZL et al., 2023 | Any of the following PPIs: omeprazole, lansoprazole, pantoprazole, or rabeprazole | Omeprazole: 20–60 mg, twice daily Lansoprazole: 15–30 mg, Once daily in the morning  Pantoprazole: 40 mg, once daily before breakfast  Rabeprazole: 20 mg, once daily | 4 weeks | GERD-Q |
| Lai YL et al., 2019 | Pantoprazole | 40 mg, once daily | 8 weeks | RDQ |
| Li HL et al., 2023 | Rabeprazole | 20 mg, once daily | 8 weeks | RDQ |
| Wang GM et al., 2020 | Rabeprazole | 20 mg, once daily | 8 weeks | RDQ |
| Wang J et al., 2023 | Rabeprazole | 20 mg, once daily | 8 weeks | RDQ |
| Yang YF et al., 2018 | Omeprazole | 20 mg, twice daily | 8 weeks | RDQ |
| Zhang HK et al., 2021 | Omeprazole | 20 mg, twice daily | 9 weeks | GERD-Q |
| Zhou Y et al., 2022 | Rabeprazole | 20 mg, once daily | 4 weeks | RDQ |

**Supplementary Table S4. Assessment of potential effect modifiers for the transitivity assumption**

| Authors, year | GERD subtype | Age | Treatment comparison | Treatment duration | Outcome measure | Baseline symptom severity |
| --- | --- | --- | --- | --- | --- | --- |
| Cai ZZ et al., 2024 | GERD | C: 51.19 ± 12.08  I: 52.68 ± 10.4 | CHM vs PPI + mosapride citrate | 8 weeks | RDQ | C: 21.77 ± 4.26  I: 22.19 ± 3.85 |
| Jia ZL et al., 2023 | GERD | C: 73.4 ± 4.8  70.1 ± 3.5 | CHM vs PPI | 4 weeks then follow-up after 3 months | GERD-Q | C: 8.25 ± 0.95  I: 8.32 ± 1.12 |
| Lai YL et al., 2019 | NERD | C: 47.9 ± 12.4  I: 45.6 ± 13.2 | CHM vs PPI | 8 weeks | RDQ | C: 17.66±3.37  I: 18.42±3.81 |
| Li Z et al., 2021 | GERD | C: 50.48 ± 11.71 I: 50.30 ± 10.38 | CHM vs PPI | 4 weeks, then follow-up after 1 and 3 months | GERD-Q | C: 10.10 ± 1.64  I: 10.53 ± 2.01 |
| Li HL et al., 2023 | GERD | C: 49.12 ± 15.25  I: 48.89 ± 16.72 | CHM vs PPI | 8 weeks, then follow-up after 4 weeks | RDQ | C: 20.96±6.02  I: 21.58±5.76 |
| Shih YS et al., 2019 | GERD | C: 46.95 ± 13.42 I: 46.03 ± 13.88 | CHM vs PPI | 4 weeks | RDQ/  GERD-Q | C: 21.59 ± 11.67/  4.92 ± 2.71  I: 24.88 ± 10.01/  6.15 ± 3.21 |
| Song S et al., 2024 | GERD | C: 54.55 ± 11.68 I: 55.09 ± 14.19 | CHM vs PPI + mosapride citrate | 2 weeks | FSSG | C: 16.21 ± 7.63  I: 18.90 ± 8.09 |
| Takeuchi T et al., 2019 | GERD | C: 60.8 ± 14.7  I: 59.2 ± 14.7 | CHM + reduced-dose PPI vs standard-dose PPI | 4 weeks | FSSG | C: 16.1 ± 6.2  I: 19.5 ± 9.6 |
| Tominaga K et al., 2012 | GERD | C: 63.6 (25–86) I: 64.5 (25–90) | CHM + PPI vs PPI | 4 weeks | FSSG | C: 15.5 ± 7.3  I: 17.6 ± 6.5 |
| Tominaga K et al., 2014 | NERD | C: 59.4 (22–83)  I: 62.1 (25–85) | CHM + PPI vs PPI | 8 weeks | FSSG | C: 16.5 ± 7.0  I: 17.1 ± 8.1 |
| Wang GM et al., 2020 | GERD | C: 54.62 ± 9.59  I: 53.66 ± 9.27 | CHM vs PPI | 8 weeks | RDQ | C: 21.31 ± 3.64  I: 20.75 ± 3.53 |
| Wang J et al., 2023 | NERD | C: 49.80 ± 13.10  I: 49.50 ± 12.50 | CHM vs PPI | 8 weeks | GERD-Q/  RDQ | C: 26.74 ± 5.20/  12.00 ± 1.97  I: 27.64 ± 4.68/  12.53 ± 1.93 |
| Yang YF et al., 2018 | GERD | C: 49.19 ± 11.75  I: 46.63 ± 13.80 | CHM vs PPI | 8 weeks | RDQ | C: 28.31±7.81  I: 26.60 ± 8.30 |
| Yu, 2017 | GERD | C: 47.62 ± 9.74  I: 48.71 ± 10.18 | CHM vs PPI + mosapride citrate | 8 weeks | GERD-Q | C: 18.27 ± 1.34  I: 17.98 ± 1.41 |
| Zhang J et al., 2021 | NERD | C: 46.54 ± 15.07  I: 45.45 ± 14.03 | CHM + reduced-dose PPI vs standard-dose PPI | 4 weeks, then follow-up after 2 weeks | GERD-Q | C: 10.26±2.08  I: 10.16±2.34 |
| Zhang HK et al., 2021 | GERD | C: 48. 54 ± 6. 72  I: 49. 16 ± 6. 81 | CHM vs PPI | 9 weeks | GERD-Q | C: 14.46 ± 1.75  I: 14.16 ± 1.62 |
| Zhang N et al., 2024 | GERD | C: 48.77 ± 11.96  I: 44.35 ± 14.25 | CHM vs PPI + mosapride citrate | 8 weeks | GERD-Q | C: 11.30 ±1.86  I: 11.88 ± 2.31 |
| Zhou Y et al., 2022 | NERD | C: 53.2 ± 10.4  I: 54.4 ± 10.8 | CHM vs PPI | 4 weeks | RDQ | C: 24. 3±5. 7  I: 25. 1±4. 9 |

**Supplementary Table S5. Botanical drug pairs.**

| **Botanic drug 1** | **Botanic drug 2** | **co_occurrence** |
| --- | --- | --- |
| **Zingiberis_Rhizoma** | Pinelliae_Rhizoma | 6 |
| **Glycyrrhizae_Radix_et_Rhizoma** | Pinelliae_Rhizoma | 6 |
| **Poria** | Pinelliae_Rhizoma | 6 |
| **Bupleuri_Radix** | Coptidis_Rhizoma | 6 |
| **Evodiae_Fructus** | Bupleuri_Radix | 6 |
| **Poria** | Zingiberis_Rhizoma | 5 |
| **Evodiae_Fructus** | Coptidis_Rhizoma | 5 |
| **Coptidis_Rhizoma** | Pinelliae_Rhizoma | 4 |
| **Bupleuri_Radix** | Pinelliae_Rhizoma | 4 |
| **Evodiae_Fructus** | Pinelliae_Rhizoma | 4 |
| **Cyperi_Rhizoma** | Pinelliae_Rhizoma | 4 |
| **Magnoliae_Officinalis_Cortex** | Pinelliae_Rhizoma | 4 |
| **Atractylodis_Macrocephalae_Rhizoma** | Pinelliae_Rhizoma | 4 |
| **Citri_Reticulatae_Pericarpium** | Pinelliae_Rhizoma | 4 |
| **Glycyrrhizae_Radix_et_Rhizoma** | Zingiberis_Rhizoma | 4 |
| **Inulae_Flos** | Coptidis_Rhizoma | 4 |
| **Inulae_Flos** | Bupleuri_Radix | 4 |
| **Aurantii Fructus** | Bupleuri_Radix | 4 |
| **Poria** | Glycyrrhizae_Radix_et_Rhizoma | 4 |
| **Aurantii Fructus** | Evodiae_Fructus | 4 |
| **Codonopsis_Radix** | Atractylodis_Macrocephalae_Rhizoma | 4 |
| **Codonopsis_Radix** | Pinelliae_Rhizoma | 3 |
| **Aurantii Fructus** | Pinelliae_Rhizoma | 3 |
| **Paeoniae Radix Alba** | Pinelliae_Rhizoma | 3 |
| **Coptidis_Rhizoma** | Zingiberis_Rhizoma | 3 |
| **Evodiae_Fructus** | Zingiberis_Rhizoma | 3 |
| **Magnoliae_Officinalis_Cortex** | Zingiberis_Rhizoma | 3 |
| **Citri_Reticulatae_Pericarpium** | Zingiberis_Rhizoma | 3 |
| **Ginseng Radix et Rhizoma** | Zingiberis_Rhizoma | 3 |
| **Jujubae Fructus** | Zingiberis_Rhizoma | 3 |
| **Magnoliae_Officinalis_Cortex** | Coptidis_Rhizoma | 3 |
| **Atractylodis_Macrocephalae_Rhizoma** | Coptidis_Rhizoma | 3 |
| **Codonopsis_Radix** | Coptidis_Rhizoma | 3 |
| **Aurantii Fructus** | Coptidis_Rhizoma | 3 |
| **Cyperi_Rhizoma** | Bupleuri_Radix | 3 |
| **Magnoliae_Officinalis_Cortex** | Bupleuri_Radix | 3 |
| **Atractylodis_Macrocephalae_Rhizoma** | Bupleuri_Radix | 3 |
| **Cyperi_Rhizoma** | Glycyrrhizae_Radix_et_Rhizoma | 3 |
| **Citri_Reticulatae_Pericarpium** | Glycyrrhizae_Radix_et_Rhizoma | 3 |
| **Cyperi_Rhizoma** | Evodiae_Fructus | 3 |
| **Magnoliae_Officinalis_Cortex** | Evodiae_Fructus | 3 |
| **Inulae_Flos** | Evodiae_Fructus | 3 |
| **Magnoliae_Officinalis_Cortex** | Cyperi_Rhizoma | 3 |
| **Aurantii Fructus** | Cyperi_Rhizoma | 3 |
| **Aurantii Fructus** | Magnoliae_Officinalis_Cortex | 3 |
| **Atractylodis_Macrocephalae_Rhizoma** | Poria | 3 |
| **Citri_Reticulatae_Pericarpium** | Poria | 3 |
| **Codonopsis_Radix** | Poria | 3 |
| **Inulae_Flos** | Atractylodis_Macrocephalae_Rhizoma | 3 |
| **Inulae_Flos** | Codonopsis_Radix | 3 |
| **Sepiae Endoconcha** | Codonopsis_Radix | 3 |
| **Jujubae Fructus** | Ginseng Radix et Rhizoma | 3 |
| **Inulae_Flos** | Pinelliae_Rhizoma | 2 |
| **Ginseng Radix et Rhizoma** | Pinelliae_Rhizoma | 2 |
| **Jujubae Fructus** | Pinelliae_Rhizoma | 2 |
| **Scutellariae Radix** | Pinelliae_Rhizoma | 2 |
| **Arcae Concha Praeparata** | Pinelliae_Rhizoma | 2 |
| **Corydalis Rhizoma** | Pinelliae_Rhizoma | 2 |
| **Perillae Folium** | Pinelliae_Rhizoma | 2 |
| **Sepiae Endoconcha** | Pinelliae_Rhizoma | 2 |
| **Ostreae Concha Praeparata** | Pinelliae_Rhizoma | 2 |
| **Bupleuri_Radix** | Zingiberis_Rhizoma | 2 |
| **Cyperi_Rhizoma** | Zingiberis_Rhizoma | 2 |
| **Atractylodis_Macrocephalae_Rhizoma** | Zingiberis_Rhizoma | 2 |
| **Codonopsis_Radix** | Zingiberis_Rhizoma | 2 |
| **Inulae_Flos** | Zingiberis_Rhizoma | 2 |
| **Perillae Folium** | Zingiberis_Rhizoma | 2 |
| **Glycyrrhizae_Radix_et_Rhizoma** | Coptidis_Rhizoma | 2 |
| **Cyperi_Rhizoma** | Coptidis_Rhizoma | 2 |
| **Aurantii_Fructus_Immaturus_Praeparatus** | Coptidis_Rhizoma | 2 |
| **Scutellariae Radix** | Coptidis_Rhizoma | 2 |
| **Arcae Concha Praeparata** | Coptidis_Rhizoma | 2 |
| **Paeoniae Radix Alba** | Coptidis_Rhizoma | 2 |
| **Hematitum** | Coptidis_Rhizoma | 2 |
| **Glycyrrhizae_Radix_et_Rhizoma** | Bupleuri_Radix | 2 |
| **Codonopsis_Radix** | Bupleuri_Radix | 2 |
| **Arcae Concha Praeparata** | Bupleuri_Radix | 2 |
| **Paeoniae Radix Alba** | Bupleuri_Radix | 2 |
| **Hematitum** | Bupleuri_Radix | 2 |
| **Paeoniae Radix Rubra** | Bupleuri_Radix | 2 |
| **Evodiae_Fructus** | Glycyrrhizae_Radix_et_Rhizoma | 2 |
| **Magnoliae_Officinalis_Cortex** | Glycyrrhizae_Radix_et_Rhizoma | 2 |
| **Atractylodis_Macrocephalae_Rhizoma** | Glycyrrhizae_Radix_et_Rhizoma | 2 |
| **Codonopsis_Radix** | Glycyrrhizae_Radix_et_Rhizoma | 2 |
| **Inulae_Flos** | Glycyrrhizae_Radix_et_Rhizoma | 2 |
| **Ginseng Radix et Rhizoma** | Glycyrrhizae_Radix_et_Rhizoma | 2 |
| **Jujubae Fructus** | Glycyrrhizae_Radix_et_Rhizoma | 2 |
| **Scutellariae Radix** | Glycyrrhizae_Radix_et_Rhizoma | 2 |
| **Sepiae Endoconcha** | Glycyrrhizae_Radix_et_Rhizoma | 2 |
| **Ostreae Concha Praeparata** | Glycyrrhizae_Radix_et_Rhizoma | 2 |
| **Paeoniae Radix Rubra** | Glycyrrhizae_Radix_et_Rhizoma | 2 |
| **Atractylodis_Macrocephalae_Rhizoma** | Evodiae_Fructus | 2 |
| **Arcae Concha Praeparata** | Evodiae_Fructus | 2 |
| **Paeoniae Radix Alba** | Evodiae_Fructus | 2 |
| **Hematitum** | Evodiae_Fructus | 2 |
| **Paeoniae Radix Rubra** | Evodiae_Fructus | 2 |
| **Poria** | Cyperi_Rhizoma | 2 |
| **Citri_Reticulatae_Pericarpium** | Cyperi_Rhizoma | 2 |
| **Inulae_Flos** | Cyperi_Rhizoma | 2 |
| **Arcae Concha Praeparata** | Cyperi_Rhizoma | 2 |
| **Paeoniae Radix Alba** | Cyperi_Rhizoma | 2 |
| **Ostreae Concha Praeparata** | Cyperi_Rhizoma | 2 |
| **Poria** | Magnoliae_Officinalis_Cortex | 2 |
| **Atractylodis_Macrocephalae_Rhizoma** | Magnoliae_Officinalis_Cortex | 2 |
| **Arcae Concha Praeparata** | Magnoliae_Officinalis_Cortex | 2 |
| **Paeoniae Radix Alba** | Magnoliae_Officinalis_Cortex | 2 |
| **Inulae_Flos** | Poria | 2 |
| **Corydalis Rhizoma** | Poria | 2 |
| **Perillae Folium** | Poria | 2 |
| **Sepiae Endoconcha** | Poria | 2 |
| **Arcae Concha Praeparata** | Atractylodis_Macrocephalae_Rhizoma | 2 |
| **Corydalis Rhizoma** | Atractylodis_Macrocephalae_Rhizoma | 2 |
| **Paeoniae Radix Alba** | Atractylodis_Macrocephalae_Rhizoma | 2 |
| **Sepiae Endoconcha** | Atractylodis_Macrocephalae_Rhizoma | 2 |
| **Perillae Folium** | Citri_Reticulatae_Pericarpium | 2 |
| **Corydalis Rhizoma** | Codonopsis_Radix | 2 |
| **Arcae Concha Praeparata** | Inulae_Flos | 2 |
| **Corydalis Rhizoma** | Inulae_Flos | 2 |
| **Hematitum** | Inulae_Flos | 2 |
| **Arcae Concha Praeparata** | Aurantii Fructus | 2 |
| **Paeoniae Radix Alba** | Aurantii Fructus | 2 |
| **Paeoniae Radix Alba** | Arcae Concha Praeparata | 2 |
| **Atractylodis Rhizoma** | Pinelliae_Rhizoma | 1 |
| **Gardeniae Fructus** | Pinelliae_Rhizoma | 1 |
| **Paeoniae Radix Rubra** | Pinelliae_Rhizoma | 1 |
| **Achyranthis Bidentatae Radix** | Pinelliae_Rhizoma | 1 |
| **Aucklandiae Radix Praeparatum** | Pinelliae_Rhizoma | 1 |
| **Bambusae Caulis in Taenia** | Pinelliae_Rhizoma | 1 |
| **Chuanxiong Rhizoma** | Pinelliae_Rhizoma | 1 |
| **Cimicifugae Rhizoma** | Pinelliae_Rhizoma | 1 |
| **Pseudostellariae Radix** | Pinelliae_Rhizoma | 1 |
| **Dalbergiae Odoriferae Lignum** | Pinelliae_Rhizoma | 1 |
| **Fossilia Ossis Mastodi** | Pinelliae_Rhizoma | 1 |
| **Hordei Fructus Germinatus Praeparatus** | Pinelliae_Rhizoma | 1 |
| **Perillae Caulis** | Pinelliae_Rhizoma | 1 |
| **Rehmanniae Radix** | Pinelliae_Rhizoma | 1 |
| **Cynanchi Paniculati Radix et Rhizoma** | Pinelliae_Rhizoma | 1 |
| **Aurantii Fructus** | Zingiberis_Rhizoma | 1 |
| **Scutellariae Radix** | Zingiberis_Rhizoma | 1 |
| **Arcae Concha Praeparata** | Zingiberis_Rhizoma | 1 |
| **Atractylodis Rhizoma** | Zingiberis_Rhizoma | 1 |
| **Corydalis Rhizoma** | Zingiberis_Rhizoma | 1 |
| **Sepiae Endoconcha** | Zingiberis_Rhizoma | 1 |
| **Fritillariae Thunbergii Bulbus** | Zingiberis_Rhizoma | 1 |
| **Hematitum** | Zingiberis_Rhizoma | 1 |
| **Polygonati Rhizoma** | Zingiberis_Rhizoma | 1 |
| **Aucklandiae Radix Praeparatum** | Zingiberis_Rhizoma | 1 |
| **Arcae Concha** | Zingiberis_Rhizoma | 1 |
| **Bambusae Caulis in Taenia** | Zingiberis_Rhizoma | 1 |
| **Cimicifugae Rhizoma** | Zingiberis_Rhizoma | 1 |
| **Crataegi Fructus Praeparatus** | Zingiberis_Rhizoma | 1 |
| **Hordei Fructus Germinatus Praeparatus** | Zingiberis_Rhizoma | 1 |
| **Ziziphi Spinosae Semen** | Zingiberis_Rhizoma | 1 |
| **Poria** | Coptidis_Rhizoma | 1 |
| **Ginseng Radix et Rhizoma** | Coptidis_Rhizoma | 1 |
| **Jujubae Fructus** | Coptidis_Rhizoma | 1 |
| **Atractylodis Rhizoma** | Coptidis_Rhizoma | 1 |
| **Corydalis Rhizoma** | Coptidis_Rhizoma | 1 |
| **Perillae Folium** | Coptidis_Rhizoma | 1 |
| **Sepiae Endoconcha** | Coptidis_Rhizoma | 1 |
| **Fritillariae Thunbergii Bulbus** | Coptidis_Rhizoma | 1 |
| **Paeoniae Radix Rubra** | Coptidis_Rhizoma | 1 |
| **Polygonati Rhizoma** | Coptidis_Rhizoma | 1 |
| **Achyranthis Bidentatae Radix** | Coptidis_Rhizoma | 1 |
| **Aucklandiae Radix Praeparatum** | Coptidis_Rhizoma | 1 |
| **Arcae Concha** | Coptidis_Rhizoma | 1 |
| **Bambusae Caulis in Taenia** | Coptidis_Rhizoma | 1 |
| **Pseudostellariae Radix** | Coptidis_Rhizoma | 1 |
| **Crataegi Fructus Praeparatus** | Coptidis_Rhizoma | 1 |
| **Eupatorii Herba** | Coptidis_Rhizoma | 1 |
| **Massa Medicata Fermentata** | Coptidis_Rhizoma | 1 |
| **Perillae Caulis** | Coptidis_Rhizoma | 1 |
| **Rehmanniae Radix** | Coptidis_Rhizoma | 1 |
| **Ziziphi Spinosae Semen** | Coptidis_Rhizoma | 1 |
| **Amomi Fructus Rotundus** | Coptidis_Rhizoma | 1 |
| **Raphani Semen Praeparatum** | Coptidis_Rhizoma | 1 |
| **Poria** | Bupleuri_Radix | 1 |
| **Citri_Reticulatae_Pericarpium** | Bupleuri_Radix | 1 |
| **Aurantii_Fructus_Immaturus_Praeparatus** | Bupleuri_Radix | 1 |
| **Scutellariae Radix** | Bupleuri_Radix | 1 |
| **Corydalis Rhizoma** | Bupleuri_Radix | 1 |
| **Fritillariae Thunbergii Bulbus** | Bupleuri_Radix | 1 |
| **Gardeniae Fructus** | Bupleuri_Radix | 1 |
| **Ostreae Concha Praeparata** | Bupleuri_Radix | 1 |
| **Polygonati Rhizoma** | Bupleuri_Radix | 1 |
| **Achyranthis Bidentatae Radix** | Bupleuri_Radix | 1 |
| **Aucklandiae Radix Praeparatum** | Bupleuri_Radix | 1 |
| **Arcae Concha** | Bupleuri_Radix | 1 |
| **Bambusae Caulis in Taenia** | Bupleuri_Radix | 1 |
| **Chuanxiong Rhizoma** | Bupleuri_Radix | 1 |
| **Pseudostellariae Radix** | Bupleuri_Radix | 1 |
| **Crataegi Fructus Praeparatus** | Bupleuri_Radix | 1 |
| **Fossilia Ossis Mastodi** | Bupleuri_Radix | 1 |
| **Perillae Caulis** | Bupleuri_Radix | 1 |
| **Rehmanniae Radix** | Bupleuri_Radix | 1 |
| **Ziziphi Spinosae Semen** | Bupleuri_Radix | 1 |
| **Raphani Semen Praeparatum** | Bupleuri_Radix | 1 |
| **Aurantii_Fructus_Immaturus_Praeparatus** | Glycyrrhizae_Radix_et_Rhizoma | 1 |
| **Aurantii Fructus** | Glycyrrhizae_Radix_et_Rhizoma | 1 |
| **Arcae Concha Praeparata** | Glycyrrhizae_Radix_et_Rhizoma | 1 |
| **Atractylodis Rhizoma** | Glycyrrhizae_Radix_et_Rhizoma | 1 |
| **Corydalis Rhizoma** | Glycyrrhizae_Radix_et_Rhizoma | 1 |
| **Paeoniae Radix Alba** | Glycyrrhizae_Radix_et_Rhizoma | 1 |
| **Perillae Folium** | Glycyrrhizae_Radix_et_Rhizoma | 1 |
| **Gardeniae Fructus** | Glycyrrhizae_Radix_et_Rhizoma | 1 |
| **Hematitum** | Glycyrrhizae_Radix_et_Rhizoma | 1 |
| **Chuanxiong Rhizoma** | Glycyrrhizae_Radix_et_Rhizoma | 1 |
| **Cimicifugae Rhizoma** | Glycyrrhizae_Radix_et_Rhizoma | 1 |
| **Dalbergiae Odoriferae Lignum** | Glycyrrhizae_Radix_et_Rhizoma | 1 |
| **Fossilia Ossis Mastodi** | Glycyrrhizae_Radix_et_Rhizoma | 1 |
| **Hordei Fructus Germinatus Praeparatus** | Glycyrrhizae_Radix_et_Rhizoma | 1 |
| **Cynanchi Paniculati Radix et Rhizoma** | Glycyrrhizae_Radix_et_Rhizoma | 1 |
| **Poria** | Evodiae_Fructus | 1 |
| **Citri_Reticulatae_Pericarpium** | Evodiae_Fructus | 1 |
| **Codonopsis_Radix** | Evodiae_Fructus | 1 |
| **Aurantii_Fructus_Immaturus_Praeparatus** | Evodiae_Fructus | 1 |
| **Ginseng Radix et Rhizoma** | Evodiae_Fructus | 1 |
| **Jujubae Fructus** | Evodiae_Fructus | 1 |
| **Scutellariae Radix** | Evodiae_Fructus | 1 |
| **Corydalis Rhizoma** | Evodiae_Fructus | 1 |
| **Fritillariae Thunbergii Bulbus** | Evodiae_Fructus | 1 |
| **Gardeniae Fructus** | Evodiae_Fructus | 1 |
| **Ostreae Concha Praeparata** | Evodiae_Fructus | 1 |
| **Polygonati Rhizoma** | Evodiae_Fructus | 1 |
| **Achyranthis Bidentatae Radix** | Evodiae_Fructus | 1 |
| **Aucklandiae Radix Praeparatum** | Evodiae_Fructus | 1 |
| **Arcae Concha** | Evodiae_Fructus | 1 |
| **Bambusae Caulis in Taenia** | Evodiae_Fructus | 1 |
| **Chuanxiong Rhizoma** | Evodiae_Fructus | 1 |
| **Pseudostellariae Radix** | Evodiae_Fructus | 1 |
| **Crataegi Fructus Praeparatus** | Evodiae_Fructus | 1 |
| **Fossilia Ossis Mastodi** | Evodiae_Fructus | 1 |
| **Perillae Caulis** | Evodiae_Fructus | 1 |
| **Rehmanniae Radix** | Evodiae_Fructus | 1 |
| **Ziziphi Spinosae Semen** | Evodiae_Fructus | 1 |
| **Atractylodis_Macrocephalae_Rhizoma** | Cyperi_Rhizoma | 1 |
| **Codonopsis_Radix** | Cyperi_Rhizoma | 1 |
| **Scutellariae Radix** | Cyperi_Rhizoma | 1 |
| **Corydalis Rhizoma** | Cyperi_Rhizoma | 1 |
| **Perillae Folium** | Cyperi_Rhizoma | 1 |
| **Sepiae Endoconcha** | Cyperi_Rhizoma | 1 |
| **Fritillariae Thunbergii Bulbus** | Cyperi_Rhizoma | 1 |
| **Gardeniae Fructus** | Cyperi_Rhizoma | 1 |
| **Hematitum** | Cyperi_Rhizoma | 1 |
| **Paeoniae Radix Rubra** | Cyperi_Rhizoma | 1 |
| **Polygonati Rhizoma** | Cyperi_Rhizoma | 1 |
| **Arcae Concha** | Cyperi_Rhizoma | 1 |
| **Chuanxiong Rhizoma** | Cyperi_Rhizoma | 1 |
| **Crataegi Fructus Praeparatus** | Cyperi_Rhizoma | 1 |
| **Dalbergiae Odoriferae Lignum** | Cyperi_Rhizoma | 1 |
| **Fossilia Ossis Mastodi** | Cyperi_Rhizoma | 1 |
| **Perillae Caulis** | Cyperi_Rhizoma | 1 |
| **Cynanchi Paniculati Radix et Rhizoma** | Cyperi_Rhizoma | 1 |
| **Ziziphi Spinosae Semen** | Cyperi_Rhizoma | 1 |
| **Citri_Reticulatae_Pericarpium** | Magnoliae_Officinalis_Cortex | 1 |
| **Codonopsis_Radix** | Magnoliae_Officinalis_Cortex | 1 |
| **Inulae_Flos** | Magnoliae_Officinalis_Cortex | 1 |
| **Perillae Folium** | Magnoliae_Officinalis_Cortex | 1 |
| **Sepiae Endoconcha** | Magnoliae_Officinalis_Cortex | 1 |
| **Fritillariae Thunbergii Bulbus** | Magnoliae_Officinalis_Cortex | 1 |
| **Hematitum** | Magnoliae_Officinalis_Cortex | 1 |
| **Polygonati Rhizoma** | Magnoliae_Officinalis_Cortex | 1 |
| **Achyranthis Bidentatae Radix** | Magnoliae_Officinalis_Cortex | 1 |
| **Arcae Concha** | Magnoliae_Officinalis_Cortex | 1 |
| **Cimicifugae Rhizoma** | Magnoliae_Officinalis_Cortex | 1 |
| **Pseudostellariae Radix** | Magnoliae_Officinalis_Cortex | 1 |
| **Crataegi Fructus Praeparatus** | Magnoliae_Officinalis_Cortex | 1 |
| **Hordei Fructus Germinatus Praeparatus** | Magnoliae_Officinalis_Cortex | 1 |
| **Perillae Caulis** | Magnoliae_Officinalis_Cortex | 1 |
| **Rehmanniae Radix** | Magnoliae_Officinalis_Cortex | 1 |
| **Ziziphi Spinosae Semen** | Magnoliae_Officinalis_Cortex | 1 |
| **Ginseng Radix et Rhizoma** | Poria | 1 |
| **Jujubae Fructus** | Poria | 1 |
| **Arcae Concha Praeparata** | Poria | 1 |
| **Atractylodis Rhizoma** | Poria | 1 |
| **Paeoniae Radix Alba** | Poria | 1 |
| **Ostreae Concha Praeparata** | Poria | 1 |
| **Aucklandiae Radix Praeparatum** | Poria | 1 |
| **Bambusae Caulis in Taenia** | Poria | 1 |
| **Cimicifugae Rhizoma** | Poria | 1 |
| **Dalbergiae Odoriferae Lignum** | Poria | 1 |
| **Hordei Fructus Germinatus Praeparatus** | Poria | 1 |
| **Cynanchi Paniculati Radix et Rhizoma** | Poria | 1 |
| **Aurantii Fructus** | Atractylodis_Macrocephalae_Rhizoma | 1 |
| **Ostreae Concha Praeparata** | Atractylodis_Macrocephalae_Rhizoma | 1 |
| **Achyranthis Bidentatae Radix** | Atractylodis_Macrocephalae_Rhizoma | 1 |
| **Aucklandiae Radix Praeparatum** | Atractylodis_Macrocephalae_Rhizoma | 1 |
| **Bambusae Caulis in Taenia** | Atractylodis_Macrocephalae_Rhizoma | 1 |
| **Cimicifugae Rhizoma** | Atractylodis_Macrocephalae_Rhizoma | 1 |
| **Pseudostellariae Radix** | Atractylodis_Macrocephalae_Rhizoma | 1 |
| **Dalbergiae Odoriferae Lignum** | Atractylodis_Macrocephalae_Rhizoma | 1 |
| **Eriobotryae Folium** | Atractylodis_Macrocephalae_Rhizoma | 1 |
| **Hordei Fructus Germinatus Praeparatus** | Atractylodis_Macrocephalae_Rhizoma | 1 |
| **Rehmanniae Radix** | Atractylodis_Macrocephalae_Rhizoma | 1 |
| **Cynanchi Paniculati Radix et Rhizoma** | Atractylodis_Macrocephalae_Rhizoma | 1 |
| **Platycodonis Radix** | Atractylodis_Macrocephalae_Rhizoma | 1 |
| **Raphani Semen Praeparatum** | Atractylodis_Macrocephalae_Rhizoma | 1 |
| **Aurantii Fructus** | Citri_Reticulatae_Pericarpium | 1 |
| **Ginseng Radix et Rhizoma** | Citri_Reticulatae_Pericarpium | 1 |
| **Jujubae Fructus** | Citri_Reticulatae_Pericarpium | 1 |
| **Scutellariae Radix** | Citri_Reticulatae_Pericarpium | 1 |
| **Atractylodis Rhizoma** | Citri_Reticulatae_Pericarpium | 1 |
| **Gardeniae Fructus** | Citri_Reticulatae_Pericarpium | 1 |
| **Ostreae Concha Praeparata** | Citri_Reticulatae_Pericarpium | 1 |
| **Paeoniae Radix Rubra** | Citri_Reticulatae_Pericarpium | 1 |
| **Chuanxiong Rhizoma** | Citri_Reticulatae_Pericarpium | 1 |
| **Fossilia Ossis Mastodi** | Citri_Reticulatae_Pericarpium | 1 |
| **Aurantii_Fructus_Immaturus_Praeparatus** | Codonopsis_Radix | 1 |
| **Scutellariae Radix** | Codonopsis_Radix | 1 |
| **Arcae Concha Praeparata** | Codonopsis_Radix | 1 |
| **Atractylodis Rhizoma** | Codonopsis_Radix | 1 |
| **Paeoniae Radix Alba** | Codonopsis_Radix | 1 |
| **Perillae Folium** | Codonopsis_Radix | 1 |
| **Ostreae Concha Praeparata** | Codonopsis_Radix | 1 |
| **Aucklandiae Radix Praeparatum** | Codonopsis_Radix | 1 |
| **Bambusae Caulis in Taenia** | Codonopsis_Radix | 1 |
| **Cimicifugae Rhizoma** | Codonopsis_Radix | 1 |
| **Dalbergiae Odoriferae Lignum** | Codonopsis_Radix | 1 |
| **Eupatorii Herba** | Codonopsis_Radix | 1 |
| **Hordei Fructus Germinatus Praeparatus** | Codonopsis_Radix | 1 |
| **Massa Medicata Fermentata** | Codonopsis_Radix | 1 |
| **Cynanchi Paniculati Radix et Rhizoma** | Codonopsis_Radix | 1 |
| **Amomi Fructus Rotundus** | Codonopsis_Radix | 1 |
| **Raphani Semen Praeparatum** | Codonopsis_Radix | 1 |
| **Aurantii_Fructus_Immaturus_Praeparatus** | Inulae_Flos | 1 |
| **Aurantii Fructus** | Inulae_Flos | 1 |
| **Paeoniae Radix Alba** | Inulae_Flos | 1 |
| **Sepiae Endoconcha** | Inulae_Flos | 1 |
| **Fritillariae Thunbergii Bulbus** | Inulae_Flos | 1 |
| **Ostreae Concha Praeparata** | Inulae_Flos | 1 |
| **Paeoniae Radix Rubra** | Inulae_Flos | 1 |
| **Polygonati Rhizoma** | Inulae_Flos | 1 |
| **Aucklandiae Radix Praeparatum** | Inulae_Flos | 1 |
| **Arcae Concha** | Inulae_Flos | 1 |
| **Bambusae Caulis in Taenia** | Inulae_Flos | 1 |
| **Crataegi Fructus Praeparatus** | Inulae_Flos | 1 |
| **Dalbergiae Odoriferae Lignum** | Inulae_Flos | 1 |
| **Cynanchi Paniculati Radix et Rhizoma** | Inulae_Flos | 1 |
| **Ziziphi Spinosae Semen** | Inulae_Flos | 1 |
| **Raphani Semen Praeparatum** | Inulae_Flos | 1 |
| **Scutellariae Radix** | Aurantii_Fructus_Immaturus_Praeparatus | 1 |
| **Atractylodis Rhizoma** | Aurantii_Fructus_Immaturus_Praeparatus | 1 |
| **Perillae Folium** | Aurantii_Fructus_Immaturus_Praeparatus | 1 |
| **Sepiae Endoconcha** | Aurantii_Fructus_Immaturus_Praeparatus | 1 |
| **Hematitum** | Aurantii_Fructus_Immaturus_Praeparatus | 1 |
| **Paeoniae Radix Rubra** | Aurantii_Fructus_Immaturus_Praeparatus | 1 |
| **Eupatorii Herba** | Aurantii_Fructus_Immaturus_Praeparatus | 1 |
| **Massa Medicata Fermentata** | Aurantii_Fructus_Immaturus_Praeparatus | 1 |
| **Amomi Fructus Rotundus** | Aurantii_Fructus_Immaturus_Praeparatus | 1 |
| **Scutellariae Radix** | Aurantii Fructus | 1 |
| **Fritillariae Thunbergii Bulbus** | Aurantii Fructus | 1 |
| **Gardeniae Fructus** | Aurantii Fructus | 1 |
| **Hematitum** | Aurantii Fructus | 1 |
| **Ostreae Concha Praeparata** | Aurantii Fructus | 1 |
| **Paeoniae Radix Rubra** | Aurantii Fructus | 1 |
| **Polygonati Rhizoma** | Aurantii Fructus | 1 |
| **Achyranthis Bidentatae Radix** | Aurantii Fructus | 1 |
| **Arcae Concha** | Aurantii Fructus | 1 |
| **Chuanxiong Rhizoma** | Aurantii Fructus | 1 |
| **Pseudostellariae Radix** | Aurantii Fructus | 1 |
| **Crataegi Fructus Praeparatus** | Aurantii Fructus | 1 |
| **Fossilia Ossis Mastodi** | Aurantii Fructus | 1 |
| **Perillae Caulis** | Aurantii Fructus | 1 |
| **Rehmanniae Radix** | Aurantii Fructus | 1 |
| **Ziziphi Spinosae Semen** | Aurantii Fructus | 1 |
| **Scutellariae Radix** | Ginseng Radix et Rhizoma | 1 |
| **Atractylodis Rhizoma** | Ginseng Radix et Rhizoma | 1 |
| **Scutellariae Radix** | Jujubae Fructus | 1 |
| **Atractylodis Rhizoma** | Jujubae Fructus | 1 |
| **Atractylodis Rhizoma** | Scutellariae Radix | 1 |
| **Perillae Folium** | Scutellariae Radix | 1 |
| **Sepiae Endoconcha** | Scutellariae Radix | 1 |
| **Gardeniae Fructus** | Scutellariae Radix | 1 |
| **Ostreae Concha Praeparata** | Scutellariae Radix | 1 |
| **Paeoniae Radix Rubra** | Scutellariae Radix | 1 |
| **Chuanxiong Rhizoma** | Scutellariae Radix | 1 |
| **Eupatorii Herba** | Scutellariae Radix | 1 |
| **Fossilia Ossis Mastodi** | Scutellariae Radix | 1 |
| **Massa Medicata Fermentata** | Scutellariae Radix | 1 |
| **Amomi Fructus Rotundus** | Scutellariae Radix | 1 |
| **Corydalis Rhizoma** | Arcae Concha Praeparata | 1 |
| **Sepiae Endoconcha** | Arcae Concha Praeparata | 1 |
| **Fritillariae Thunbergii Bulbus** | Arcae Concha Praeparata | 1 |
| **Hematitum** | Arcae Concha Praeparata | 1 |
| **Ostreae Concha Praeparata** | Arcae Concha Praeparata | 1 |
| **Polygonati Rhizoma** | Arcae Concha Praeparata | 1 |
| **Achyranthis Bidentatae Radix** | Arcae Concha Praeparata | 1 |
| **Arcae Concha** | Arcae Concha Praeparata | 1 |
| **Pseudostellariae Radix** | Arcae Concha Praeparata | 1 |
| **Crataegi Fructus Praeparatus** | Arcae Concha Praeparata | 1 |
| **Dalbergiae Odoriferae Lignum** | Arcae Concha Praeparata | 1 |
| **Rehmanniae Radix** | Arcae Concha Praeparata | 1 |
| **Cynanchi Paniculati Radix et Rhizoma** | Arcae Concha Praeparata | 1 |
| **Ziziphi Spinosae Semen** | Arcae Concha Praeparata | 1 |
| **Perillae Folium** | Atractylodis Rhizoma | 1 |
| **Sepiae Endoconcha** | Atractylodis Rhizoma | 1 |
| **Eupatorii Herba** | Atractylodis Rhizoma | 1 |
| **Massa Medicata Fermentata** | Atractylodis Rhizoma | 1 |
| **Amomi Fructus Rotundus** | Atractylodis Rhizoma | 1 |
| **Paeoniae Radix Alba** | Corydalis Rhizoma | 1 |
| **Sepiae Endoconcha** | Corydalis Rhizoma | 1 |
| **Ostreae Concha Praeparata** | Corydalis Rhizoma | 1 |
| **Aucklandiae Radix Praeparatum** | Corydalis Rhizoma | 1 |
| **Bambusae Caulis in Taenia** | Corydalis Rhizoma | 1 |
| **Dalbergiae Odoriferae Lignum** | Corydalis Rhizoma | 1 |
| **Cynanchi Paniculati Radix et Rhizoma** | Corydalis Rhizoma | 1 |
| **Sepiae Endoconcha** | Paeoniae Radix Alba | 1 |
| **Ostreae Concha Praeparata** | Paeoniae Radix Alba | 1 |
| **Achyranthis Bidentatae Radix** | Paeoniae Radix Alba | 1 |
| **Pseudostellariae Radix** | Paeoniae Radix Alba | 1 |
| **Dalbergiae Odoriferae Lignum** | Paeoniae Radix Alba | 1 |
| **Perillae Caulis** | Paeoniae Radix Alba | 1 |
| **Rehmanniae Radix** | Paeoniae Radix Alba | 1 |
| **Cynanchi Paniculati Radix et Rhizoma** | Paeoniae Radix Alba | 1 |
| **Sepiae Endoconcha** | Perillae Folium | 1 |
| **Eupatorii Herba** | Perillae Folium | 1 |
| **Massa Medicata Fermentata** | Perillae Folium | 1 |
| **Amomi Fructus Rotundus** | Perillae Folium | 1 |
| **Ostreae Concha Praeparata** | Sepiae Endoconcha | 1 |
| **Cimicifugae Rhizoma** | Sepiae Endoconcha | 1 |
| **Dalbergiae Odoriferae Lignum** | Sepiae Endoconcha | 1 |
| **Eupatorii Herba** | Sepiae Endoconcha | 1 |
| **Hordei Fructus Germinatus Praeparatus** | Sepiae Endoconcha | 1 |
| **Massa Medicata Fermentata** | Sepiae Endoconcha | 1 |
| **Cynanchi Paniculati Radix et Rhizoma** | Sepiae Endoconcha | 1 |
| **Amomi Fructus Rotundus** | Sepiae Endoconcha | 1 |
| **Hematitum** | Fritillariae Thunbergii Bulbus | 1 |
| **Polygonati Rhizoma** | Fritillariae Thunbergii Bulbus | 1 |
| **Arcae Concha** | Fritillariae Thunbergii Bulbus | 1 |
| **Crataegi Fructus Praeparatus** | Fritillariae Thunbergii Bulbus | 1 |
| **Ziziphi Spinosae Semen** | Fritillariae Thunbergii Bulbus | 1 |
| **Ostreae Concha Praeparata** | Gardeniae Fructus | 1 |
| **Paeoniae Radix Rubra** | Gardeniae Fructus | 1 |
| **Chuanxiong Rhizoma** | Gardeniae Fructus | 1 |
| **Fossilia Ossis Mastodi** | Gardeniae Fructus | 1 |
| **Paeoniae Radix Rubra** | Hematitum | 1 |
| **Polygonati Rhizoma** | Hematitum | 1 |
| **Arcae Concha** | Hematitum | 1 |
| **Crataegi Fructus Praeparatus** | Hematitum | 1 |
| **Ziziphi Spinosae Semen** | Hematitum | 1 |
| **Paeoniae Radix Rubra** | Ostreae Concha Praeparata | 1 |
| **Chuanxiong Rhizoma** | Ostreae Concha Praeparata | 1 |
| **Dalbergiae Odoriferae Lignum** | Ostreae Concha Praeparata | 1 |
| **Fossilia Ossis Mastodi** | Ostreae Concha Praeparata | 1 |
| **Cynanchi Paniculati Radix et Rhizoma** | Ostreae Concha Praeparata | 1 |
| **Chuanxiong Rhizoma** | Paeoniae Radix Rubra | 1 |
| **Fossilia Ossis Mastodi** | Paeoniae Radix Rubra | 1 |
| **Arcae Concha** | Polygonati Rhizoma | 1 |
| **Crataegi Fructus Praeparatus** | Polygonati Rhizoma | 1 |
| **Ziziphi Spinosae Semen** | Polygonati Rhizoma | 1 |
| **Pseudostellariae Radix** | Achyranthis Bidentatae Radix | 1 |
| **Rehmanniae Radix** | Achyranthis Bidentatae Radix | 1 |
| **Bambusae Caulis in Taenia** | Aucklandiae Radix Praeparatum | 1 |
| **Crataegi Fructus Praeparatus** | Arcae Concha | 1 |
| **Ziziphi Spinosae Semen** | Arcae Concha | 1 |
| **Fossilia Ossis Mastodi** | Chuanxiong Rhizoma | 1 |
| **Hordei Fructus Germinatus Praeparatus** | Cimicifugae Rhizoma | 1 |
| **Rehmanniae Radix** | Pseudostellariae Radix | 1 |
| **Ziziphi Spinosae Semen** | Crataegi Fructus Praeparatus | 1 |
| **Cynanchi Paniculati Radix et Rhizoma** | Dalbergiae Odoriferae Lignum | 1 |
| **Platycodonis Radix** | Eriobotryae Folium | 1 |
| **Massa Medicata Fermentata** | Eupatorii Herba | 1 |
| **Amomi Fructus Rotundus** | Eupatorii Herba | 1 |
| **Amomi Fructus Rotundus** | Massa Medicata Fermentata | 1 |

**Supplementary Table S6. CINeMA assessment results.**

| **comparison** | **Direct** | **Indirect** | **NMA** | **Within-study bias** | **Reporting bias** | **Indirectness** | **Imprecision** | **Heterogeneity** | **Incoherence** | **NMA confidence rating** |
| --- | --- | --- | --- | --- | --- | --- | --- | --- | --- | --- |
| **BXHP:CHPLGML** | - | 0.11 [-0.54, 0.76] | 0.11 [-0.54, 0.76] | Some concerns | Some concerns | -- | -- | -- | -- | Low (⊕⊕○○) |
| **BXHP:CSTG** | - | -0.66 [-1.32, 0.00] | -0.66 [-1.32, 0.00] | Some concerns | Some concerns | -- | -- | -- | -- | Low (⊕⊕○○) |
| **BXHP:HEJIANG** | - | -0.41 [-1.10, 0.27] | -0.41 [-1.10, 0.27] | Major concerns | Some concerns | -- | -- | -- | -- | Very low (⊕○○○) |
| **BXHP:HST** | - | -0.10 [-0.75, 0.54] | -0.10 [-0.75, 0.54] | Some concerns | Some concerns | -- | -- | -- | -- | Low (⊕⊕○○) |
| **BXHP:HZJP** | - | -0.17 [-0.79, 0.44] | -0.17 [-0.79, 0.44] | No concerns | Some concerns | -- | -- | -- | -- | Moderate (⊕⊕⊕○) |
| **BXHP:JQ** | - | 0.03 [-0.49, 0.55] | 0.03 [-0.49, 0.55] | No concerns | Some concerns | -- | -- | -- | -- | Moderate (⊕⊕⊕○) |
| **BXHP:JZJN** | - | -0.48 [-1.16, 0.21] | -0.48 [-1.16, 0.21] | Major concerns | Some concerns | -- | -- | -- | -- | Very low (⊕○○○) |
| **BXHP:LZDBXHP** | - | -0.65 [-1.31, 0.01] | -0.65 [-1.31, 0.01] | Major concerns | Some concerns | -- | -- | -- | -- | Very low (⊕○○○) |
| **BXHP:MXD** | - | 0.06 [-0.44, 0.57] | 0.06 [-0.44, 0.57] | Some concerns | Some concerns | -- | -- | -- | -- | Low (⊕⊕○○) |
| **BXHP:PPI** | 0.31 [-0.13, 0.75] | - | 0.31 [-0.13, 0.75] | Major concerns | Some concerns | -- | -- | -- | -- | Very low (⊕○○○) |
| **BXHP:PPI_COMB** | 1.10 [0.60, 1.59] | - | 1.10 [0.60, 1.59] | Some concerns | Some concerns | -- | -- | -- | -- | Low (⊕⊕○○) |
| **BXHP:RIKKUNSHITO** | - | 0.17 [-0.33, 0.67] | 0.17 [-0.33, 0.67] | Major concerns | Some concerns | -- | -- | -- | -- | Very low (⊕○○○) |
| **BXHP:SGHW** | - | 0.45 [-0.22, 1.12] | 0.45 [-0.22, 1.12] | No concerns | Some concerns | -- | -- | -- | -- | Moderate (⊕⊕⊕○) |
| **BXHP:SGWA** | - | -0.80 [-1.39, -0.22] | -0.80 [-1.39, -0.22] | Major concerns | Some concerns | -- | -- | -- | -- | Very low (⊕○○○) |
| **BXHP:SNYLZS** | - | -0.46 [-1.10, 0.18] | -0.46 [-1.10, 0.18] | Major concerns | Some concerns | -- | -- | -- | -- | Very low (⊕○○○) |
| **BXHP:WCYT** | - | -0.36 [-0.99, 0.28] | -0.36 [-0.99, 0.28] | No concerns | Some concerns | -- | -- | -- | -- | Moderate (⊕⊕⊕○) |
| **BXHP:ZBXHP** | - | -2.29 [-3.02, -1.56] | -2.29 [-3.02, -1.56] | Major concerns | Some concerns | -- | -- | -- | -- | Very low (⊕○○○) |
| **CHPLGML:CSTG** | - | -0.77 [-1.70, 0.15] | -0.77 [-1.70, 0.15] | Some concerns | Some concerns | -- | -- | -- | -- | Low (⊕⊕○○) |
| **CHPLGML:HEJIANG** | - | -0.53 [-1.47, 0.41] | -0.53 [-1.47, 0.41] | Major concerns | Some concerns | -- | -- | -- | -- | Very low (⊕○○○) |
| **CHPLGML:HST** | - | -0.22 [-1.13, 0.70] | -0.22 [-1.13, 0.70] | Some concerns | Some concerns | -- | -- | -- | -- | Low (⊕⊕○○) |
| **CHPLGML:HZJP** | - | -0.29 [-1.18, 0.61] | -0.29 [-1.18, 0.61] | Major concerns | Some concerns | -- | -- | -- | -- | Very low (⊕○○○) |
| **CHPLGML:JQ** | - | -0.09 [-0.92, 0.75] | -0.09 [-0.92, 0.75] | Major concerns | Some concerns | -- | -- | -- | -- | Very low (⊕○○○) |
| **CHPLGML:JZJN** | - | -0.59 [-1.54, 0.35] | -0.59 [-1.54, 0.35] | Major concerns | Some concerns | -- | -- | -- | -- | Very low (⊕○○○) |
| **CHPLGML:LZDBXHP** | - | -0.76 [-1.69, 0.16] | -0.76 [-1.69, 0.16] | Major concerns | Some concerns | -- | -- | -- | -- | Very low (⊕○○○) |
| **CHPLGML:MXD** | - | -0.05 [-0.88, 0.77] | -0.05 [-0.88, 0.77] | Some concerns | Some concerns | -- | -- | -- | -- | Low (⊕⊕○○) |
| **CHPLGML:PPI** | - | 0.20 [-0.59, 0.98] | 0.20 [-0.59, 0.98] | Major concerns | Some concerns | -- | -- | -- | -- | Very low (⊕○○○) |
| **CHPLGML:PPI_COMB** | 0.98 [0.56, 1.41] | - | 0.98 [0.56, 1.41] | Major concerns | Some concerns | -- | -- | -- | -- | Very low (⊕○○○) |
| **CHPLGML:RIKKUNSHITO** | - | 0.05 [-0.77, 0.87] | 0.05 [-0.77, 0.87] | Major concerns | Some concerns | -- | -- | -- | -- | Very low (⊕○○○) |
| **CHPLGML:SGHW** | - | 0.34 [-0.28, 0.95] | 0.34 [-0.28, 0.95] | No concerns | Some concerns | -- | -- | -- | -- | Moderate (⊕⊕⊕○) |
| **CHPLGML:SGWA** | - | -0.92 [-1.79, -0.05] | -0.92 [-1.79, -0.05] | Major concerns | Some concerns | -- | -- | -- | -- | Very low (⊕○○○) |
| **CHPLGML:SNYLZS** | - | -0.57 [-1.48, 0.34] | -0.57 [-1.48, 0.34] | Major concerns | Some concerns | -- | -- | -- | -- | Very low (⊕○○○) |
| **CHPLGML:WCYT** | - | -0.47 [-1.38, 0.44] | -0.47 [-1.38, 0.44] | Major concerns | Some concerns | -- | -- | -- | -- | Very low (⊕○○○) |
| **CHPLGML:ZBXHP** | - | -2.41 [-3.38, -1.43] | -2.41 [-3.38, -1.43] | Major concerns | Some concerns | -- | -- | -- | -- | Very low (⊕○○○) |
| **CSTG:HEJIANG** | - | 0.24 [-0.47, 0.96] | 0.24 [-0.47, 0.96] | Some concerns | Some concerns | -- | -- | -- | -- | Low (⊕⊕○○) |
| **CSTG:HST** | - | 0.55 [-0.13, 1.24] | 0.55 [-0.13, 1.24] | Some concerns | Some concerns | -- | -- | -- | -- | Low (⊕⊕○○) |
| **CSTG:HZJP** | - | 0.48 [-0.17, 1.14] | 0.48 [-0.17, 1.14] | No concerns | Some concerns | -- | -- | -- | -- | Moderate (⊕⊕⊕○) |
| **CSTG:JQ** | - | 0.69 [0.12, 1.25] | 0.69 [0.12, 1.25] | No concerns | Some concerns | -- | -- | -- | -- | Moderate (⊕⊕⊕○) |
| **CSTG:JZJN** | - | 0.18 [-0.54, 0.90] | 0.18 [-0.54, 0.90] | Some concerns | Some concerns | -- | -- | -- | -- | Low (⊕⊕○○) |
| **CSTG:LZDBXHP** | - | 0.01 [-0.68, 0.70] | 0.01 [-0.68, 0.70] | Some concerns | Some concerns | -- | -- | -- | -- | Low (⊕⊕○○) |
| **CSTG:MXD** | - | 0.72 [0.17, 1.27] | 0.72 [0.17, 1.27] | Some concerns | Some concerns | -- | -- | -- | -- | Low (⊕⊕○○) |
| **CSTG:PPI** | 0.97 [0.48, 1.46] | - | 0.97 [0.48, 1.46] | Some concerns | Some concerns | -- | -- | -- | -- | Low (⊕⊕○○) |
| **CSTG:PPI_COMB** | - | 1.75 [0.93, 2.58] | 1.75 [0.93, 2.58] | Some concerns | Some concerns | -- | -- | -- | -- | Low (⊕⊕○○) |
| **CSTG:RIKKUNSHITO** | - | 0.83 [0.28, 1.37] | 0.83 [0.28, 1.37] | Some concerns | Some concerns | -- | -- | -- | -- | Low (⊕⊕○○) |
| **CSTG:SGHW** | - | 1.11 [0.17, 2.05] | 1.11 [0.17, 2.05] | Some concerns | Some concerns | -- | -- | -- | -- | Low (⊕⊕○○) |
| **CSTG:SGWA** | - | -0.15 [-0.77, 0.48] | -0.15 [-0.77, 0.48] | Some concerns | Some concerns | -- | -- | -- | -- | Low (⊕⊕○○) |
| **CSTG:SNYLZS** | - | 0.20 [-0.48, 0.88] | 0.20 [-0.48, 0.88] | Some concerns | Some concerns | -- | -- | -- | -- | Low (⊕⊕○○) |
| **CSTG:WCYT** | - | 0.30 [-0.37, 0.97] | 0.30 [-0.37, 0.97] | No concerns | Some concerns | -- | -- | -- | -- | Moderate (⊕⊕⊕○) |
| **CSTG:ZBXHP** | - | -1.64 [-2.40, -0.87] | -1.64 [-2.40, -0.87] | Some concerns | Some concerns | -- | -- | -- | -- | Low (⊕⊕○○) |
| **HEJIANG:HST** | - | 0.31 [-0.39, 1.02] | 0.31 [-0.39, 1.02] | Some concerns | Some concerns | -- | -- | -- | -- | Low (⊕⊕○○) |
| **HEJIANG:HZJP** | - | 0.24 [-0.44, 0.92] | 0.24 [-0.44, 0.92] | No concerns | Some concerns | -- | -- | -- | -- | Moderate (⊕⊕⊕○) |
| **HEJIANG:JQ** | - | 0.44 [-0.15, 1.04] | 0.44 [-0.15, 1.04] | No concerns | Some concerns | -- | -- | -- | -- | Moderate (⊕⊕⊕○) |
| **HEJIANG:JZJN** | - | -0.06 [-0.80, 0.68] | -0.06 [-0.80, 0.68] | Major concerns | Some concerns | -- | -- | -- | -- | Very low (⊕○○○) |
| **HEJIANG:LZDBXHP** | - | -0.23 [-0.95, 0.48] | -0.23 [-0.95, 0.48] | Major concerns | Some concerns | -- | -- | -- | -- | Very low (⊕○○○) |
| **HEJIANG:MXD** | - | 0.48 [-0.10, 1.06] | 0.48 [-0.10, 1.06] | Some concerns | Some concerns | -- | -- | -- | -- | Low (⊕⊕○○) |
| **HEJIANG:PPI** | 0.73 [0.20, 1.25] | - | 0.73 [0.20, 1.25] | Major concerns | Some concerns | -- | -- | -- | -- | Very low (⊕○○○) |
| **HEJIANG:PPI_COMB** | - | 1.51 [0.67, 2.35] | 1.51 [0.67, 2.35] | Major concerns | Some concerns | -- | -- | -- | -- | Very low (⊕○○○) |
| **HEJIANG:RIKKUNSHITO** | - | 0.58 [0.01, 1.16] | 0.58 [0.01, 1.16] | Major concerns | Some concerns | -- | -- | -- | -- | Very low (⊕○○○) |
| **HEJIANG:SGHW** | - | 0.86 [-0.09, 1.82] | 0.86 [-0.09, 1.82] | Major concerns | Some concerns | -- | -- | -- | -- | Very low (⊕○○○) |
| **HEJIANG:SGWA** | - | -0.39 [-1.04, 0.26] | -0.39 [-1.04, 0.26] | Major concerns | Some concerns | -- | -- | -- | -- | Very low (⊕○○○) |
| **HEJIANG:SNYLZS** | - | -0.04 [-0.74, 0.66] | -0.04 [-0.74, 0.66] | Major concerns | Some concerns | -- | -- | -- | -- | Very low (⊕○○○) |
| **HEJIANG:WCYT** | - | 0.06 [-0.64, 0.75] | 0.06 [-0.64, 0.75] | No concerns | Some concerns | -- | -- | -- | -- | Moderate (⊕⊕⊕○) |
| **HEJIANG:ZBXHP** | - | -1.88 [-2.66, -1.10] | -1.88 [-2.66, -1.10] | Major concerns | Some concerns | -- | -- | -- | -- | Very low (⊕○○○) |
| **HST:HZJP** | - | -0.07 [-0.71, 0.57] | -0.07 [-0.71, 0.57] | No concerns | Some concerns | -- | -- | -- | -- | Moderate (⊕⊕⊕○) |
| **HST:JQ** | - | 0.13 [-0.42, 0.68] | 0.13 [-0.42, 0.68] | No concerns | Some concerns | -- | -- | -- | -- | Moderate (⊕⊕⊕○) |
| **HST:JZJN** | - | -0.37 [-1.08, 0.33] | -0.37 [-1.08, 0.33] | Some concerns | Some concerns | -- | -- | -- | -- | Low (⊕⊕○○) |
| **HST:LZDBXHP** | - | -0.55 [-1.22, 0.13] | -0.55 [-1.22, 0.13] | Some concerns | Some concerns | -- | -- | -- | -- | Low (⊕⊕○○) |
| **HST:MXD** | - | 0.17 [-0.37, 0.70] | 0.17 [-0.37, 0.70] | Some concerns | Some concerns | -- | -- | -- | -- | Low (⊕⊕○○) |
| **HST:PPI** | 0.41 [-0.06, 0.89] | - | 0.41 [-0.06, 0.89] | Some concerns | Some concerns | -- | -- | -- | -- | Low (⊕⊕○○) |
| **HST:PPI_COMB** | - | 1.20 [0.39, 2.01] | 1.20 [0.39, 2.01] | Some concerns | Some concerns | -- | -- | -- | -- | Low (⊕⊕○○) |
| **HST:RIKKUNSHITO** | - | 0.27 [-0.26, 0.80] | 0.27 [-0.26, 0.80] | Some concerns | Some concerns | -- | -- | -- | -- | Low (⊕⊕○○) |
| **HST:SGHW** | - | 0.55 [-0.37, 1.48] | 0.55 [-0.37, 1.48] | Some concerns | Some concerns | -- | -- | -- | -- | Low (⊕⊕○○) |
| **HST:SGWA** | - | -0.70 [-1.31, -0.09] | -0.70 [-1.31, -0.09] | Some concerns | Some concerns | -- | -- | -- | -- | Low (⊕⊕○○) |
| **HST:SNYLZS** | - | -0.36 [-1.02, 0.31] | -0.36 [-1.02, 0.31] | Some concerns | Some concerns | -- | -- | -- | -- | Low (⊕⊕○○) |
| **HST:WCYT** | - | -0.26 [-0.91, 0.40] | -0.26 [-0.91, 0.40] | No concerns | Some concerns | -- | -- | -- | -- | Moderate (⊕⊕⊕○) |
| **HST:ZBXHP** | - | -2.19 [-2.94, -1.44] | -2.19 [-2.94, -1.44] | Some concerns | Some concerns | -- | -- | -- | -- | Low (⊕⊕○○) |
| **HZJP:JQ** | - | 0.20 [-0.31, 0.72] | 0.20 [-0.31, 0.72] | No concerns | Some concerns | -- | -- | -- | -- | Moderate (⊕⊕⊕○) |
| **HZJP:JZJN** | - | -0.30 [-0.98, 0.38] | -0.30 [-0.98, 0.38] | No concerns | Some concerns | -- | -- | -- | -- | Moderate (⊕⊕⊕○) |
| **HZJP:LZDBXHP** | - | -0.47 [-1.13, 0.18] | -0.47 [-1.13, 0.18] | No concerns | Some concerns | -- | -- | -- | -- | Moderate (⊕⊕⊕○) |
| **HZJP:MXD** | - | 0.24 [-0.26, 0.73] | 0.24 [-0.26, 0.73] | No concerns | Some concerns | -- | -- | -- | -- | Moderate (⊕⊕⊕○) |
| **HZJP:PPI** | 0.49 [0.05, 0.92] | - | 0.49 [0.05, 0.92] | No concerns | Some concerns | -- | -- | -- | -- | Moderate (⊕⊕⊕○) |
| **HZJP:PPI_COMB** | - | 1.27 [0.48, 2.06] | 1.27 [0.48, 2.06] | No concerns | Some concerns | -- | -- | -- | -- | Moderate (⊕⊕⊕○) |
| **HZJP:RIKKUNSHITO** | - | 0.34 [-0.15, 0.84] | 0.34 [-0.15, 0.84] | No concerns | Some concerns | -- | -- | -- | -- | Moderate (⊕⊕⊕○) |
| **HZJP:SGHW** | - | 0.62 [-0.28, 1.53] | 0.62 [-0.28, 1.53] | No concerns | Some concerns | -- | -- | -- | -- | Moderate (⊕⊕⊕○) |
| **HZJP:SGWA** | - | -0.63 [-1.21, -0.05] | -0.63 [-1.21, -0.05] | No concerns | Some concerns | -- | -- | -- | -- | Moderate (⊕⊕⊕○) |
| **HZJP:SNYLZS** | - | -0.28 [-0.92, 0.35] | -0.28 [-0.92, 0.35] | No concerns | Some concerns | -- | -- | -- | -- | Moderate (⊕⊕⊕○) |
| **HZJP:WCYT** | - | -0.19 [-0.82, 0.44] | -0.19 [-0.82, 0.44] | No concerns | Some concerns | -- | -- | -- | -- | Moderate (⊕⊕⊕○) |
| **HZJP:ZBXHP** | - | -2.12 [-2.84, -1.40] | -2.12 [-2.84, -1.40] | No concerns | Some concerns | -- | -- | -- | -- | Moderate (⊕⊕⊕○) |
| **JQ:JZJN** | - | -0.51 [-1.10, 0.09] | -0.51 [-1.10, 0.09] | No concerns | Some concerns | -- | -- | -- | -- | Moderate (⊕⊕⊕○) |
| **JQ:LZDBXHP** | - | -0.68 [-1.24, -0.11] | -0.68 [-1.24, -0.11] | No concerns | Some concerns | -- | -- | -- | -- | Moderate (⊕⊕⊕○) |
| **JQ:MXD** | - | 0.03 [-0.34, 0.41] | 0.03 [-0.34, 0.41] | No concerns | Some concerns | -- | -- | -- | -- | Moderate (⊕⊕⊕○) |
| **JQ:PPI** | 0.28 [0.00, 0.56] | - | 0.28 [0.00, 0.56] | No concerns | Some concerns | -- | -- | -- | -- | Moderate (⊕⊕⊕○) |
| **JQ:PPI_COMB** | - | 1.07 [0.35, 1.78] | 1.07 [0.35, 1.78] | No concerns | Some concerns | -- | -- | -- | -- | Moderate (⊕⊕⊕○) |
| **JQ:RIKKUNSHITO** | - | 0.14 [-0.23, 0.51] | 0.14 [-0.23, 0.51] | No concerns | Some concerns | -- | -- | -- | -- | Moderate (⊕⊕⊕○) |
| **JQ:SGHW** | - | 0.42 [-0.43, 1.27] | 0.42 [-0.43, 1.27] | No concerns | Some concerns | -- | -- | -- | -- | Moderate (⊕⊕⊕○) |
| **JQ:SGWA** | - | -0.83 [-1.31, -0.36] | -0.83 [-1.31, -0.36] | No concerns | Some concerns | -- | -- | -- | -- | Moderate (⊕⊕⊕○) |
| **JQ:SNYLZS** | - | -0.49 [-1.03, 0.06] | -0.49 [-1.03, 0.06] | No concerns | Some concerns | -- | -- | -- | -- | Moderate (⊕⊕⊕○) |
| **JQ:WCYT** | - | -0.39 [-0.93, 0.15] | -0.39 [-0.93, 0.15] | No concerns | Some concerns | -- | -- | -- | -- | Moderate (⊕⊕⊕○) |
| **JQ:ZBXHP** | - | -2.32 [-2.97, -1.68] | -2.32 [-2.97, -1.68] | No concerns | Some concerns | -- | -- | -- | -- | Moderate (⊕⊕⊕○) |
| **JZJN:LZDBXHP** | - | -0.17 [-0.89, 0.55] | -0.17 [-0.89, 0.55] | Major concerns | Some concerns | -- | -- | -- | -- | Very low (⊕○○○) |
| **JZJN:MXD** | - | 0.54 [-0.04, 1.12] | 0.54 [-0.04, 1.12] | Some concerns | Some concerns | -- | -- | -- | -- | Low (⊕⊕○○) |
| **JZJN:PPI** | 0.79 [0.26, 1.31] | - | 0.79 [0.26, 1.31] | Major concerns | Some concerns | -- | -- | -- | -- | Very low (⊕○○○) |
| **JZJN:PPI_COMB** | - | 1.57 [0.73, 2.42] | 1.57 [0.73, 2.42] | Major concerns | Some concerns | -- | -- | -- | -- | Very low (⊕○○○) |
| **JZJN:RIKKUNSHITO** | - | 0.65 [0.07, 1.22] | 0.65 [0.07, 1.22] | Major concerns | Some concerns | -- | -- | -- | -- | Very low (⊕○○○) |
| **JZJN:SGHW** | - | 0.93 [-0.03, 1.88] | 0.93 [-0.03, 1.88] | Major concerns | Some concerns | -- | -- | -- | -- | Very low (⊕○○○) |
| **JZJN:SGWA** | - | -0.33 [-0.98, 0.32] | -0.33 [-0.98, 0.32] | Major concerns | Some concerns | -- | -- | -- | -- | Very low (⊕○○○) |
| **JZJN:SNYLZS** | - | 0.02 [-0.68, 0.72] | 0.02 [-0.68, 0.72] | Major concerns | Some concerns | -- | -- | -- | -- | Very low (⊕○○○) |
| **JZJN:WCYT** | - | 0.12 [-0.58, 0.82] | 0.12 [-0.58, 0.82] | No concerns | Some concerns | -- | -- | -- | -- | Moderate (⊕⊕⊕○) |
| **JZJN:ZBXHP** | - | -1.82 [-2.60, -1.03] | -1.82 [-2.60, -1.03] | Major concerns | Some concerns | -- | -- | -- | -- | Very low (⊕○○○) |
| **LZDBXHP:MXD** | - | 0.71 [0.16, 1.26] | 0.71 [0.16, 1.26] | Some concerns | Some concerns | -- | -- | -- | -- | Low (⊕⊕○○) |
| **LZDBXHP:PPI** | 0.96 [0.47, 1.45] | - | 0.96 [0.47, 1.45] | Major concerns | Some concerns | -- | -- | -- | -- | Very low (⊕○○○) |
| **LZDBXHP:PPI_COMB** | - | 1.75 [0.92, 2.57] | 1.75 [0.92, 2.57] | Major concerns | Some concerns | -- | -- | -- | -- | Very low (⊕○○○) |
| **LZDBXHP:RIKKUNSHITO** | - | 0.82 [0.27, 1.36] | 0.82 [0.27, 1.36] | Major concerns | Some concerns | -- | -- | -- | -- | Very low (⊕○○○) |
| **LZDBXHP:SGHW** | - | 1.10 [0.16, 2.03] | 1.10 [0.16, 2.03] | Major concerns | Some concerns | -- | -- | -- | -- | Very low (⊕○○○) |
| **LZDBXHP:SGWA** | - | -0.16 [-0.78, 0.47] | -0.16 [-0.78, 0.47] | Major concerns | Some concerns | -- | -- | -- | -- | Very low (⊕○○○) |
| **LZDBXHP:SNYLZS** | - | 0.19 [-0.49, 0.87] | 0.19 [-0.49, 0.87] | Major concerns | Some concerns | -- | -- | -- | -- | Very low (⊕○○○) |
| **LZDBXHP:WCYT** | - | 0.29 [-0.38, 0.96] | 0.29 [-0.38, 0.96] | No concerns | Some concerns | -- | -- | -- | -- | Moderate (⊕⊕⊕○) |
| **LZDBXHP:ZBXHP** | - | -1.64 [-2.40, -0.88] | -1.64 [-2.40, -0.88] | Major concerns | Some concerns | -- | -- | -- | -- | Very low (⊕○○○) |
| **MXD:PPI** | 0.25 [-0.00, 0.50] | - | 0.25 [-0.00, 0.50] | Some concerns | Some concerns | -- | -- | -- | -- | Low (⊕⊕○○) |
| **MXD:PPI_COMB** | - | 1.03 [0.33, 1.74] | 1.03 [0.33, 1.74] | Some concerns | Some concerns | -- | -- | -- | -- | Low (⊕⊕○○) |
| **MXD:RIKKUNSHITO** | - | 0.11 [-0.24, 0.45] | 0.11 [-0.24, 0.45] | Some concerns | Some concerns | -- | -- | -- | -- | Low (⊕⊕○○) |
| **MXD:SGHW** | - | 0.39 [-0.45, 1.22] | 0.39 [-0.45, 1.22] | Some concerns | Some concerns | -- | -- | -- | -- | Low (⊕⊕○○) |
| **MXD:SGWA** | - | -0.87 [-1.33, -0.41] | -0.87 [-1.33, -0.41] | Some concerns | Some concerns | -- | -- | -- | -- | Low (⊕⊕○○) |
| **MXD:SNYLZS** | - | -0.52 [-1.05, 0.01] | -0.52 [-1.05, 0.01] | Some concerns | Some concerns | -- | -- | -- | -- | Low (⊕⊕○○) |
| **MXD:WCYT** | - | -0.42 [-0.94, 0.10] | -0.42 [-0.94, 0.10] | No concerns | Some concerns | -- | -- | -- | -- | Moderate (⊕⊕⊕○) |
| **MXD:ZBXHP** | - | -2.35 [-2.99, -1.72] | -2.35 [-2.99, -1.72] | Some concerns | Some concerns | -- | -- | -- | -- | Low (⊕⊕○○) |
| **PPI:PPI_COMB** | - | -0.79 [-1.45, -0.13] | -0.79 [-1.45, -0.13] | Some concerns | Some concerns | -- | -- | -- | -- | Low (⊕⊕○○) |
| **PPI:RIKKUNSHITO** | 0.14 [-0.10, 0.38] | - | 0.14 [-0.10, 0.38] | Some concerns | Some concerns | -- | -- | -- | -- | Low (⊕⊕○○) |
| **PPI:SGHW** | - | -0.14 [-0.94, 0.66] | -0.14 [-0.94, 0.66] | No concerns | Some concerns | -- | -- | -- | -- | Moderate (⊕⊕⊕○) |
| **PPI:SGWA** | 1.12 [0.73, 1.50] | - | 1.12 [0.73, 1.50] | Major concerns | Some concerns | -- | -- | -- | -- | Very low (⊕○○○) |
| **PPI:SNYLZS** | 0.77 [0.30, 1.24] | - | 0.77 [0.30, 1.24] | Major concerns | Some concerns | -- | -- | -- | -- | Very low (⊕○○○) |
| **PPI:WCYT** | 0.67 [0.21, 1.13] | - | 0.67 [0.21, 1.13] | No concerns | Some concerns | -- | -- | -- | -- | Moderate (⊕⊕⊕○) |
| **PPI:ZBXHP** | 2.60 [2.02, 3.19] | - | 2.60 [2.02, 3.19] | Major concerns | Some concerns | -- | -- | -- | -- | Very low (⊕○○○) |
| **PPI_COMB:RIKKUNSHITO** | - | -0.93 [-1.63, -0.23] | -0.93 [-1.63, -0.23] | Some concerns | Some concerns | -- | -- | -- | -- | Low (⊕⊕○○) |
| **PPI_COMB:SGHW** | -0.65 [-1.10, -0.20] | - | -0.65 [-1.10, -0.20] | No concerns | Some concerns | -- | -- | -- | -- | Moderate (⊕⊕⊕○) |
| **PPI_COMB:SGWA** | - | -1.90 [-2.67, -1.14] | -1.90 [-2.67, -1.14] | Major concerns | Some concerns | -- | -- | -- | -- | Very low (⊕○○○) |
| **PPI_COMB:SNYLZS** | - | -1.56 [-2.36, -0.75] | -1.56 [-2.36, -0.75] | Major concerns | Some concerns | -- | -- | -- | -- | Very low (⊕○○○) |
| **PPI_COMB:WCYT** | - | -1.46 [-2.26, -0.65] | -1.46 [-2.26, -0.65] | No concerns | Some concerns | -- | -- | -- | -- | Moderate (⊕⊕⊕○) |
| **PPI_COMB:ZBXHP** | - | -3.39 [-4.27, -2.51] | -3.39 [-4.27, -2.51] | Major concerns | Some concerns | -- | -- | -- | -- | Very low (⊕○○○) |
| **RIKKUNSHITO:SGHW** | - | 0.28 [-0.55, 1.12] | 0.28 [-0.55, 1.12] | No concerns | Some concerns | -- | -- | -- | -- | Moderate (⊕⊕⊕○) |
| **RIKKUNSHITO:SGWA** | - | -0.97 [-1.43, -0.52] | -0.97 [-1.43, -0.52] | Major concerns | Some concerns | -- | -- | -- | -- | Very low (⊕○○○) |
| **RIKKUNSHITO:SNYLZS** | - | -0.63 [-1.15, -0.10] | -0.63 [-1.15, -0.10] | Major concerns | Some concerns | -- | -- | -- | -- | Very low (⊕○○○) |
| **RIKKUNSHITO:WCYT** | - | -0.53 [-1.05, -0.01] | -0.53 [-1.05, -0.01] | No concerns | Some concerns | -- | -- | -- | -- | Moderate (⊕⊕⊕○) |
| **RIKKUNSHITO:ZBXHP** | - | -2.46 [-3.09, -1.83] | -2.46 [-3.09, -1.83] | Major concerns | Some concerns | -- | -- | -- | -- | Very low (⊕○○○) |
| **SGHW:SGWA** | - | -1.25 [-2.14, -0.37] | -1.25 [-2.14, -0.37] | Major concerns | Some concerns | -- | -- | -- | -- | Very low (⊕○○○) |
| **SGHW:SNYLZS** | - | -0.91 [-1.83, 0.02] | -0.91 [-1.83, 0.02] | Major concerns | Some concerns | -- | -- | -- | -- | Very low (⊕○○○) |
| **SGHW:WCYT** | - | -0.81 [-1.73, 0.11] | -0.81 [-1.73, 0.11] | No concerns | Some concerns | -- | -- | -- | -- | Moderate (⊕⊕⊕○) |
| **SGHW:ZBXHP** | - | -2.74 [-3.73, -1.76] | -2.74 [-3.73, -1.76] | Major concerns | Some concerns | -- | -- | -- | -- | Very low (⊕○○○) |
| **SGWA:SNYLZS** | - | 0.35 [-0.26, 0.95] | 0.35 [-0.26, 0.95] | Major concerns | Some concerns | -- | -- | -- | -- | Very low (⊕○○○) |
| **SGWA:WCYT** | - | 0.45 [-0.15, 1.04] | 0.45 [-0.15, 1.04] | No concerns | Some concerns | -- | -- | -- | -- | Moderate (⊕⊕⊕○) |
| **SGWA:ZBXHP** | - | -1.49 [-2.19, -0.79] | -1.49 [-2.19, -0.79] | Major concerns | Some concerns | -- | -- | -- | -- | Very low (⊕○○○) |
| **SNYLZS:WCYT** | - | 0.10 [-0.56, 0.75] | 0.10 [-0.56, 0.75] | No concerns | Some concerns | -- | -- | -- | -- | Moderate (⊕⊕⊕○) |
| **SNYLZS:ZBXHP** | - | -1.83 [-2.58, -1.09] | -1.83 [-2.58, -1.09] | Major concerns | Some concerns | -- | -- | -- | -- | Very low (⊕○○○) |
| **WCYT:ZBXHP** | - | -1.93 [-2.67, -1.19] | -1.93 [-2.67, -1.19] | No concerns | Some concerns | -- | -- | -- | -- | Moderate (⊕⊕⊕○) |

**Supplementary Figure S1. Forest plot of Chinese herbal medicine (CHM) treatment in Gastroesophageal reflux disease (GERD) across different outcome measurement scales.**

Abbreviations: RDQ, Reflux Diagnostic Questionnaire; FSSG, Frequency Scale for the Symptoms of GERD.

**Supplementary Figure S2. Forest plot of CHM treatment by GERD subtypes.**

**Supplementary Figure S3. Forest plot of CHM treatment in GERD by comparator types.**

**Supplementary Figure S4. Subgroup analysis by outcome measure in CHM versus PPI comparisons**

**Supplementary Figure S5. Subgroup analysis by treatment duration in CHM versus PPI comparisons**

**Supplementary Figure S6. Publication bias.**

| Outcome | No. of trials | Egger’s Intercept (95% CI) | t (df) | p-value |
| --- | --- | --- | --- | --- |
| GERD symptom scores | 18 | -0.72 (−1.47 to 0.06) | 3.70 (16) | 0.0019 |

**Supplementary Figure S7. Cumulative ranking probabilities of all interventions (SUCRA curves).**

These curves display the cumulative probability that each treatment ranks at or above a given position, based on the network meta-analysis.

Abbreviation: ZBXHP, Zhang’s Banxia Houpu Decoction; BXHP, Banxia Houpu Decoction; SNYLZS, Sinivulianzheshi Decoction; HZJP, Hezhong Jianpi Decoction; SGHW, Shugan Hewei Formula; JZJN, Jianzhong Jiangni Decoction; HST, Hangeshashinto; RKT, Rikkunshito; JQ, JianpiQinghua Granules; SGWA, Shugan Weian Recipe; CSTG, Chaishao Tongjian Prescription; CHPLGML, Chaihu Plus Longgu Muli Decoction; LZDBXHP, Modified Lizhong Decoction combined with Banxia Houpu Decoction; MXD, Modified Xiaochaihu Decoction; WCYT, Wuchuyu Tang; PPI, proton pump inhibitors; PPI_comb, proton pump inhibitors combined with mosapride.

**Supplementary Figure S8. Cumulative ranking probability curves for each intervention.**

Each panel shows the cumulative probability curve for a single treatment across all possible ranks (1 = best, N = worst), as derived from the network meta-analysis.

Abbreviations are the same as described in Supplementary Figure S5.

**Supplementary Figure S9. Sensitivity Analysis.**

Forest plot of pairwise meta-analysis comparing CHM to PPI after exclusion of the outlier study

**Supplementary Figure S10. Component-level coefficient estimates illustrated by a weighted bubble plot.**

The analysis includes only botanical drugs with a frequency of use greater than three (frequency > 3). Bubble size represents precision (1/SE), the x-axis indicates the frequency of botanical drug use across included prescriptions, and the y-axis represents the estimated effect (β) derived from the component contribution analysis.
